# New hydrogen-bonding organocatalysts: Chiral cyclophosphazanes and phosphorus amides as catalysts for asymmetric Michael additions

**DOI:** 10.3762/bjoc.10.18

**Published:** 2014-01-21

**Authors:** Helge Klare, Jörg M Neudörfl, Bernd Goldfuss

**Affiliations:** 1 Department of Chemistry, Universität zu Köln, Greinstrasse 4, D-50939 Köln, Germany, Fax: +49(0)221-470-5057

**Keywords:** DFT computations, hydrogen bonding, Michael addition, organocatalysis, phosphazanes

## Abstract

Ten novel hydrogen-bonding catalysts based on open-chain P^V^-amides of BINOL and chinchona alkaloids as well as three catalysts based on rigid *cis*-P^V^-cyclodiphosphazane amides of *N*^1^,*N*^1^-dimethylcyclohexane-1,2-diamine have been developed. Employed in the asymmetric Michael addition of 2-hydroxynaphthoquinone to β-nitrostyrene, the open-chain 9-*epi*-aminochinchona-based phosphorus amides show a high catalytic activity with almost quantitative yields of up to 98% and enantiomeric excesses of up to 51%. The cyclodiphosphazane catalysts show the same high activity and give improved enantiomeric excesses of up to 75%, thus representing the first successful application of a cyclodiphosphazane in enantioselective organocatalysis. DFT computations reveal high hydrogen-bonding strengths of cyclodiphosphazane P^V^-amides compared to urea-based catalysts. Experimental results and computations on the enantiodetermining step with *cis*-cyclodiphosphazane **14a** suggest a strong bidentate H-bond activation of the nitrostyrene substrate by the catalyst.

## Introduction

**Figure 1:**
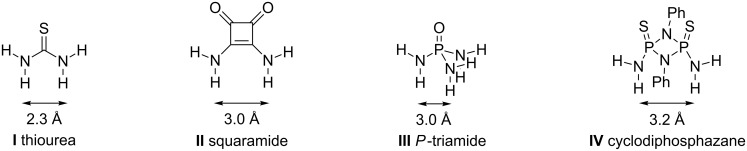
Thiourea, squaramide, *P*-triamide and cyclodiphosphazane with computed distances between H-atoms.

Organocatalysis has gained great impact in promoting highly enantioselective [1–4] and eco-friendly [5] reactions. Within the field of organocatalysis hydrogen-bonding (HB) catalysts represent an ever growing class [6–8]. The majority of non-specific hydrogen-bonding catalysts are based on the (thio)urea motif (**I**, Figure 1) [9–10]. More recently squaramides (**II**, Figure 1) have emerged as complementing motif in HB catalysis [11]. Other H-bonding motifs are less established, e.g. sulfonamides [12], urea-*N*-sulfoxides [13], guanines [14] as well as protonated catalysts such as ammonium [15], 2-aminopyridinium [16] and guanidinium [17] motifs.

Most catalysts can form two hydrogen bonds to a reactant, which further enhances their ability to activate and constrain it to a defined geometry. Introducing a new motif, Shea et al. have synthesized achiral tridentate (thio)phosphorus triamides and assessed their catalytic activity relative to the established (*m*-(CF_3_)_2_-Ph)_2_thiourea [18]. In the Friedel–Crafts reaction of *N*-methylindole with β-nitrostyrene and the Baylis–Hillman reaction of methyl acrylate with benzaldehyde the substituted triamide catalysts show a comparable or an even superior activity relative to the thiourea analogue. Recently, Gale et al. demonstrated that the same phosphoric triamides effectively act as anion transporters by hydrogen bonding [19]. These characteristics and the increased steric bulk of “3-D”-P^V^ compared to “2-D” urea or squaramides make phosphorus triamides excellent candidates for asymmetric (HB) organocatalysts. However, the tetrahedral structure of P^V^-amides also enables a higher degree of conformational freedom combined with a less rigid structure due to the low rotational barrier (6.5–10.0 kcal/mol) [20–21] of the P^V^–N bond. An ideal catalyst would thus combine the steric bulk of P^V^-amides with an improved rigidity. Cyclodiphosphazanes (**IV**, Figure 1) are saturated four-membered P_2_N_2_ heterocycles that can easily be synthesized with different substitution patterns on phosphorus and nitrogen from commercially available amines and PCl_3_. While achiral P^III/V^-cyclodiphospazanes have been studied as ligands in transition-metal chemistry [22–23], only two examples of cyclodiphosphazanes as catalysts in asymmetric reactions are known; Chakravarty et al. [24] tested an *ansa*-bridged BINOL-based P^V^-cyclodiphosphazane in the asymmetric reduction of acetophenone with BH_3_ (5–8% ee), while Gade et al. [25] recently introduced BINOL-based P^III^-cyclodiphosphazane ligands to transition-metal catalysis (up to 84% ee). We anticipated that by incorporation of a chiral amido-scaffold into bis(amido)cyclodiphosphazanes (*cis*-[R'NHP(S)(μ-NR)]_2_), bulky and conformationally constrained bidentate HB catalysts with an improved H-acidity should be accessible. HB interaction with donor substrates of the different structural motifs **I**–**IV** is expected to be dependent on the H-acidity, and on spacing and angle of the H-bond. The H-bond spacings for *N*,*N*'-dimethylthiourea and *N*,*N*'-dimethylsquaramide have been computed by the Takemoto and Rawal groups and were given to be 2.1 Å [9] and 2.7 Å [11], respectively. Computations of the H-bonding properties of both P-triamide **III** and cyclodiphosphazane **IV** and their comparison to the “classic” motifs **I** and **II** suggest a slightly larger spacing and are reported herein. We furthermore report the synthesis of chiral variants of the catalyst motifs **III** and **IV** and their successful application in the organocatalytic addition of 2-hydroxynaphthoquinone to β-nitrostyrene as a test reaction.

## Results and Discussion

### Computational assessment of HB strengths

**Figure 2:**
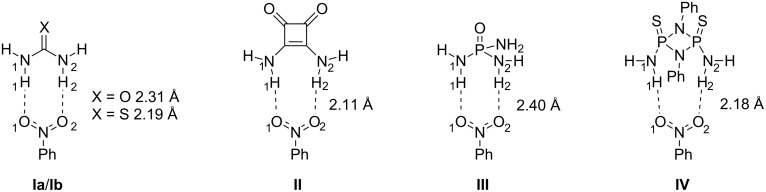
Urea, squaramide, *P*-triamide and cyclodiphosphazane coordinated to nitrobenzene, with the computed mean H-bond lengths (cf. Table 1).

To determine the relative strength of the hydrogen bonding, the interaction between unsubstituted HB motifs and nitrobenzene (**I**–**IV**, Figure 2) was computed. In the interaction between the proton donors and the proton-accepting nitro group, two bonding patterns are possible that involve either one or both oxygen atoms of the nitro moiety. Computation of either conformation revealed both oxygen atoms acting as proton acceptors to be most favored with all motifs. This is in agreement with previous computations carried out on the interaction of (thio)urea and HNO_2_ by Chen et al. [26].

Computations of the hydrogen-bonded complexes (Table 1) reveal a distinct trend for the non-phosphorus HB motifs; urea complex **Ia** exhibits the weakest ability to form hydrogen bonds to the nitro moiety with a bonding energy (Δ*E*) of 5.2 kcal/mol and a mean bond length NH_urea_–O_nitrobenzene_ of 2.31 Å. Thiourea complex **Ib** forms slightly stronger hydrogen bonds to the NO_2_ moiety with a bonding energy of 6.5 kcal/mol, which is also reflected in the shorter mean bond length of NH_thiourea_–O_nitrobenzene_ (2.19 Å). This is in accordance with the experimentally observed higher acidity [27] of thiourea over urea. Squaramide complex **II** forms the strongest hydrogen bonds of all computed motifs. The bond lengths NH_squaramide_–O_nitrobenzene_ are shortest with 2.11 Å while the bonding energy is highest (8.3 kcal/mol). The proposed ability of squaramides to form stronger hydrogen bonds than ureas is in agreement with the experimentally found higher acidity of squaramides [28]. The strength of hydrogen bonding is also thought to be dependent on the directionality of the hydrogen bonds, with an optimum of 180° [29]. This is supported by the computational results with smaller mean N–H–O angles for urea/thiourea (167°/173°) compared to squaramide (178°).

**Table 1:** Computational^a^ comparison of HB catalyst motifs (cf. Figure 2).

motif	NH–O [Å]	Δ*E* (kcal/mol)	angle (N1H1O1)	angle (N2H2O2)

**Ia** urea	2.31	5.2	168.3	166.8
**Ib** thiourea	2.19	6.5	174.3	172.9
**II** squaramide	2.11	8.3	178.7	177.6
**III** P^V^-triamide	2.40	4.2	169.5	170.1
**IV** phosphazane	2.18	7.2	165.1	165.0

^a^All computations performed with MARI-TPSS//def2-TZVP.

The open-chain P^V^-triamide complex **III** exhibits a weaker ability to form hydrogen bonds with a bonding energy of 4.2 kcal/mol (Table 1), which is slightly lower than that of the urea complex. The bonds are considerably elongated with a mean length of 2.40 Å. Tridentate binding was not computationally found. The cyclodiphosphazane complex **IV** on the other hand has a much more pronounced ability to form hydrogen bonds with a bonding energy of 7.2 kcal/mol, which exceeds even that of thiourea (6.5 kcal/mol), and a corresponding shorter bond length (2.18 Å), although the directionality of the hydrogen bonding is not optimal (165°). It is thus plausible, that P^V^-triamides and especially the P^V^-cyclodiphosphazane(s) can perform similarly or even better in activating a hydrogen-bond-acceptor than commonly employed (thio)ureas.

### Synthesis of chiral open-chain P^V^-amides

**Scheme 1:**
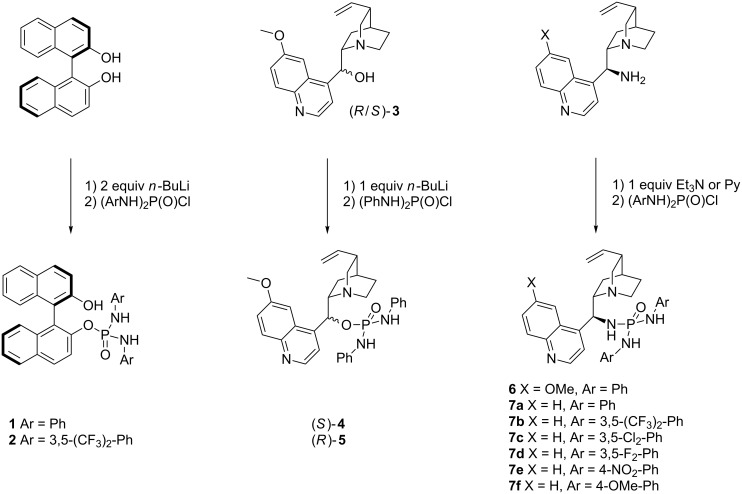
Chiral P^V^-amide catalysts based on BINOL and chinchona backbones.

We evaluated both BINOL and chinchona alkaloids as chiral backbones. BINOL is a well-established chiral motif [30], while chinchona alkaloids are sterically very demanding, can be readily converted to their 9-*epi*-amino derivatives and are well established in HB catalysis [31]. The P^V^-amide catalysts are efficiently accessible through addition of the lithium alkoxides (**1**,**2**,**4**,**5**, Scheme 1) or primary amines to *N*,*N*'-diarylphosphordiamido chloridates in THF or pyridine (**6**–**7f**, Scheme 1). The [(ArNH)_2_P(O)Cl] derivatives can be synthesized directly from POCl_3_ and the corresponding aniline derivatives in benzene under reflux [32] and were directly converted to the product without prior isolation in the case of **7b**–**f**.

**Scheme 2:**
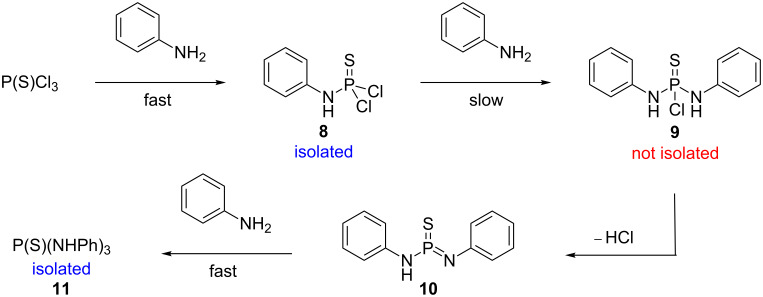
Exclusive formation of the mono- and trisubstituted product from thiophosphoryl chloride and aniline.

We also attempted to obtain the equivalent thiophosphoryl derivatives as their respective proton-donor capacity is higher [33]. Contrary to a literature protocol [34] synthesizing the [(ArNH)_2_P(S)Cl] precursors from PSCl_3_ and various anilines resulted in the exclusive formation of the mono- and trisubstituted products. The findings match those reported by Cremlyn et al. [35], who proposed a slow S_N_2(P) attack by amines on (phenyl)phosphoramidothioic dichloride(s) **8** due to the lower electrophilicity of the latter (Scheme 2). The intermediate [(ArNH)_2_P(S)Cl **9** undergoes a fast base-catalyzed E_1_cB-reaction to **10**, which then reacts with an additional equivalent of aniline to the triamide **11** via a metaphosphate-type intermediate. This prevents the isolation of the desired product **9**.

**Figure 3:**
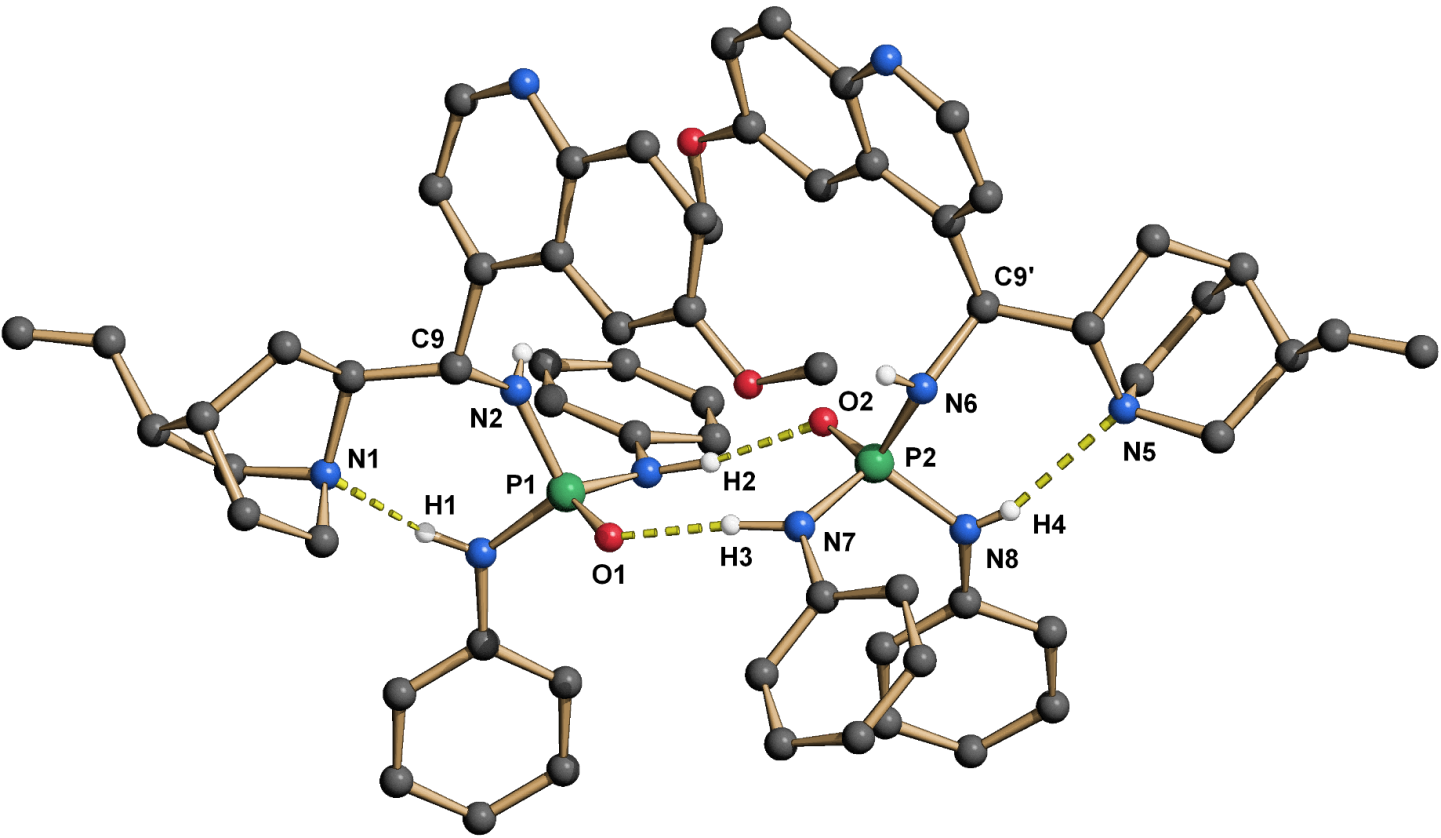
X-ray structure of **6**-dimer. The hydrogen atoms are omitted for clarity, except at all nitrogens.

**Figure 4:**
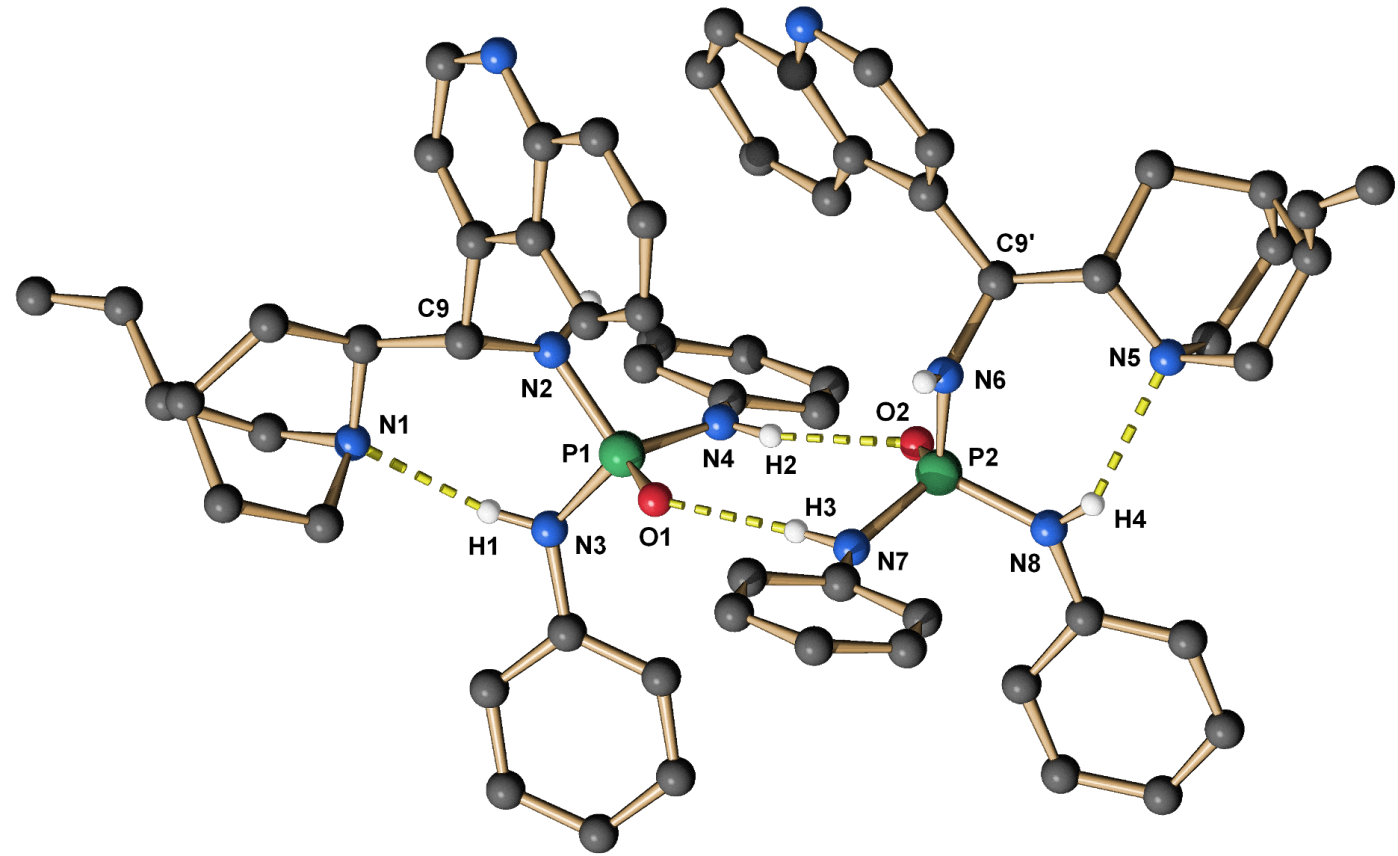
X-ray structure of **7a**-dimer. The hydrogen atoms are omitted for clarity, except at all nitrogens.

Hence, we synthesized a series of BINOL/chinchona-based oxophosphoric amide catalysts with different electron-donating/withdrawing substituents (Scheme 1). Crystals suitable for crystallography were obtained from catalysts **6** and **7a**. Both catalysts form dimers with short intermolecular hydrogen bonds between P1O1–H3 and P2O2–H2 (1.843/1.810 Å and 2.148/2.103 Å respectively, Figure 3 and Figure 4). In each case the second acidic NH proton H1 forms an intramolecular hydrogen bond to quinuclidin nitrogen N1, which is significantly shorter in **7a** (2.194/2.110 Å respectively). The intramolecular hydrogen bond is probably also the cause for the occurrence of conformational isomers when inverting the configuration at C9. While catalyst (*S*)-**4** solely exists as one conformer on the NMR timescale, its epimer (*R*)-**5** and the 9-*epi*-amino derivatives (*R*)-**6**/**7a**–**f** give unexpectedly complex ^1^H/^31^P NMR spectra in CDCl_3_/DMSO-*d*_6_ at room temperature. Signals that belong to any one proton split into two signals with a ratio of ~5:1/20:1 depending on the solvent. The reason for this is conformational isomerism, which was confirmed by DOSY and temperature-dependent NMR (Supporting Information File 1). The cause is probably the hindered rotation by the intramolecular hydrogen bond N1–H1.

### Synthesis of chiral P^V^-cyclodiphosphazane amides

**Scheme 3:**
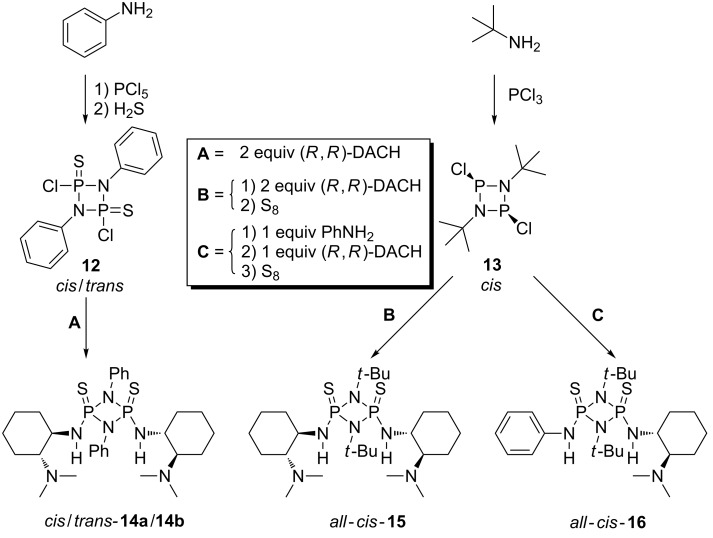
Synthesis of chiral cyclodiphosphazane catalysts **14a/b**, **15** and **16**.

In order to synthesize chiral cyclodiphosphazanes **14a**/**14b**, PCl_5_ was reacted with aniline to give [Cl_3_P(μ-NPh)]_2_ in a first step (route **A**, Scheme 3). The formation of [ClP(S)(μ-NPh)]_2_ **12** was accomplished by reaction with H_2_S by following a modified literature [36] protocol. Crystallization from benzene yields a mixture of the corresponding *cis*/*trans* isomers in a ratio of 5:1. Notably, the follow-up reaction of **12** with *N*^1^,*N*^1^-dimethyldiaminocyclohexane gives a 1:1 mixture of *cis*/*trans*-cyclodiphosphazane **14a**/**14b,** which does not reflect the distribution of *cis*/*trans*-isomers in the starting material. This indicates that the reaction does not strictly proceed via an S_N_2(P) mechanism.

**Figure 5:**
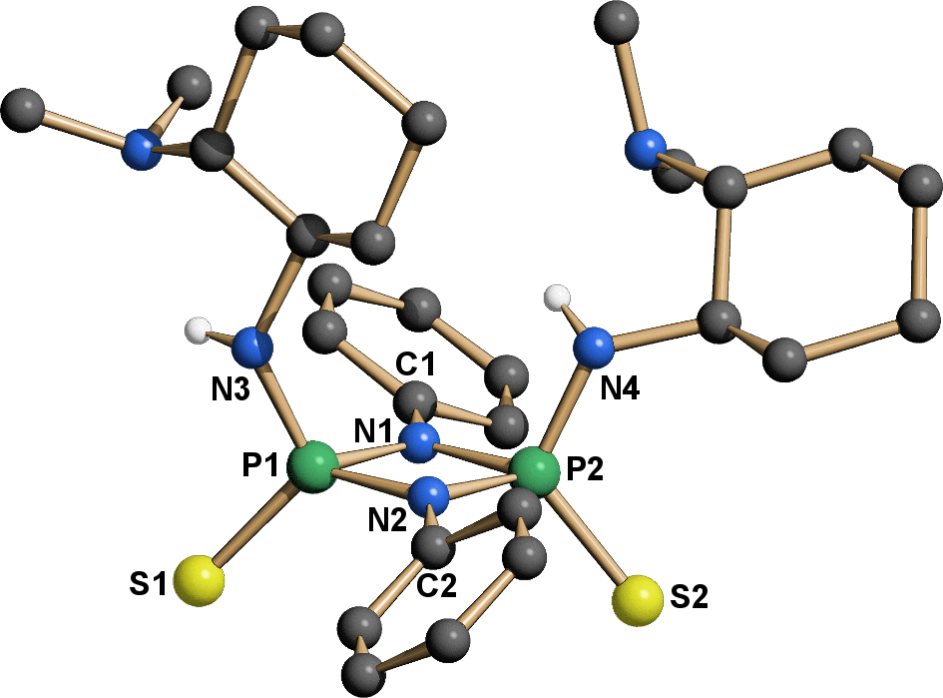
X-ray structure of **14a**. The hydrogen atoms are omitted for clarity, except at nitrogen.

The separation of the isomers was achieved by column chromatography over neutral grade V alumina. The use of a more active alumina resulted in a dramatic loss of the isolated yield. The configuration of the isolated isomers was unambiguously proven by crystallography (Figure 5). To date **14a** is the second [37] reported crystal structure of a *cis*-cylcodiphosphazane-2,4-disulfide with aromatic substituents on nitrogen. For all other known structures of this type the configuration is either not specified or *trans*.

**Figure 6:**
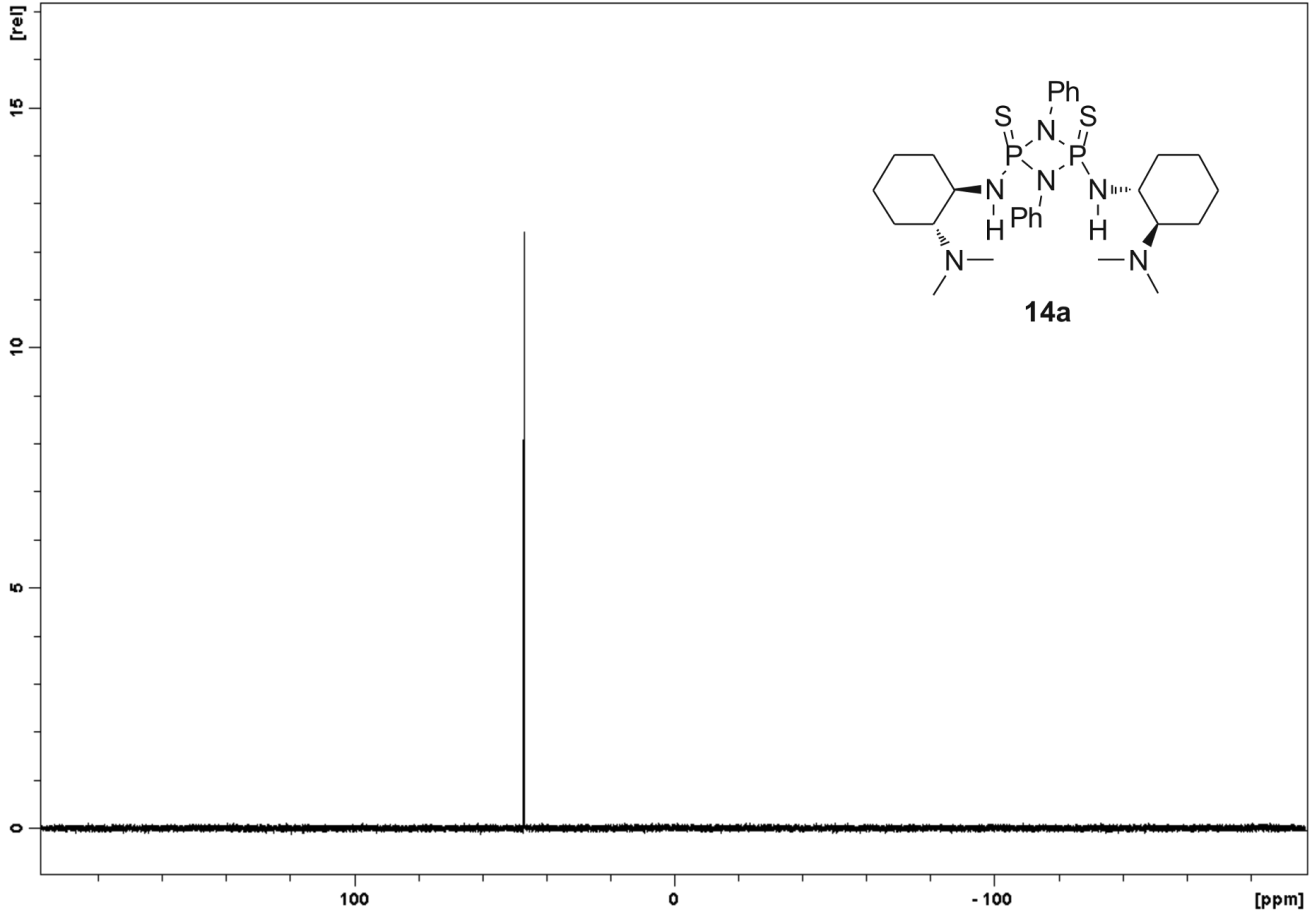
^31^P{1H} NMR spectrum in CDCl_3_ at rt showing *C*_2_ symmetry of **14a** at rt.

Since generating and separating mixtures of *cis*/*trans*-isomers is undesirable we attempted a *cis*-selective synthesis of bis(amido)cyclodiphosphazanes, which also allows a more modular approach to the design of catalysts. The generation of cyclic *cis*-di(P^III^)phosphazanes from bulky aliphatic amines is well known [38]. In most cases the dichloro derivatives can be obtained in a *cis-*selective fashion, as the *cis*-isomers are generally thermodynamically favored even with large R-groups on the exo-nitrogen substituents [39–41]. The situation is less clear for N(ring)-aryl-cyclodiphosphazanes, in which, dependent on the substitution pattern either *cis* or *trans*-isomers are favored [42]. We thus employed known aliphatic *cis*-[ClP(μ-N*t*-Bu)]_2_ **13** (Scheme 3) as starting compound, although a slightly decreased acidity of NH-protons can be expected because of the electron-donating effect of the *tert*-butyl moiety. Indeed when comparing the ^1^H NMR spectra of catalysts **14a** and **15**, an upfield shift of NH-protons for **15** is evident (Δδ = 0.4 ppm). However catalysts **15** and **16** could be obtained exclusively as *cis*-isomers after oxidation of the substituted cyclodiphosphazanes with sulfur (route **B**, **C**, Scheme 3). Furthermore the phenyl-substituted *cis* and *trans*-isomers **14a**/**14b** need to be stored under inert atmosphere because they decompose slowly when exposed to moisture, while the aliphatic cyclodiphosphazanes **15**/**16** are both completely stable to air. Cyclodiphosphazanes **14a**/**14b**/**15** are *C*_2_-symmetric on the NMR timescale at rt (**14a**, Figure 6). This is apparent from the ^31^P{H} NMR, which reveals the two phosphorus atoms to be magnetically equivalent. The same applies to the respective ^13^C and ^1^H NMR spectra (Supporting Information File 1).

**Figure 7:**
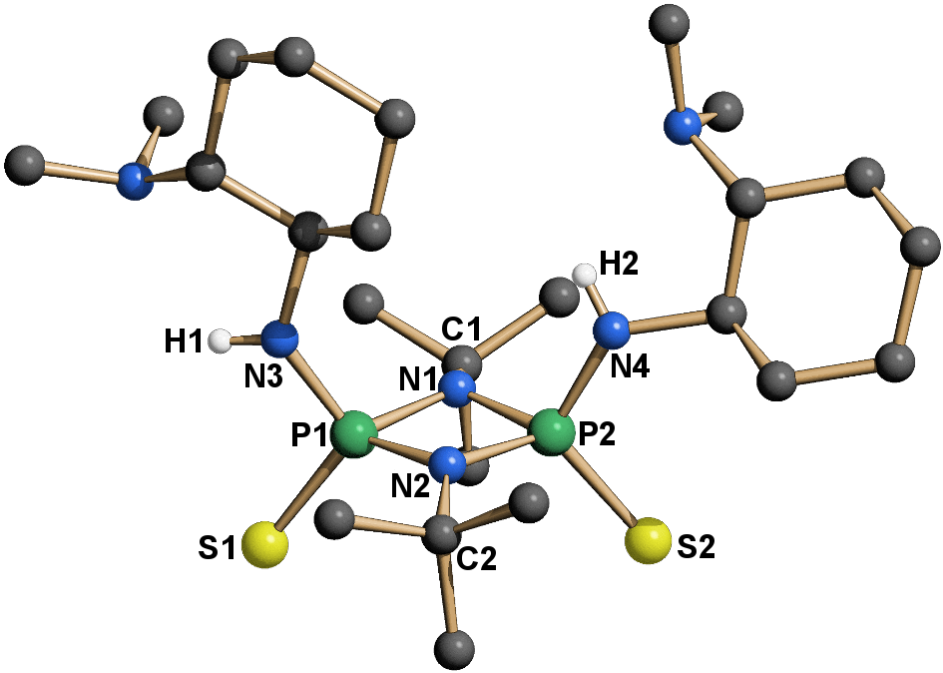
X-ray structure of **15**. The hydrogen atoms are omitted for clarity, except at nitrogen.

**Figure 8:**
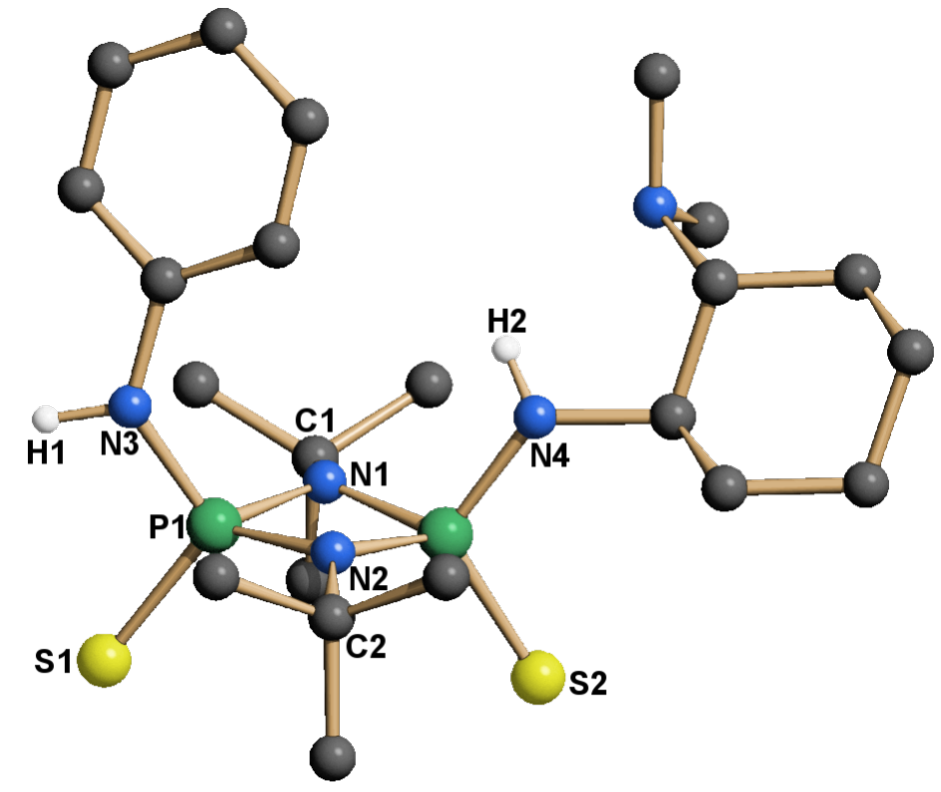
X-ray structure of **16**. The hydrogen atoms are omitted for clarity, except at nitrogen.

In the crystal structure (**14a**, **15**, **16**, Figure 5, Figure 7 and Figure 8) all structures adopt an (*endo*,*exo*) conformation. The cylcodiphosphazane ring in **14a** is quasi planar, with the sum of angles around N1 being 359.9° and the dihedral angle between the two [PNN] planes being 0.2°. The *tert*-butyl-substituted catalysts are somewhat more puckered, their sum of angles around N1 for **15**/**16** being 356.3° and 356.1° respectively, while the cyclodiphospazane ring is slightly distorted (angle between [PNN] planes 6.3° and 6.5°, Figure 7 and Figure 8). The angular sum around N3/N4 (**15**: 354.8°/353.6°, **16**: 356.9°/349.2°) is ambiguous with respect to the hybridization of nitrogen (nominal sp^3^/sp^2^: 328° and 360°, respectively) but suggests a hybridization with higher s-character and a delocalization of the lone pair onto the phosphorus atom. This is also supported by the shortened exocyclic P–N bonds and a downfield shift of the hydrogen atoms attached to nitrogen (**14a**, **15**, **16** δ-NH = 4.93, 4.50, 5.34/4.2 in CDCl_3_).

### Asymmetric Michael addition of β-nitrostyrene to 2-hydroxy-1,4-naphthoquinone

With these novel catalysts in hands we tested their efficiency in enantioselective catalysis. The reaction of 2-hydroxy-1,4-naphthoquinone (**17**) with β-nitrostyrene (**18**) is well-suited for comparison because it has already been studied while employing thiourea, squaramide and phosphor-diamide-based catalysts [43–45]. The BINOL-based catalysts **1** and **2** (Scheme 1) without Brønsted base functional group are both ineffective and only traces of the product could be isolated even after prolonged reaction times (Table 2). In contrast, catalyst **4**, derived directly from quinine, gives modest yields (60%) albeit with low enantiomeric excess (13% ee). Lowering the reaction temperature with catalyst **4** results in a slightly higher enantiomeric excess (27% ee, Table 2), however the yield drops to only 30%. We anticipated an increase in enantioselectivity for the epimer **5** as has been reported for 9-*epi*-aminoquinine-based thiourea catalysts in other organocatalytic reactions [46]. Indeed the yields are slightly higher (70%), however the enantioselectivity is low (11% ee). Altering the ligand backbone to the corresponding 9-*epi*-amino derivatives dramatically improves the efficiency of the catalysts. Catalysts **6** and **7a** afford significantly higher yields (90%, 92%) and also higher enantiomeric excesses (30%, 32%) in DCM and outperform quinine (77%, 22% ee). The 9-*epi*-aminocinchonidine derivative **7a** performs slightly better than the 9-*epi*-aminoquinidine-based catalyst. Changing the solvent to benzene again significantly improves the performance of catalyst **7a** with nearly quantitative yield and modest enantioselectivity (98%, 51% ee, Table 2).

**Table 2:** Evaluation of open-chain phosphorus-triamide catalysts **1**–**7a** (cf. Scheme 1).

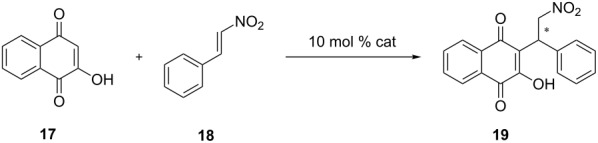					

catalyst	solvent	time	*T* (°C)	% yield^a^	% ee^b^

**1**	CH_2_Cl_2_	3 d	rt	< 5	n.d.
**2**	CH_2_Cl_2_	3 d	rt	< 5	n.d.
**1**^c^	CH_2_Cl_2_	3 d	rt	50	rac
**4**	CH_2_Cl_2_	14 h	rt	60	13 (*S*)
**4**	CH_2_Cl_2_	24 h	0	30	27 (*S*)
**5**	CH_2_Cl_2_	14 h	rt	70	11 (*R*)
quinine	CH_2_Cl_2_	14 h	rt	77	22 (*S*)
**6**	CH_2_Cl_2_	3 h	rt	90	30 (*R*)
**7a**	CH_2_Cl_2_	3 h	rt	92	32 (*R*)
**7a**	C_6_H_6_	3 h	rt	98	51(*R*)
**7a**	PhMe	3 d	-20	43	49 (*R*)
**7a**^d^	**C_6_H_6_**	**1 h**	**rt**	**98**	**51 (*R*)**

^a^Isolated yields. ^b^Chiral HPLC Daicel OJ, hexane/iPrOH 50/50, 1.0 mL/min, 254 nm, (*S*)-isomer: 12.3 min, (*R*)-isomer: 26.0 min. ^c^With 10 mol % Et_3_N. ^d^With 2% catalyst loading.

Introducing substituents to the aniline moieties generally had a negative impact on yields and enantioselectivities. The incorporation of strongly electron-withdrawing groups such as NO_2_ in *para*-position (**7e**) results in lower yields (69%, Table 3), probably because of the low solubility of the very polar catalysts, and also decreases the selectivity (43%, Table 3). Catalyst **7b** with CF_3_-groups in *meta*-position furnished comparable yields but a lower enantioselectivity. Chlorine and fluorine in *meta-*position also lead to diminished yields and lower enantioselectivities (Table 3). Concerning the substitution pattern a clear trend that correlates the increasing electron-withdrawing properties of substituents on the phenyl ring with the decreasing yields could be observed. This is well supported by the positive mesomeric effect of the methoxy-group in catalyst **7f**: While the selectivity decreases, substitution does not have an effect on yields (98% yield, 37% ee). Overall substitution on the phenyl moieties has a negative effect on the selectivity and also on the efficacy with electron-withdrawing groups.

**Table 3:** Influence of phenyl substituents on yields^a^ and selectivity (catalysts **7a**–**f**, cf. Scheme 1).

catalyst	R	% yield^a,b^	% ee^c^

**7a**	Ph	98	51 (*R*)
**7b**	3,5-(CF_3_)_2_-Ph	95	42 (*R*)
**7c**	3,5-Cl_2_-Ph	86	34 (*R*)
**7d**	3,5-F_2_	82	38 (*R*)
**7e**	4-NO_2_-Ph	69	43 (*R*)
**7f**	4-OMe-Ph	98	37 (*R*)

^a^All reactions carried out in benzene, 10 mol % cat., rt, 1 h. ^b^Isolated yields. ^c^Chiral HPLC Daicel OJ, hexane/iPrOH 50:50, 1.0 mL/min, 254 nm, (*S*)-enantiomer: 12.3 min (*R*)-enantiomer: 26.0 min.

While the flexible open-chain triamide catalysts (**6**, **7a**) proved to be strong promoters of the addition reaction with excellent yields, we assumed that the rigid cyclodiphosphazanes **14a**/**15**/**16** would be more selective catalyst motifs. Indeed *cis-*catalyst **14a** exhibited an excellent efficacy with 98% isolated yield and a good enantioselectivity of up to 75% ee (Table 4). It is noteworthy, that the enantiomeric excess can easily be increased to >99% by recrystallization. While enantioselectivities with **14a** are slightly higher in THF, the reaction proceeded faster in CH_2_Cl_2_ as indicated by TLC and was completed after 1 h. Employing the isomeric *trans*-catalyst **14b** resulted in much lower yields and poor enantioselectivitity (66%, 29% ee, Table 4). This strongly indicates that for an efficient and selective reaction both NH protons must participate in the activation of the substrate. The efficiency of catalyst **15** with very bulky *t*-Bu groups proved to be low (69% yield, Table 4), and surprisingly the resulting product was entirely racemic. It is probable, that the steric bulk of the *t*-Bu groups together with the *N*^1^,*N*^1^-dimethylcyclohexane-1,2-diamine moieties inhibits a specific binding of the catalyst to the substrate. Exchanging one of the *N*^1^,*N*^1^-dimethylaminocyclohexylamino groups for an aniline moiety, as in catalyst **16**, supports this. This sterically less demanding catalyst produced yields and selectivities (95% yield, 67% ee, Table 4) close to those of **14a**. While both structural motifs are efficient promoters of the reaction, open-chain phosphorus amides **1**–**7f** are outperformed by the more selective cyclodiphosphazanes **14a**/**16**, which can be attributed to the greater structural rigidity of the latter combined with a stronger hydrogen bonding.

**Table 4:** Evaluation of cyclodiphosphazane-based triamide catalysts **14a/b**–**16** (cf. Scheme 3).

catalyst	solvent	time	% yield^a^	% ee^b^

**14a**	CH_2_Cl_2_	1 h	98	74 (*S*)
**14b**	CH_2_Cl_2_	3 h	66	29 (*S*)
**14a**	**THF**	**3 h**	**98**	**75 (*S*)**
**15**	CH_2_Cl_2_	3 h	69	rac
**16**	CH_2_Cl_2_	3 h	95	67 (*S*)

^a^Isolated yields. ^b^Chiral HPLC Daicel OJ, hexane/iPrOH 50:50, 1.0 mL/min, 254 nm, (*S*)-enantiomer 12.3 min, (*R*)-enantiomer: 26.0 min.

### Mechanistic considerations

**Figure 9:**
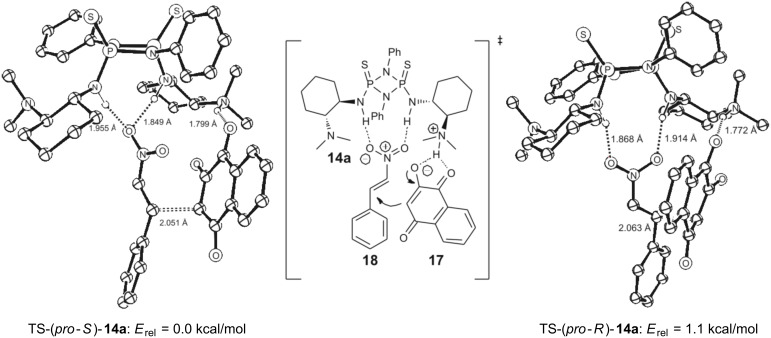
Enantiodetermining transition states TS-**14a**/TS-**14b** arising from the addition of 2-hydroxynapthoquinone to β-nitrostyrene.

We anticipated cyclodiphosphazane catalyst **14a** to act as a bidentate bifunctional H-bond-donor catalyst. Therefore a conformation of the catalyst similar to the crystal structure but with an (*exo*/*exo*)-conformation is reasonable as otherwise a bidentate H-bonding mechanism, as suggested by the experimental results, is not possible. In our model reaction **14a** activates the nitroolefin in the nucleophilic addition step as shown in Figure 9, the tertiary amine functionality meanwhile serves as a base that deprotonates 2-hydroxy-1,4-naphtoquinone (**17**, Figure 9). The resulting 1,4-dioxo-1,4-dihydronaphthalen-2-olate in turn acts as the nucleophile. In the transition state both, 1,4-dioxo-1,4-dihydronaphthalen-2-olate and β-nitrostyrene (**18**), are coordinated by hydrogen bonding to the quarternary amino group of the catalyst and the diamide structure of the cyclodiphosphazane, respectively. This is similar to the mechanism proposed by Takemoto [9] for the addition of diethylmalonate to β-nitrostyrene. As 2-hydroxynaphthoquinone is not symmetric, two stereocenters are created in the addition step, which gives rise to four different isomers. As with the foregoing calculation of binding energies (Table 1) we considered different conformational binding patterns of the nitroolefin to the diamide moiety with either any one or both oxygen atoms participating in hydrogen bonding. This results in potentially twelve different transition states, some of which present the same stationary point on the potential energy surface resulting in **9** (see Supporting Information File 1 for all TS) competing transition states. Relative energies in Figure 9 suggest that TS-(*pro-S*)-**14a** is the most favored by 1.1 kcal/mol compared to the lowest competing TS in *pro-R*-configuration TS-(*pro-R*)-**14a**. Thus the *S*-enantiomer of **19** should be generated primarily. The experimentally favored enantiomer is indeed (*S*)-configured with 75% ee. The computationally found difference in energies is not high enough to wholly explain the experimentally found selectivity. This can be tentatively attributed to the lower accuracy of the employed SVP-basis set in describing H-bonded systems.

## Conclusion

A series of new phosphorus (tri)amides, which includes the first chiral P^V^-cyclodiphosphazane, is synthesized and is successfully employed as HB organocatalysts in the Michael addition reaction of 2-hydroxynaphthoquinone to β-nitrostyrene. The open-chain triamide catalyst **7a** performs with nearly quantitative yields (98%) and moderate enantiomeric excess of 51%, even at 2% catalyst loading (Table 2). Cyclodiphosphazane *cis*-**14a** shows the same efficiency with 98% isolated yields and an even improved enantiomeric excess of up to 75% (Table 4). Notably, its isomer *trans*-**14b** performs markedly inferior compared to *cis-***14a**. This is attributed to the disability of *trans*-**14b** to participate in bidentate hydrogen bonding. The high efficiency of *cis-***14a** with respect to the yields can be attributed to the relative strength of its hydrogen bonds: Computations of the hydrogen-bond-donor strength reveal a higher bonding energy to nitrobenzene (7.2 kcal/mol, Table 1) than for the comparable thiourea motif (6.5 kcal/mol). In terms of yields (Tables 2–4), the new phosphorusamide catalysts are competitive with standard HB donors such as (thio)ureas in the tested Michael addition. A fine tuning of the structural motifs might improve enantioselectivity even further. Especially the rigid and sterically crowded P^V^-cyclodiphosphazane structures will have a high potential for future catalyst designs not only in organocatalysis but also in applications such as anion recognition.

## Experimental

All reactions were conducted under argon atmosphere on a dual manifold Schlenk line and in oven-dried glassware, unless otherwise stated. All solvents were dried according to known methods and distilled prior to use. NMR spectra were recorded on Bruker Avance (300, 400, 500 and 600 MHz) instruments. High resolution mass spectra were conducted at the mass spectrometry facility of the Institute for Organic Chemistry of the University of Cologne. Enantiomeric excess was determined by chiral HPLC. We employed the LaChrom elite unit by Hitachi together with the chiral column Chiralcel OJ-H in 25 cm length. The enantiomers of the product were determined by reference spectra. Crystal data, NMR spectra and coordinates of stationary points/transition states as well as experimental procedures for the preparation of catalysts **1**–**7f** can be found in Supporting Information File 1. Crystallographic data have been deposited with the Cambridge Crystallographic Data center. These data can be obtained free of charge from The Cambridge Crystallographic Data Centre via http://www.ccdc.cam.ac.uk/data_request/cif.

**Typical procedure for the Michael addition of 2-hydroxy-1,4-naphthoquinone to *trans*-β-nitrostyrene:** To a stirred mixture of 2-hydroxy-1,4-naphthoquinone (34.8 mg, 0.2 mmol) and *trans*-β-nitrostyrene (32.8 mg, 0.22 mmol) in dry DCM (1.2 mL) was added catalyst **14a** (11.8 mg, 0.02 mmol) and stirred for 3 h at room temperature. After completion of the reaction the solvent was removed in vacuo and the obtained residue was purified by column chromatography over silica (EtOAc/hexane 1:3) to afford the corresponding Michael adduct as a yellowish solid in 98% yield. The product is a known compound and its data are identical to those reported in literature hitherto [47].

**Preparation of *cis*-2,4-bis(((*R*,*R*)-2-(dimethylamino)cyclohexyl)amino)-1,3-diphenylcyclodiphosphazane-2,4-disulfide (14a):** To a stirred solution of (*R*,*R*)-*N*^1^,*N*^1^-dimethylcyclohexane-1,2-diamine (400 mg, 2.81 mmol) in DCM (4 mL) was added a solution of 2,4-dichloro-1,3-diphenylcyclodiphosphazane-2,4-disulfide (532 mg, 1.40 mmol) in DCM (2 mL) via syringe at 0 °C. After 0.5 h, Et_3_N (284 mg, 2.81 mmol) was added via syringe and the mixture was kept stirring at 0 °C for further 0.5 h. The reaction was allowed to warm to rt and stirred for 1 h at this temperature. The solvent was removed in vacuo and the yellowish residue was purified by column chromatography over neutral alumina (grade V, EtOAc/hexane 1:7, *R*_f_ (*cis*) 0.32) to yield 21% (171 mg) of *cis-***14a** as a white solid. Mp >110 °C dec; ^1^H NMR (300 MHz, CDCl_3_) δ 7.56 (d, *J* = 7.8 Hz, 4H), 7.30 (t, *J* = 7.8 Hz, 4H), 7.07 (t, *J* = 7.4 Hz, 2H), 4.93 (s, 2H, NH), 3.14 (s, 2H), 2.54 (s, 2H), 2.21–2.15 (m, 2H), 2.09 (s, 12H, CH_3_), 1.82–1.61 (m, 6H), 1.26–1.07 (m, 8H); ^13^C NMR (75 MHz, CDCl_3_) δ 136.3, 129.3, 123.8, 119.7, 68.1, 54.9, 40.5, 34.6, 25.3, 24.8, 21.5; ^31^P{^1^H} NMR (121 MHz, CDCl_3_) δ 46.80; FTIR (ATR) ν (cm^−1^): 3049 (s), 2933 (s), 2860 (m), 1635 (m), 1598 (s), 1496 (s), 1282 (m), 1132 (w), 1099 (m), 952 (m); HRMS–ESI^+^ (*m*/*z*): [M + H]^+^ calcd for C_28_H_44_N_6_P_2_S_2_ + H, 591.2616; found, 591.2610; X-ray crystal data: CCDC-958718 (**14a**) contains the supplementary crystallographic data for this compound.

**Preparation of *cis*-2,4-bis(((*R*,*R*)-2-(dimethylamino)cyclohexyl)amino)-1,3-di-*tert*-butylcyclodiphosphazane-2,4-disulfide (15):** A solution of (*R*,*R*)-*N*^1^,*N*^1^-dimethylcyclohexane-1,2-diamine (200 mg, 1.4 mmol) and Et_3_N (284 mg, 1.4 mmol) in Et_2_O (2 mL) was added dropwise to a solution of *cis*-(*t-*BuNPCl)_2_ (196 mg, 0.7 mmol) in Et_2_O (4 mL) at 0 °C. After stirring at this temperature for 1 h, the mixture was allowed to warm to room temperature and stirred for further 16 h. The resulting suspension was filtered under argon and the filtrate concentrated in vacuo. The residue was redissolved in toluene (5 mL), elemental sulfur was added (90 mg, 2.8 mmol) and stirred for 16 h at 50 °C. The solvent was removed in vacuo and the crude product was purified by column chromatography on silica (EtOAc/MeOH/NEt_3_ 80:20:1 *R*_f_ 0.15) yielding 54% (210 mg, 0.76 mmol) of **15** as a white solid. Mp 205 °C; ^1^H NMR (300 MHz, CDCl_3_) δ 4.50 (s, 2H, NH), 3.04 (s, 2H), 2.84–2.81 (m, 2H), 2.18 (s, 12H), 2.11 (t, *J* = 9.0 Hz, 2H), 1.86–1.73 (m, 4H), 1.63 (s, 2H), 1.58 (s, 18H), 1.28–1.11 (m, 8H); ^13^C NMR (75 MHz, CDCl_3_) δ 68.4 (t, *J*_PC_ = 5.9 Hz), 56.8, 55.1, 41.2, 33.8, 30.2 (t, *J*_PC_ = 4.6 Hz), 25.4, 24.6, 21.7; ^31^P{^1^H} NMR (121 MHz, CDCl_3_) δ 46.37; FTIR (ATR) ν (cm^−1^): 2985 (s), 1639 (s), 1531 (s), 1512 (m), 1400 (m), 1242 (w), 1002 (w); HRMS–ESI^+^ (*m*/*z*): [M + H] calcd for C_24_H_52_N_6_P_2_S_2_ + H, 551.3242; found, 551.3237; X-ray crystal data: CCDC-958719 (**15**) contains the supplementary crystallographic data for this compound.

**Preparation of *cis*-((*R*,*R*)-2-(dimethylamino)cyclohexyl)amino)-4-anilino-1,3-di-*tert*-butylcyclodiphosphazane-2,4-disulfide (16)**: A solution of aniline (130 mg, 1.4 mmol) and Et_3_N (141 mg, 1.4 mmol) in THF (4 mL) was added dropwise to a solution of *cis*-(*t*-BuNPCl)_2_ (385 mg, 1.4 mmol) in THF (10 mL) at −78 °C. After stirring at this temperature for 1 h, the mixture was allowed to warm to room temperature and stirred for further 16 h. To the suspension was then added a solution of (*R*,*R*)-(*N*^1^,*N*^1^-dimethylcyclohexane-1,2-diamine (199 mg, 1.4 mmol) and Et_3_N (141 mg, 1.4 mmol) in THF (2 mL) at −78 °C. After 0.5 h the mixture was allowed to warm to rt and stirred overnight. The resulting suspension was filtered under argon and the filtrate concentrated in vacuo. The residue was redissolved in toluene (10 mL), elemental sulfur was added (96 mg, 3 mmol) and stirred for 16 h at 50 °C. The solvent was removed in vacuo and the crude product was purified by column chromatography on silica (gradient EtOAc/hexane 1:1 to EtOAc) yielding 26% (183 mg, 0.36 mmol) of 16 as a white solid. Mp 191 °C; ^1^H NMR (300 MHz, CDCl_3_) δ 7.26 (t, *J* = 7.8 Hz, 2H), 7.15 (d, *J* = 8.2 Hz, 2H), 7.03 (t, *J* = 7.3 Hz, 1H), 5.35 (d, *J*_PH_ = 13.6 Hz, 1H, NH), 4.24 (s, 1H, NH)), 3.19–3.10 (m, 1H), 2.87–2.83 (m, 1H), 2.10 (m, 1H), 2.08 (s, 6H), 1.72 (m, 2H), 1.56–1.50 (m, 10H), 1.45 (s, 9H), 1.23–1.02 (m, 4H); ^13^C NMR (75 MHz, CDCl_3_) δ 140.0 (d, *J*_PC_ = 7.0 Hz), 129.7, 123.8, 120.5 (d, *J*_PC_ = 5.5 Hz), 68.5 (d, *J*_PC_ = 11.3 Hz), 57.5, 56.9, 55.3, 41.5, 35.0, 29.9, 29.8, 25.4, 24.6, 21.8; ^31^P{^1^H} NMR (121 MHz, CDCl_3_) δ 47.74 (d, *J*_PP_ = 35.8 Hz), 38.83 (d, *J*_PP_ = 35.8 Hz); FTIR (ATR) ν (cm^−1^): 3248 (s), 2974 (m), 2937 (m) , 2868 (w), 1598 (w), 1494 (m), 1386 (m), 1369 (m), 1055 (s), 902 (s); HRMS–ESI (*m*/*z*): [M + H]^+^ calcd for C_22_H_41_N_5_P_2_S_2_ + H, 502.2351; found, 502.2344; X-ray crystal data: CCDC-958720 (16) contains the supplementary crystallographic data for this compound.

## Computational details

All theoretical calculations were performed with the program package TURBOMOLE-6.3 [48]. The employed density functionals was the nonempirical TPSS-functional developed by Tao, Perdew, Scuseria and Staroverov [49], combined with the contracted def-SVP and the def2-TZVP basis set by Ahlrich et al. [50–51] as specified. The multipole accelerated resolution of identity approximation for two electron integral evaluation was used. All stationary points were fully optimized and confirmed by separate analytical frequency calculations. Transition structures were optimized with quasi-Newton–Raphson methods by using the Powell update algorithm for hessian matrix approximation (subsequent analytical frequency calculation). Calculating hydrogen-bonding interactions, density functional theory (DFT) is frequently employed. The B3LYP functional is common for this purpose, however we chose the non-empirical TPSS-functional as previous studies suggest a greater accuracy for dissociation energies and geometries of weakly bonded systems [52].

## Supporting Information

File 1 Detailed experimental procedures for all compounds and precursors, copies of ^13^C/^1^H NMR spectra for all compounds, DOSY, computational coordinates, X-ray-data.
