# Supplementary material for: New hydrogen-bonding organocatalysts: Chiral cyclophosphazanes and phosphorus amides as catalysts for asymmetric Michael additions
Source: Beilstein J Org Chem. 2014 Jan 21;10:224–36. doi: 10.3762/bjoc.10.18 (PMC3944119; doi:10.3762/bjoc.10.18)
Supplement: File 1 — Detailed experimental procedures for all compounds and precursors, copies of 13C/1H NMR spectra for all compounds, DOSY, computational coordinates, X-ray-data. [file Beilstein_J_Org_Chem-10-224-s001.pdf]

Supporting Information

for

New hydrogen-bonding organocatalysts: Chiral cyclophosphazanes and phosphorus amides as catalysts for asymmetric Michael additions

Helge Klare, Jörg M. Neudörfl[§] and Bernd Goldfuss*

Address: Department of Chemistry, Universität zu Köln, Greinstrasse 4, D-50939 Köln, Germany,

Fax: +49(0)221-470-5057

Email: Bernd Goldfuss* - goldfuss@uni-koeln.de

* Corresponding author

[§]X-Ray analysis

Detailed experimental procedures for all compounds and precursors, copies of $^{13}C/^{1}H$ NMR spectra for all compounds, DOSY, computational coordinates, X-ray data

Experimental ... S3

N,*N'*-Diphenylphosphorodiamido chloridate ... S3

Catalyst **1** - (*S*)-2'-(*N*,*N'*-diphenylphosphorodiamido-oxy)-1,1'-binaphthyl-2-ol ... S4

2'-(Methoxymethoxy)-1,1'-binaphthyl-2-ol ... S5

(*S*)-2-Methoxy-2'-(*N*,*N'*-di-(3,5-bis(trifluormethyl))phosphorodiamido-oxy)-1,1'-binaphthyl ... S5

Catalyst **2** - (*S*)-2'-(*N*,*N'*-di-(3,5-bis(trifluormethyl))phosphorodiamido-oxy)-1,1'-binaphthyl-2-ol ... S6

Catalyst **4** - (*N*,*N'*-diphenylphosphorodiamido-oxy)-quinine ... S7

Catalyst **5** - (*N*,*N'*-diphenylphosphorodiamido-oxy)-9-*epi*-quinine ... S8

Catalyst **6** - (*N*,*N'*-diphenylphosphorodiamido)-9-amido-*epi*-quinine ... S9

Catalyst **7a** - (*N*,*N'*-diphenylphosphorodiamido)-9-amido-*epi*-cinchonidine ... S10

Catalyst **7b** - (*N*,*N'*- (3,5-bis(trifluoromethyl)phenylphosphorodiamido)-9-amido-*epi*-cinchonidine ... S11

Catalyst **7c** - (*N*,*N'*- (3,5-dichloro-phenyl)phosphorodiamido)-9-amido-*epi*-cinchonidine ... S12

Catalyst **7d** - (*N*,*N'*-(3,5-fluoro-phenyl)phosphorodiamido)-9-amido-*epi*-cinchonidine ... S13

Catalyst **7e** - (*N*,*N'*-(4-nitro-phenyl)phosphorodiamido)-9-amido-*epi*-cinchonidine ... S14

Catalyst **7f** - (*N*,*N'*-(4-methoxy-phenyl)phosphorodiamido)-9-amido-*epi*-cinchonidine ... S15

2,2,2,4,4,4-Hexachloro-1,3-diphenyl-1,3,2,4-diazaphosphetidine ... S16

12 - 2,4-Dichloro-1,3-diphenyl-1,3,2,4-diazaphosphetidin-2,4-disulfide ... S16

Catalyst **14a**/**b** - *cis/trans*-2,4-bis(((1*R*,2*R*)-2-(dimethylamino)cyclohexyl)amino)-1,3-diphenyl-1,3,2,4-diazadiphosphetidine-2,4-disulfide ... S17

1,3-di-*tert*-Butyl-2,4-dichloro-1,3,2,4-diazadiphosphetidine ... S17

Catalyst **15** - *cis*-1,3-di-*tert*-butyl-2,4-bis(((1*R*,2*R*)-2-(dimethylamino)cyclohexyl)amino)-1,3,2,4-diazadiphosphetidine-2,4-disulfide ... S18

Catalyst **16** - 1,3-di-*tert*-butyl-2-(((1*R*,2*R*)-2-(dimethylamino)cyclohexyl)amino)-4-(phenylamino)-1,3,2,4-diazadiphosphetidine-2,4-disulfide ... S19

DOSY and variable temperature NMR ... S20

NMR spectra ... S21

Computed structures ... S40

Stationary points of compounds shown in Scheme 2 (TPSS/def2-TZVP) ... S40

Transition states (TPSS/def-SVP) ... S46

Pro-*R* ... S46

Pro-*S* ... S57

Crystal data ... S67

Catalyst **6** ... S67

Catalyst **7a** ... S73

Catalyst **14a** ... S79

Catalyst **15** ... S83

Catalyst **16** ... S86

References ... S92

General: All reactions were conducted under argon-atmosphere on a dual manifold Schlenk-line unless otherwise mentioned and in oven-dried glass-ware. All solvents were dried according to known methods and distilled prior to use. Starting materials 9-*epi*-quinine, 9-*epi*-cinchonidine, 9-amino-(9-deoxy)-*epi*-quinidine and 9-amino-(9-deoxy)-*epi*-cinchonidine were synthesized according to literature procedures[1,2]. Other reagents were commercially available and used as purchased.

Experimental

N,N'-diphenylphosphorodiamido chloridate

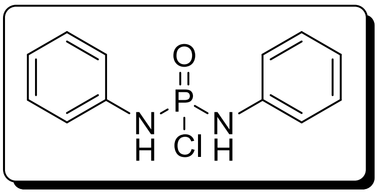

In a 50 mL Schlenk-tube 1.22 mL $POCl_3$ (2.05 g, 13.4 mmol) were dissolved in 14 mL THF. To the ice-cooled solution were added 4.9 mL (5 g, 53.7 mmol) of freshly distilled aniline in 14 mL THF via a dropping funnel under vigorous stirring until the addition was finished after 1 hour. The mixture was then left to stand at 4 °C over night. The solids were separated from the solvent by filtration and washed with cold water (3x 10 mL) to remove anilinium hydrochloride. The product was then recrystallized from EtOH.

Yield: 1.8 g = 50 %, **m.p**. 179 °C, **^{1}H-NMR** (300 Mhz, Acetone-D_6): δ 8.18 (d, J = 10.7 Hz), 7.24-7.32 (m, 4H), 6.98-7.04 (m, 1H) **^{13}C-NMR** (75 Mhz, Acetone-D_6): δ 140.1 (d, $^{2}J(P\text{-}C)$ = 7.2 Hz), 129.6, 123.0, 119.4 (d, $^{3}J(P\text{-}C)$ = 6.5 Hz) **^{31}P-NMR** (127 Mhz, Acetone-D_6): δ -15.3

Catalyst 1 - (*S*)-2'-(*N,N'*-diphenylphosphorodiamido-oxy)-1,1'-binaphthyl-2-ol

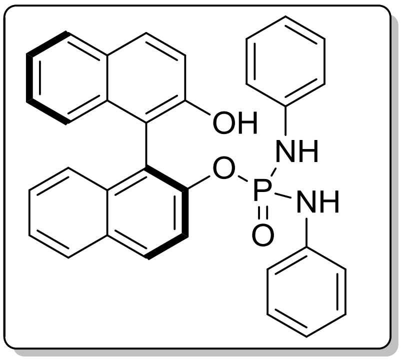

In a sealed Schlenk-tube *S*-(-)-BINOL (200 mg, 0.7 mmol) was suspended in 2 mL toluene. After stirring for 10 minutes the toluene was removed in vacuo. The remaining *S*-(-)-BINOL was then dissolved in 6 mL THF and cooled to 0 °C. Then 2 eq. 1.6M n-BuLi in hexane (1.4 mmol, 0.88 mL) were added over a period of 5 minutes. The mixture was subsequently stirred at 0 °C for a further 10 minutes and then at 20 °C for further 15 minutes. After cooling to 0 °C, 1 eq. of *N*,*N'*-diphenylphosphorodiamidochloridate (186 mg, 0.7 mmol) in 4 mL THF was added slowly via syringe and the mixture was stirred for 30 minutes. After removing the ice-bath, stirring was held up for an additional 2 h. The reaction was then quenched by addition of water (10 mL). The solution was extracted with Et_2O (3x 5 mL) and CH_2Cl_2 (3x 5 mL). The combined org. phases were dried over Na_2SO_4 and concentrated. The concentrated organic phase was purified by column chromatography on silica gel (cHex/MTBE/Acetone 5:1:1) and then recrystallized from Et_2O to afford the compound as colourless crystals.

Yield: 230 mg = 64 %; **m.p**. 105 °C; **^{1}H-NMR** (300 Mhz, $CDCl_3$): δ 7.81-7.89 (m, 4H), 7.67 (d, *J* = 9.0 Hz, 1H), 7.44 (t, *J* = 7.4 Hz, 1H), 7.20-7.36 (m, 5H), 6.73-7.12 (m, 9H), 6.60 (d, *J* = 7.8 Hz, 2H), 6.52 (d, *J* = 7.7 Hz, 2H), 5.82 (d, *J* = 9.1 Hz, 1H, NH), 5.36 (d, *J* = 9.5 Hz, 1H, NH); **^{13}C-NMR** (75 Mhz, $CDCl_3$): δ = 152.4, 147.7, 147.6, 138.7, 138.4, 133.9, 131.8, 131.0, 130.7, 129.5, 129.3, 129.1, 128.4, 127.5, 127.2, 126.1, 126.0, 124.8, 124.0, 122.8, 122.5, 121.2, 119.8, 118.8, 118.7, 118.6, 118.5, 116.1; **^{31}P-NMR** (127 Mhz, $CDCl_3$): δ = -0.9 (t (b)); **FT-IR (ATR)**: ν [cm^{-1}] : 3417 (s), 2089 (w), 1628 (s), 1601 (m), 1497 (m), 1396 (w), 1283 (w), 1207 (m), 972 (w), 816 (w); **HRMS** (ESI^+): calcd. for [$C_{32}H_{25}N_2O_3P$ + H] 517.1675; found: 517.1678

(S)-2'-(methoxymethoxy)-1,1'-binaphthyl-2-ol

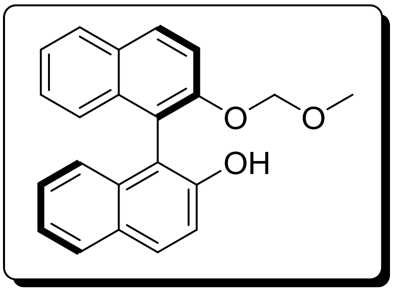

Following a procedure of Itoh et al.[3] 4.29 g *S*-(-)-BINOL were dissolved in 30 mL DCM and 5.74 mL (33.5 mmol) diisopropylethlamine (DIPEA) were added at 0 °C and the mixture was stirred for 2.5 h. Subsequently 1.7 mL (22.5 mmol) chloromethylmethylether (MOMCl) were added at 0 °C and the mixture stirred for 15 min. The reaction was quenched by addition of 20 mL diluted HCl at 0 °C and then extracted with DCM (3x 20 mL). The combined organic layers were dried over Na_2SO_4 and concentrated. The product was purified by silica gel flash-column chromatography (hexane/acetone 10/1) yielding (S)-2-hydroxy-2'-(methoxymethyl)oxy-1,1'-binaphthyl as a white solid with diprotected (*S*)-BINOL (0.95 g, 18%) as a by-product.

Yield: 2.9 g, 62 %; ^{1}**H-NMR** (300 Mhz, $CDCl_3$): 8.02 (d, *J* = 9.1 Hz, 1H), 7.90 (d, *J* = 8.7 Hz, 2H), 7.85 (d, *J* = 8.2 Hz, 1H), 7.59 (d, *J* = 9.1 Hz, 1H), 7.18-742 (m, 6H), 7.07 (d, *J* = 8.3 Hz, 1H), 5.10 (d, *J* = 6.9 Hz, 1H), 5.05 (d, *J* = 6.9 Hz, 1H), 4.98 (s, 1H), 3.17 (s, 3H)

(*S*)-2-methoxy-2'-(N,N'-di-(3,5-bis(trifluormethyl))phosphorodiamido-oxy)-1,1'-binaphthyl

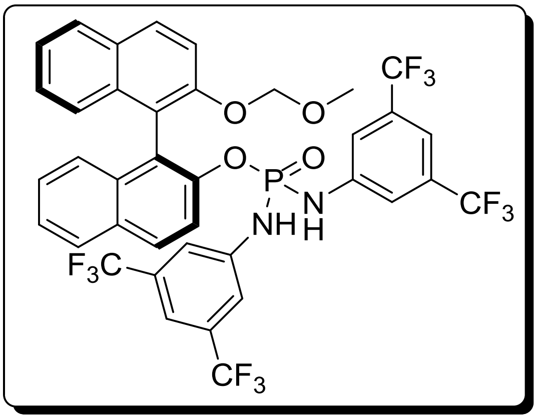

To a solution of 300 mg (0.91mmol) (S)-2-hydroxy-2'-(methoxymethyl)oxy-1,1'-binaphthyl in 2 mL Et_2O was added 1 eq. n-BuLi (0.57 mL, 0.91 mmol, 1.6M in hexanes) via syringe. After removal of cooling the mixture was stirred at room temperature for 2 h. The suspension was cooled to -78 °C and 1.2 eq. of $POCl_3$ in 2 mL Et_2O were added slowly via syringe. The mixture was allowed to warm up to rt over a period of 1.5 h. After stirring for an additional 0.5 h at rt, the solvent and traces of unreacted $POCl_3$ were removed in vacuo and the resulting white solid was redissolved in dry pyridine and cooled to 0 °C. To this mixture 0.28 mL (1.82 mmol) of *m*-$(CF_3)_2$-aniline were added and stirred over night. After removal of solvent in vacuo 5 mL of water were added. The mixture was extracted with DCM (3x 15 mL) and the combined organic-layers dried over Na_2SO_4 and concentrated. The product was purified by silica gel flash-column chromatography (c-hexane / MtBE 5 /1).

Yield: 410 mg = 54 %; **m.p.** 72 °C; ^{1}**H-NMR** (300 Mhz, $CDCl_3$): 7.90-7.95 (m, 2H), 7.79 (d, *J* = 8.2 Hz, 2H), 7.74 (d, *J* = 8.2 Hz, 1H), 7.37-7.47 (m, 3H), 7.24-7.32 (m, 7H), 7.03-7.14 (m, 3H), 6.83 (d, *J* = 8.4 Hz, 1H, NH), 6.75 (s, 2H), 5.91 (d, *J* = 6.4 Hz, 1H), 4.94-4.99 (m, 2H), 2.91 (s, 3H)

Catalyst 2 - (*S*)-2'-(N,N'-di-(3,5-bis(trifluormethyl))phosphorodiamido-oxy)-1,1'-binaphthyl-2-ol

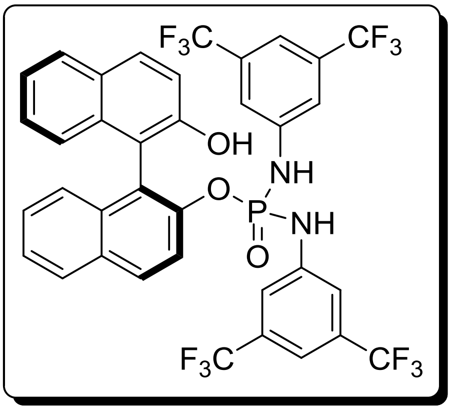

To 200 mg (0.24 mmol) (*S*)-2-methoxy-2'-(N,N'-di-(3,5-bis(trifluormethyl))phosphorodiamido-oxy)-1,1'-binaphthyl in a 25 mL flask 6 eq. of trifluoracetic acid (2.9 mL, 0.5 M in DCM) were added under constant stirring at 25 °C. After TLC indicated completion of the reaction (3 h) all solvent and TFA were removed in vacuo. The remaining solid was purified by silica gel flash-column chromatography (n-hexane / acetone 10/1) yielding **2** as a white solid.

Yield: 140 mg = 74 %; **m.p**. 103 °C; **^{1}H-NMR** (300 Mhz, $CDCl_3$): δ 7.95 (t, *J* = 8.8 Hz, 2H), 7.60-7.67 (m, 3H), 7.50 (t, *J* = 7.5 Hz, 1H), 6.94-7.33 (m, 12H), 6.65 (d, *J* = 8.5 Hz, 1H, NH), 6.07-6.17 (m, 2H, NH / OH), 6.52 (d, *J* = 7.7 Hz, 2H), 5.82 (d, *J* = 9.1 Hz, 1H, NH), 5.36 (d, *J* = 9.5 Hz, 1H, NH); **^{13}C-NMR** (100 Mhz, $CDCl_3$): δ 151.9, 147.0, 146.9, 139.8 (d, $J_{P\text{-}C}$ = 1.8 Hz), 139.6 (d, $J_{P\text{-}C}$ = 1.9 Hz), 133.7 (d, $J_{P\text{-}C}$ = 1.3 Hz), 133.3, 132.7, 132.2 (d, $J_{P\text{-}C}$ = 1.1 Hz), 131.5, 131.0, 129.1, 128.6, 128.4, 128.1, 127.6, 126.8, 126.0, 124.3, 123.0, 122.2, 121.4, 118.5, 118.4, 118.3, 118.1, 118.0, 116.5, 116.4, 114.1; **^{31}P-NMR** (162 Mhz, $CDCl_3$): δ -3.7; **^{19}F-NMR** (376 Mhz, $CDCl_3$): δ -63.17, -63.22; **FT-IR (ATR)**: ν [cm^{-1}] : 3424 (s), 1622 (s), 1506 (w), 1470 (w), 1382 (m), 1278 (m), 1182 (m), 1132 (m), 1005 (w), 982 (m); **HRMS** (ESI^+): calcd. for [$C_{36}H_{21}F_{12}N_2O_3P$ + H] 789.1170; found: 789.1175

Catalyst 4 - (*N,N'*-diphenylphosphorodiamido-oxy)-quinine

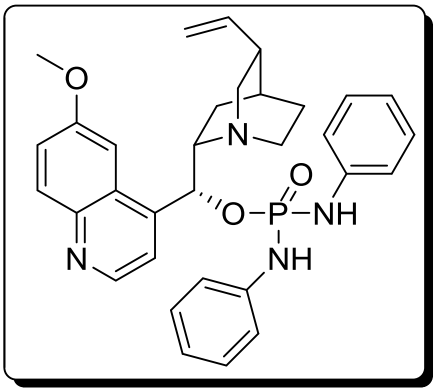

In a Schlenck-tube with magnetic stirring-bar 500 mg (1.54 mmol) quinine were suspended in 3 mL of toluene. The toluene was removed in vacuo and the residue was dissolved in 12 mL THF. After cooling to 0 °C 1 eq. n-BuLi (0.96 mL, 1.6 M in hexanes) were slowly added via syringe. The solution was then left to stir for 1.5 h at 0 °C. To the solution was then added 410 mg (1.54 mmol) *N,N'*-diphenylphosphorodiamido chloridate in 7 mL THF at 0 °C. Stirring was maintained at this temperature for 1 h and then allowed to warm up to rt over night. The reaction was then quenched with 10 mL water and extracted with 2x 10 mL Et_2O / DCM each. The combined organic extracts were dried over Na_2SO_4, evaporated and chromatographed on silica gel flash column (EtOAc / MeOH / conc. NH_3 = 75/5/1; R_f = 0.22) to give **4** as a white solid. (150 mg = 18 %).

Yield: 150 mg = 18 %; **m.p.** 79 °C; **^{1}H-NMR** (500 Mhz, DMSO-d_6): δ 8.54 (d, *J* = 4.4 Hz, 1H), 8.04 (d, *J* = 8.7 Hz, 1H), 7.87 (d, *J* = 9.2 Hz, 1H), 7.71 (d, *J* = 9.3 Hz, 1H), 7.54 – 7.26 (m, 4H), 7.19 – 7.13 (m, 4H), 6.85 – 6.62 (m, 6H), 5.89 (ddd, *J* = 17.5, 10.3, 7.4 Hz, 1H), 5.03 – 4.99 (m, 2H), 3.87 (s, CH_3), 3.03 – 2.91 (m, 1H), 2.82 – 2.70 (m, 1H), 2.45 – 2.27 (m, 2H), 2.24 – 2.13 (m, 1H), 2.04 – 1.88 (m, 1H), 1.79 – 1.53 (m, 3H), 1.49 – 1.36 (m, 1H); **^{13}C-APT** (101 MHz, $CDCl_3$) δ 158.3, 147.2, 144.4, 141.9, 139.3, 139.0, 134.0, 131.6, 131.6, 129.4, 129.0, 128.5, 127.0, 122.4, 122.2, 119.0, 118.5 (d, *J* = 7.0 Hz), 118.1 (b), 114.8, 100.6, 74.7, 60.3, 56.3, 55.9, 42.8, 39.6, 32.4, 27.4, 25.3; **^{31}P NMR (121 MHz, $CDCl_3$):** δ = 1.72.; **FT-IR (ATR)**: ν [cm^{-1}] : 3155 (m) 2943 (m), 1622(w) 1600 (m), 1498 (s), 1419 (w), 1286 (m), 1224 (s, P=O), 1031 (m), 1001 (s), 937 (m); **LRMS** (ESI^+): calcd. for [$C_{32}H_{35}N_4O_3P$ + H] 555; found: 555.18

Catalyst 5 - (*N,N'*-diphenylphosphorodiamido-oxy)-9-*epi*-quinine

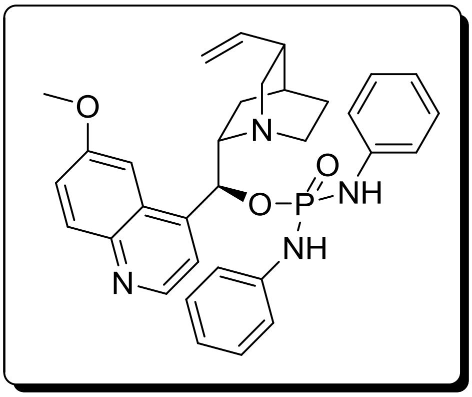

In a Schlenck-tube with magnetic stirring-bar 500 mg (1.54 mmol) 9-*epi*-quinine (9-*epi*-quinine synthesized in 2 steps with an overall yield of 68% following the procedure of Yang et al.[1]) were suspended in 3 mL of toluene. The toluene was removed in vacuo and the residue was dissolved in 12 mL THF. After cooling to 0 °C 1 eq. n-BuLi (0.96 mL, 1.6 M) was slowly added via syringe. The solution was then left to stir for 1.5 h at 0 °C. To the solution was then added 410 mg (1.54 mmol) *N,N'*-diphenylphosphorodiamido chloridate in 7 mL THF at 0 °C. Stirring was maintained at this temperature for 1 h and then allowed to warm up to rt over night. The reaction was then quenched with 10 mL water and extracted with 2x 10 mL Et_2O / DCM each. The combined organic extracts were dried over Na_2SO_4, evaporated and chromatographed on silica gel flash column (EtOAc / MeOH / conc. NH_3 = 75/5/1; R_f = 0.26) to give **5** as a white solid.

Note: For information on conformers read DOSY and variable temperature NMR p. 21.

Yield: 172 mg = 20 %; **m.p.** 116 °C; **^{1}H-NMR** (600 Mhz, $CDCl_3$) (*Major Conformer*): δ = 9.07 (s, 1H, NH), 8.69 (d, *J* = 4.5 Hz, 1H), 7.99 (d, *J* = 9.2 Hz, 1H), 7.62 (d, *J* = 2.3 Hz, 1H), 7.38-7.36 (m, 1H), 7.22-6.83 (m, 12H), 6.24 (t, *J* = 10.0 Hz, 1H), 5.85 (ddd, *J* = 17.4, 10.4, 7.3 Hz, 1H), 5.75 (d, *J* = 6.8 Hz, 1H, NH), 5.10-4.97 (m, 2H), 3.95 (s, 3H), 3.50-3.41 (m, 3H), 3.08 (d, *J* = 13.6 Hz, 1H), 2.96-2.92 (m, 1H), 2.43-2.39 (m, 1H), 1.72 (s(b), 1H), 1.65-1.58 (m, 2H), 1.44-1.40 (m, 1H), 0.78-0.73 (m, 1H), (*Minor Conformer*): δ 9.23 (s, 1H, NH), 8.63 (d, *J* = 4.2 Hz, 1H), 7.95 (d, *J* = 9.2 Hz, 1H), 7.45 (d, *J* = 2.5 Hz, 1H), 7.38-7.36 (m, 1H), 7.22-6.83 (m, 12H), 5.96 (s, 1H, NH), 5.71-5.63 (m, 2H), 3.84 (q, *J* = 9.9 Hz, 1H), 3.50-3.41 (m, 2H), 3.35 (s, 3H), 3.13 (d, *J* = 13.5 Hz, 1H), 2.96-2.92 (m, 1H), 2.43-2.39 (m, 1H), 1.72 (s(b), 1H), 1.65-1.58 (m, 2H), 1.07-1.03 (m, 1H), 0.78-0.73 (m, 1H); **^{13}C-NMR** (151 Mhz, $CDCl_3$) (*Major Conformer*): δ 158.3, 147.6, 144.7, 141.0, 140.9, 140.5, 139.9, 131.8, 129.4, 129.1, 127.7, 122.4, 121.8, 121.2, 119.3, 118.12 (d, J_{PC} = 7.1 Hz), 117.28 (d, J_{PC} = 8.2 Hz), 115.4, 100.9, 71.5, 61.6, 55.9, 55.2, 41.2, 39.2, 27.3, 27.0, 24.9, (*Minor Conformer*): δ 157.6, 147.1, 145.3, 141.1, 140.9, 140.3, 139.6, 131.7, 129.4, 129.1, 126.9, 122.5, 122.4, 121.6, 121.3, 117.6 (d, J_{PC} = 7.0 Hz), 117.4 (d, J_{PC} = 7.9 Hz), 115.2 (d, J_{PC} = 4.9 Hz), 102.4, 80.2, 59.9, 55.1, 54.9, 40.7, 31.1, 27.3, 26.9, 24.8; **^{31}P-NMR** (121 MHz, $CDCl_3$) *(Major Conformer)***:** δ = 1.46 *(Minor Conformer)*: δ = -0.31; **FT-IR (ATR)**: ν [cm^{-1}] : 3155 (m) 2972 (s), 1620 (m) 1602 (m), 1498 (s), 1421 (w), 1219 (m), 1031 (m), 752 (w); **HRMS** (ESI^+): calcd. for [$C_{32}H_{35}N_4O_3P$ + H] 555.2519; found: 555.2519

Catalyst 6 - (N,N'-diphenylphosphorodiamido)-9-Amido-*epi*-quinine

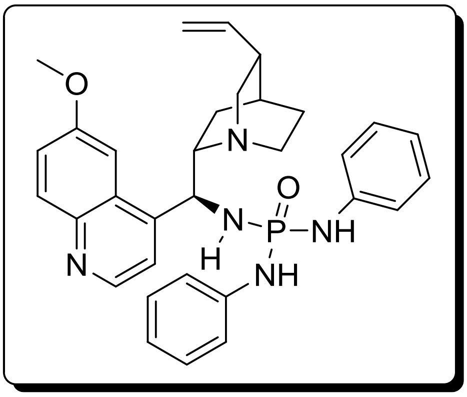

In a Schlenck-tube with magnetic stirring-bar 280 mg (0.86 mmol) 9-*epi*-amino-quinine (9-*epi*-amino-quinine synthesized with an overall yield of 52% following the procedure of Cavaleiro et al.[2]) were dissolved in 3 mL of dry pyridine. At 0°C 229 mg (0.86 mmol) N,N'-diphenylphosphorodiamido-chloridate were added. Stirring was kept up for 1h at 0°C, then at room temperature for further 16 h. Solvent was removed in vacuo and the resulting solid was directly chromatographed on silica gel flash column (EtOAc / MeOH / conc. NH_3 80/20/1; R_f=0.35) to give **6** as a colorless mass. The residue was dissolved in a small amount of dry benzene and frozen in liquid nitrogen thereafter benzene was sublimated in high vacuum yielding **6** as a white solid.

Yield: 373 mg = 78 %; **m.p.** 135 °C; ^{1}**H-NMR** (300 Mhz, DMSO) (*major conformer*): δ = 8.65 (d, *J* = 4.5 Hz, 1H), 8.38 (d, *J* = 9.3 Hz, 1H), 8.26 (d, *J* = 6.6 Hz, 1H), 7.87 (d, *J* = 9.2 Hz, 1H), 7.73 (d, *J* = 1.8 Hz, 1H), 7.51 (d, *J* = 4.5 Hz, 1H), 7.37-7.34 (m, 1H), 7.05-6.97 (m, 8H), 6.74-6.63 (m, 2H), 5.93 (ddd, *J* = 17.4, 10.1, 7.5 Hz, 1H), 5.40 (t, *J* = 9.8 Hz, 1H), 5.11-4.95 (m, 3H), 3.93 (s, 3H), 3.47-3.30 (m, 3H), 2.94-2.77 (m, 2H), 1.56-1.28 (m, 4H), 0.55-0.48 (m, 1H); ^{13}**C-APT** (75 MHz, DMSO) (*Major Conformer*): δ = 157.1, 147.3, 145.7, 145.6, 143.8, 142.4, 142.4, 142.0, 130.9, 128.4, 128.3, 128.2, 127.6, 121.2, 119.3, 119.2, 117.04 (d, $J_{P\text{-}C}$ = 10.7 Hz), 116.9 (d, $J_{P\text{-}C}$ = 7.4 Hz), 114.4, 101.9, 61.2, 55.5, 54.8, 50.1, 39.6, 39.0, 27.0, 27.0, 26.3; ^{31}**P-NMR** (121 Mhz, DMSO) (*major conformer*): δ = 0.83 (m), (*minor conformer*): δ = 2.20 **FT-IR (ATR)**: ν [cm^{-1}] : 3385 (s), 2914 (m), 1620 (m), 1600 (m), 1498 (s), 1284 (m), 1029 (w), 941 (m); **HRMS** (ESI^{+}): calcd. for [$C_{32}H_{36}N_5O_2P$ + H] 554.2679; found: 554.2673; X-ray crystal data: CCDC-958722 (**6**) contains the supplementary crystallographic data for this paper.

Catalyst 7a - (N,N'-diphenylphosphorodiamido)-9-Amido-*epi*-cinchonidine

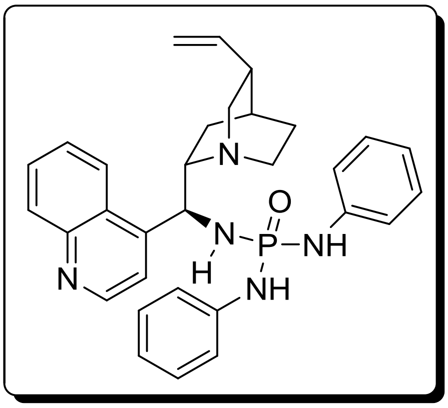

In a Schlenck-tube with magnetic stirring-bar 320 mg (1.09 mmol) 9-*epi*-amino-cinchonidine (9-*epi*-amino-cinchonidine synthesized with an overall yield of 58% following the procedure of Cavaleiro et al.[2]) were dissolved in 4 mL of dry pyridine. At 0°C 291 mg (1.09 mmol) *N,N'*-diphenylphosphorodiamido-chloridate were added. Stirring was kept up for 1 h at 0°C, then at room temperature for further 16 h. Solvent was removed in vacuo and the resulting solid was directly chromatographed on silica gel flash column (EtOAc / MeOH / conc. NH_3 80/20/1; R_f=0.38) to give **7a** as a colorless mass. The residue was dissolved in a small amount of dry benzene and frozen in liquid nitrogen thereafter benzene was sublimated in high vacuum yielding **7a** as a white solid.

Yield: 510 mg = 91 %; **m.p.** 139 °C; **^{1}H-NMR** (300 Mhz, DMSO-d) (*Major Conformer*): δ 8.78 (d, *J* = 4.2 Hz, 1H), 8.37 (t, *J* = 8.8 Hz, 2H), 8.07 (d, *J* = 7.5 Hz, 1H), 7.96 (d, *J* = 8.2 Hz, 1H), 7.69 (t, *J* = 7.5 Hz, 1H), 7.58 (d, *J* = 4.2 Hz, 1H), 7.50 (t, *J* = 7.5 Hz, 1H), 7.08-6.95 (m, 8H), 6.75-6.66 (m, 2H), 5.94-5.83 (m, 1H), 5.39 (t, *J* = 9.0 Hz, 1H), 5.15-4.98 (m, 3H), 3.42-3.25 (m, 3H), 2.92-2.80 (m, 2H), 2.32 (s, 1H), 1.53-1.42 (m, 3H), 1.23-1.16 (m, 1H), 0.51-0.44 (m, 1H); **^{13}C-APT** (75 MHz, DMSO): δ 149.9, 147.6, 147.4, 147.3, 142.3, 141.9, 129.4, 128.7, 128.5, 128.3, 128.2, 126.5, 126.2, 123.7, 119.5, 119.3, 117.1 (d, $J_{P\text{-}C}$ = 7.5 Hz), 116.9 (d, $J_{P\text{-}C}$ = 7.4 Hz), 114.4, 61.5, 54.8, 49.7, 39.7, 39.0, 27.0, 26.9, 26.1; **^{31}P-NMR** (121 Mhz, DMSO) (*major conformer*): δ = 0.61 (m), (*minor conformer*): δ = 1.94 (b) **FT-IR (ATR)**: ν [cm^{-1}] : 3091 (s), 2945 (s), 1635 (s), 1600 (s), 1498 (s), 1417 (m), 1284 (w), 1265 (w), 1203 (m), 1031 (w); **HRMS** (ESI$^+$): calcd. for [$C_{31}H_{34}N_5OP$ + H] 524.2573; found: 524.2566; X-ray crystal data: CCDC-958721 (**7a**) contains the supplementary crystallographic data for this paper.

Catalyst 7b - (N,N'- (3,5-bis(trifluoromethyl)phenylphosphorodiamido)-9-Amido-*epi*-cinchonidine

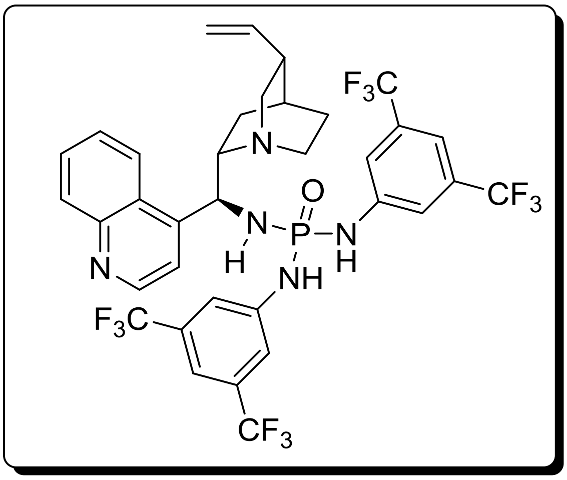

To a solution of 0.16 mL (1 mmol) *m-(CF$_3$)$_2$*-aniline in 5 mL dry benzene in a Schlenck-flask with magnetic stirring-bar and condenser were added 0.034 mL Et_3N (0.25 mmol) and 0.046 mL $POCl_3$ (0.5 mmol) successively via syringe and the resulting suspension was refluxed for 48 h until evolution of HCl-gas had ceased. The solvent was removed in vacuo and the resulting solid suspended in 2 mL of dry THF. Further 0.069 mL (0.5 mmol) Et_3N were added and the suspension was cooled to 0 °C. To this was added dropwise a solution of 146 mg (0.5 mmol) 9-*epi*-amino-cinchonidine in 2 mL THF via syringe. The suspension was allowed to warm to rt and stirred for further 16 h. Solvents were removed in vacuo and the residue was subjected to flash column chromatography over silica (EtOAc / cHex, 50 /50; R_f = 0.12) to give **7b** as a colorless mass. The residue was dissolved in a small amount of dry benzene and frozen in liquid nitrogen, thereafter benzene was sublimated in high vacuum yielding **7b** as a white solid.

Yield: 123 mg = 31 %; **m.p.** 120 °C; ^{1}**H-NMR** (400 MHz, DMSO) (*major conformer*): δ = 8.94 (d, *J* = 6.8 Hz, 1H), 8.86 – 8.75 (m, 1H), 8.67 (d, *J* = 4.3 Hz, 1H), 8.18 (d, *J* = 8.3 Hz, 1H), 7.87 (d, *J* = 8.4 Hz, 1H), 7.64 (t, *J* = 7.6 Hz, 1H), 7.61 – 7.42 (m, 4H), 7.36 – 7.25 (m, 4H), 6.29 – 6.17 (m, 1H), 5.85 (ddd, *J* = 17.4, 10.1, 7.6 Hz, 1H), 5.14 – 4.92 (m, 2H), 3.49 – 3.17 (m, 3H), 2.84 – 2.64 (m, 2H), 2.37 – 2.25 (m, 1H), 1.60 – 1.45 (m, 3H), 1.22 (t, *J* = 12.3 Hz, 1H), 0.46 (dd, *J* = 12.5, 8.0 Hz, 1H); ^{13}**C-NMR** (101 MHz, DMSO) (*major conformer*): δ = 149.7, 147.5, 146.6, 144.0, 143.7, 141.8, 130.5, 130.4, 129.4, 128.5, 126.28, 126.1, 123.1, 122.7, 119.2, 117.1 (d, $J_{P\text{-}C}$ = 7.5 Hz), 116.7 (d, $J_{P\text{-}C}$ = 7.5 Hz), 114.2, 112.3, 60.6, 54.8, 49.5, 39.7, 38.9, 27.0, 26.9, 25.9; ^{31}**P- NMR** (162 MHz, DMSO) (*major conformer*): δ = -0.39 (*minor conformer*): 1.19; ^{19}**F-NMR** (376 MHz, DMSO) (*major* conformer): δ = -61.71, -61.81 (*minor conformer*): -61.93, -62.04; **FT-IR (ATR)**: ν [cm^{-1}] : 3156 (s), 2926 (s), 1620 (m), 1469 (m), 1377 (s) 1276 (s), 1176 (m), 1132 (s), 1001 (w), 979 (m)

Catalyst 7c - (N,N'- (3,5-dichloro-phenyl)phosphorodiamido)-9-Amido-*epi*-cinchonidine

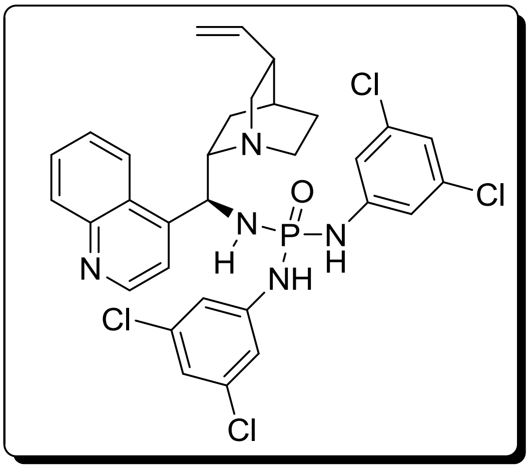

To a solution of 162 mg (1 mmol) *m-Cl*$_2$-aniline in 5 mL dry benzene in a Schlenck-flask with magnetic stirring-bar and condenser were added 0.034 mL Et_3N (0.25 mmol) and 0.046 mL $POCl_3$ (0.5 mmol) successively via syringe and the resulting suspension was refluxed for 24 h until evolution of HCl-gas had ceased. The solvent was removed in vacuo and the resulting solid suspended in 2 mL of dry THF. Further 0.069 mL (0.5 mmol) Et_3N were added and the suspension was cooled to 0 °C. To this was added dropwise a solution of 146 mg (0.5 mmol) 9-*epi*-amino-cinchonidine in 2 mL THF via syringe. The suspension was allowed to warm to rt and stirred for further 16 h. Solvents were removed in vacuo and the residue was subjected to flash column chromatography over silica (EtOAc / MeOH, 98 / 2; R_f = 0.27) to give **7c** as a colorless mass. The residue was dissolved in a small amount of dry benzene and frozen in liquid nitrogen, thereafter benzene was sublimated in high vacuum yielding **7c** as a white solid.

Yield: 116 mg = 35 %; **m.p.** 162 °C; **^{1}H-NMR** (400 MHz, DMSO) (*major conformer*): δ = 8.85 – 8.15 (m, 4H), 7.96 (d, *J* = 7.9 Hz, 1H), 7.79 – 7.42 (m, 3H), 7.08 (m, 2H), 6.88 – 6.71 (m, 4H), 5.99 – 5.80 (m, 2H), 5.11 – 4.96 (m, 3H), 3.42 – 3.32 (m, 3H), 2.91 – 2.69 (m, 2H), 2.39 – 2.27 (m, 1H), 1.63 – 1.42 (m, 3H), 1.31 – 1.19 (m, 1H), 0.50 (s, 1H); **^{13}C-APT** (151 MHz, DMSO)) (*major conformer*): δ = 149.8, 147.6, 146.7, 144.6, 144.4, 141.9, 133.8, 133.8, 129.5, 128.8, 126.4, 126.2, 123.1, 119.3, 119.1, 118.9, 115.7 (d, J_{P-C} = 7.5 Hz), 115.3 (d, J_{P-C} = 7.5 Hz), 114.4, 60.8, 54.8, 49.6, 38.9, 36.2, 27.0, 25.9, 24.2; **^{31}P- NMR** (162 MHz, DMSO) (*major conformer*): δ = -0.28 (*minor conformer*): 0.93; **FT-IR (ATR)**: ν [cm^{-1}] : 3371 (s), 2945 (m), 1598 (s), 1577 (m), 1442 (w), 1112 (w), 979 (m)

Catalyst 7d - (N,N'-(3,5-fluoro-phenyl)phosphorodiamido)-9-Amido-*epi*-cinchonidine

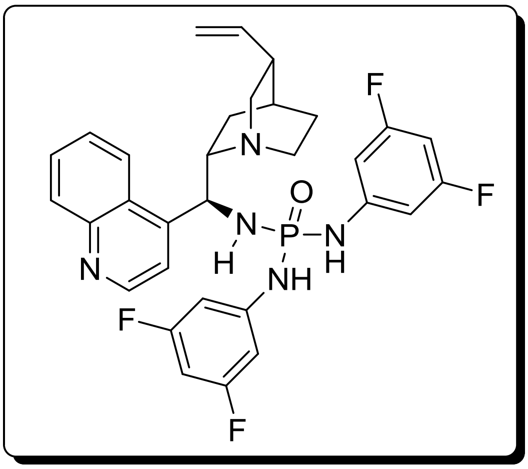

To a solution of 129 mg (1 mmol) *m-F*$_2$-aniline in 5 mL dry benzene in a Schlenck-flask with magnetic stirring-bar and condenser were added 0.034 mL Et_3N (0.25 mmol) and 0.046 mL $POCl_3$ (0.5 mmol) successively via syringe and the resulting suspension was refluxed for 24 h until evolution of HCl-gas had ceased. The solvent was removed in vacuo and the resulting solid suspended in 2 mL of dry THF. Further 0.069 mL (0.5 mmol) Et_3N were added and the suspension was cooled to 0 °C. To this was added dropwise a solution of 146 mg (0.5 mmol) 9-*epi*-amino-cinchonidine in 2 mL THF via syringe. The suspension was allowed to warm to rt and stirred for further 16 h. Solvents were removed in vacuo and the residue was subjected to flash column chromatography over silica (EtOAc / MeOH, 95 / 5; R_f = 0.30) to give **7d** as a colorless mass. The residue was dissolved in a small amount of dry benzene and frozen in liquid nitrogen, thereafter benzene was sublimated in high vacuum yielding **7d** as a white solid.

Yield: 66 mg = 22 %; **m.p.** 142-146 °C; **^{1}H-NMR** (400 MHz, DMSO) (*major conformer*): δ = 8.85 – 8.15 (m, 4H), 7.96 (d, *J* = 7.9 Hz, 1H), 7.79 – 7.42 (m, 3H), 7.08 (m, 2H), 6.88 – 6.71 (m, 4H), 5.99 – 5.80 (m, 2H), 5.11 – 4.96 (m, 3H), 3.42 – 3.32 (m, 3H), 2.91 – 2.69 (m, 2H), 2.39 – 2.27 (m, 1H), 1.63 – 1.42 (m, 3H), 1.31 – 1.19 (m, 1H), 0.50 (s, 1H); **^{13}C-NMR** (101 MHz, DMSO) (*major conformer*): δ = 162.6, 162.5, 149.8, 147.6, 146.7 (d, J_{P-C} = 4.1 Hz), 144.9 (d, *J* = 9.4 Hz), 141.9, 131.4, 131.3, 129.4, 128.7, 128.6, 126.3, 123.2, 119.3, 114.3, 100.3, 100.3, 100.0, 99.9, 94.8, 94.6, 61.0, 61.0, 54.6, 49.6, 39.9, 27.0, 26.9, 25.9; **^{31}P- NMR** (162 MHz, DMSO) (*major conformer*): δ = -0.28 (*minor conformer*): 0.93; **^{19}F NMR** (376 MHz, DMSO) (*major conformer*): δ = -110.36, -110.47; **FT-IR (ATR)**: ν [cm^{-1}] : 3163 (m), 2943 (m), 1624 (s), 1600 (m), 1514 (m), 1475 (m), 1201 (w), 1151 (s), 1114 (s), 1028 (m)

Catalyst 7e - (N,N'-(4-nitro-phenyl)phosphorodiamido)-9-Amido-*epi*-cinchonidine

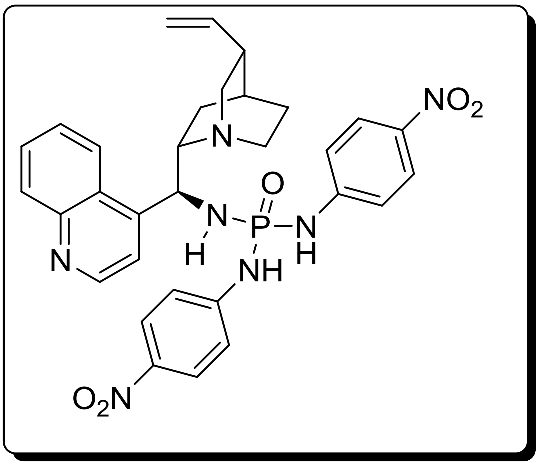

To a solution of 138 mg (1 mmol) *p-(NO$_2$)*-aniline in 5 mL dry benzene in a Schlenck-flask with magnetic stirring-bar and condenser were added 0.034 mL Et_3N (0.25 mmol) and 0.046 mL $POCl_3$ (0.5 mmol) successively via syringe and the resulting suspension was refluxed for 48 h until evolution of HCl-gas had ceased. The solvent was removed in vacuo and the resulting solid suspended in 2 mL of dry THF. Further 0.069 mL (0.5 mmol) Et_3N were added and the suspension was cooled to 0 °C. To this was added dropwise a solution of 146 mg (0.5 mmol) 9-*epi*-amino-cinchonidine in 2 mL THF via syringe. The suspension was allowed to warm to rt and stirred for further 16 h. Solvents were removed in vacuo and the residue was subjected to flash column chromatography over silica (EtOAc / MeOH, 80 / 20; R_f = 0.31) to give **7e** as a yellow solid.

Yield: 95 mg = 31 %; **m.p.** 178- °C (decomp); **^{1}H-NMR** (300 MHz, DMSO) (*major* conformer): δ = 9.68 (s, 1H), 9.24 (s, 1H), 8.78 (d, *J* = 4.4 Hz, 1H), 8.16 (d, *J* = 8.4 Hz, 1H), 8.03 – 7.84 (m, 5H), 7.66 (t, *J* = 7.5 Hz, 1H), 7.56 (d, *J* = 4.5 Hz, 1H), 7.46 (t, *J* = 7.5 Hz, 1H), 7.16 (d, *J* = 9.0 Hz, 2H), 7.07 (d, *J* = 9.0 Hz, 2H), 6.04 (t, *J* = 10.0 Hz, 1H), 5.91 (ddd, *J* = 17.4, 10.2, 7.5 Hz, 1H), 5.11 – 5.01 (m, 3H), 3.51 – 3.36 (m, 3H), 2.92 – 2.88 (m, 2H), 2.36 (s, 1H), 1.57 – 1.55 (m, 3H), 1.30 – 1.23 (m, 1H), 0.59 – 0.42 (m, 1H); **^{13}C-APT** (126 MHz, DMSO) (*major conformer*): δ = 150.5, 149.6, 148.1, 146.94, 146.90, 142.3, 140.4, 140.3, 129.9, 129.2, 126.8, 126.6, 125.5, 125.3, 123.6, 119.7, 117.4 (d, $J_{P\text{-}C}$ = 7.6 Hz), 117.2 (d, $J_{P\text{-}C}$ = 7.6 Hz), 115.0, 61.6, 55.0, 50.2, 40.6, 39.3, 27.4, 26.5, 24.7; **^{31}P- NMR** (162 MHz, DMSO) (*major conformer*): δ = -1.02 (*minor conformer*): 0.66; **FT-IR (ATR)**: ν [cm^{-1}] : 3140 (m), 2943 (m), 1593 (s), 1514 (s), 1336 (s), 1294 (s), 1190 (w), 1111 (m), 993 (m), 846 (w)

Catalyst 7f - (N,N'-(4-methoxy-phenyl)phosphorodiamido)-9-Amido-*epi*-cinchonidine

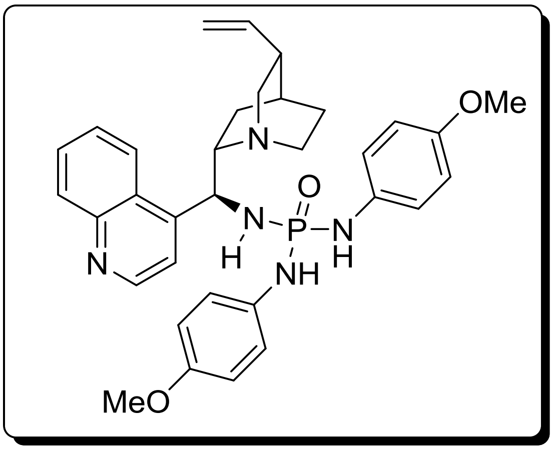

To a solution of 123 mg (1 mmol) *p-(OMe)*-aniline in 5 mL dry benzene in a Schlenck-flask with magnetic stirring-bar and condenser were added 0.034 mL Et_3N (0.25 mmol) and 0.046 mL $POCl_3$ (0.5 mmol) successively via syringe and the resulting suspension was refluxed for 24 h until evolution of HCl-gas had ceased. The solvent was removed in vacuo and the resulting solid suspended in 2 mL of dry THF. Further 0.069 mL (0.5 mmol) Et_3N were added and the suspension was cooled to 0 °C. To this was added dropwise a solution of 146 mg (0.5 mmol) 9-*epi*-amino-cinchonidine in 2 mL THF via syringe. The suspension was allowed to warm to rt and stirred for further 16 h. Solvents were removed in vacuo and the residue was subjected to flash column chromatography over silica (EtOAc / MeOH, 85 / 15; R_f = 0.28) to give **7f** as a white solid.

Yield: 125 mg = 43 %; **m.p.** 115 °C; **^{1}H-NMR** (300 MHz, DMSO) (*major conformer*): δ = 8.78 (d, *J* = 4.1 Hz, 1H), 8.32 (d, *J* = 8.4 Hz, 1H), 8.03 – 7.93 (m, 2H), 7.70 (t, *J* = 7.3 Hz, 4H), 7.60 – 7.46 (m, 4H), 6.89 (t, *J* = 8.8 Hz, 4H), 6.66 (d, *J* = 8.6 Hz, 2H), 6.58 (d, *J* = 8.6 Hz, 2H), 5.95 – 5.79 (m, 1H), 5.27 – 4.95 (m, 4H), 3.63 (s, 3H), 3.58 (s, 3H), 3.41 – 3.19 (m, 2H), 2.91 – 2.71 (m, 2H), 2.36 – 2.22 (m, 1H), 1.52 – 1.50 (m, 3H), 1.23 – 1.17 (m, 1H), 0.88 – 0.72 (m, 1H), 0.52 – 0.40 (m, 1H). **^{13}C-APT** (75 MHz, DMSO) (*major* conformer): δ = 153.0, 152.9, 149.9, 147.6, 141.9, 135.6, 129.4, 128.7, 126.5, 126.2, 123.7, 119.4, 118.3, 118.1, 114.4, 113.9, 113.8, 61.7, 55.1, 55.1, 54.8, 49.8, 39.9, 39.0, 27.1, 26.9, 26.0; **^{31}P- NMR** (121 MHz, DMSO) (*major conformer*): δ = 1.56 (*minor conformer*): 2.88; **FT-IR (ATR)**: ν [cm^{-1}] : 3228 (s), 2941 (w), 1635 (m), 1508 (s), 1280 (w), 1238 (m), 1111 (w), 1035 (w), 950 (w)

2,2,2,4,4,4-hexachloro-1,3-diphenyl-1,3,2,4-diazaphosphetidin

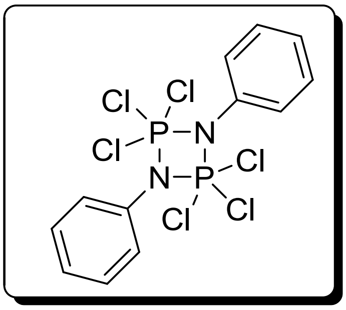

In a 100-mL three-neck-flask with condenser and dropping-funnel 8.33 g PCl_5 (40 mmol) were suspended in 20 mL dry benzene. Aniline (3.65 mL, 40 mmol) in 40 mL benzene was added dropwise over a period of 30 min, the resulting yellowish suspension was then refluxed for 3 h until HCl-evolution had ceased. The solution was left to stand overnight and the resulting white precipitate was filtered off and washed with 3x 20 mL benzene yielding 6.82 g (75 %) of product. Melting point matched the literature-value [4] and the product was used without further purification.

Yield: 6.82 g = 75 %; **m.p.** 182-186 °C

12 - 2,4-dichloro-1,3-diphenyl-1,3,2,4-diazaphosphetidin-2,4-disulfide

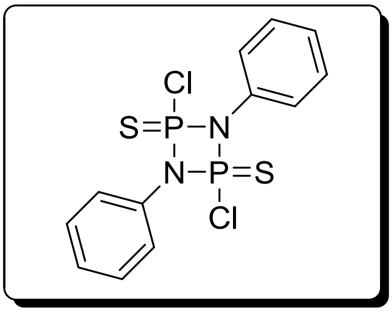

In a gas-washing bottle with magnetic stirring bar 6 g (13,2 mmol) 2,2,2,4,4,4-hexachloro-1,3-diphenyl-1,3,2,4-diazaphosphetidine were suspended in 70 mL dry benzene and 4.26 mL (52.9 mmol) dry pyridine were added. For 4 h a stream of dry H_2S-gas was bubbled through the suspension. The suspension was filtered under argon and solvent was removed in vacuo. The residue was then recrystallized twice from dry benzene (2x 15 mL) at 4 °C giving **12***benzene as colorless needles, which upon removal of benzene in vacuo yielded 2 g of white **12**.

Yield: 2g = 40 % (5/1 mixture of *cis*/*trans*-isomers as determined by X-ray) ; **m.p.** 146 °C; ^{1}**H-NMR** (300 MHz, $CDCl_3$) (cis/trans overlapping): δ = 7.69 - 7.66 (m, 2H), 7.53 (t, *J* = 7.3 Hz, 2H), 7.44 - 7.42 (m, 1H); ^{13}**C-APT** (75 MHz, $CDCl_3$) (cis): δ = 132.2, 130.2, 127.7, 122.9 (t, *J* = 6.5 Hz); ^{31}**P-NMR** (121 MHz, $CDCl_3$): δ = 38.97 (trans), 36.83 (cis)

Catalyst 14a/b - *cis/trans*-2,4-bis(((*1R,2R*)-2-(dimethylamino)cyclohexyl)amino)-1,3-diphenyl-1,3,2,4-diazadiphosphetidine-2,4-disulfide

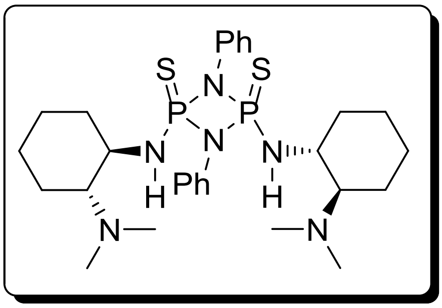

To a stirred solution of (*R*,*R*)-*N*,*N'*-dimethyl-cyclohexane-1,2-diamin (400 mg, 2.81 mmol) in DCM (4 mL) was added a solution of 2,4-dichloro-1,3-diphenyl-cyclodiphosphazane-2,4-disulfide (532 mg, 1.40 mmol) in DCM (2 mL) via syringe at 0 °C. After 0.5 h, Et_3N (284 mg, 2.81 mmol) was added via syringe and the mixture was kept stirring at 0 °C for further 0.5 h. The reaction was allowed to warm to rt and stirred for 1h at this temperature. The solvent was removed in vacuo and the yellowish residue was purified by column chromatography over neutral alumina (grade V, EtOAc/Hexane 1:7, R_f-*trans*: 0.71, Rf-*cis*: 0.32) to yield 171 mg (21%) of *cis*-**14a** and 228mg (28%) of *trans*-**14b** as white solids.

Cis-**14a**: **Yield:** 171 mg (21%); **m.p.** 110- °C (decomp); ^{1}**H-NMR** (300 Mhz, $CDCl_3$): δ = 7.56 (d, *J* = 7.8 Hz, 4H), 7.30 (t, *J* = 7.8 Hz, 4H), 7.07 (t, *J* = 7.4 Hz, 2H), 4.93 (s, 2H, NH), 3.14 (s, 2H), 2.54 (s, 2H), 2.21-2.15 (m, 2H), 2.09 (s, 12H, CH_3), 1.82-1.61 (m, 6H), 1.26-1.07 (m, 8H); ^{13}**C-APT** (75 MHz, $CDCl_3$) δ = 136.3, 129.3, 123.8, 119.7, 68.1, 54.9, 40.5, 34.6, 25.3, 24.8, 21.5; ^{31}**P-NMR** (121 Mhz, $CDCl_3$): δ = 46.80 (b) **FT-IR (ATR)**: ν [cm^{-1}] : 3049 (s), 2933 (s), 2860 (m), 1635 (m), 1598 (s), 1496 (s), 1282 (m), 1132 (w), 1099 (m), 952 (m) **HRMS** (ESI^+): calcd. for [$C_{28}H_{44}N_6P_2S_2$ + H] 591.2616; found: 591.2610; X-ray crystal data: CCDC-958718 (**14a**) contains the supplementary crystallographic data for this paper.

Trans-**14b**: **Yield:** 228 mg (28%); **m.p.** 115- °C (decomp); ^{1}**H-NMR** (300 MHz, $CDCl_3$): δ = 7.74 (d, *J* = 7.9 Hz, 2H), 7.58 (d, *J* = 8.0 Hz, 2H), 7.39 – 7.30 (m, 4H), 7.08 (dd, *J* = 15.5, 7.5 Hz, 2H), 5.17 (s, 2H, NH), 3.21 – 2.88 (m, 2H), 2.51 – 2.41 (m, 2H), 2.15 – 2.06 (m, 2H), 1.98 (s, 12H), 1.69 (t, *J* = 10.8 Hz, 4H), 1.49 – 1.41 (m, 2H), 1.20 – 0.83 (m, 8H); ^{13}**C-APT** (75 MHz, $CDCl_3$): δ = 136.8 (d, J_{P-C} = 24.3 Hz), 129.3, 123.2 (d, J_{P-C} = 17.2 Hz), 118.3 (dt, J_{P-C} = 19.6, 7.6 Hz), 68.0, 67.9, 67.8, 54.4, 40.4, 33.4, 25.2, 24.3, 21.4; ^{31}**P-NMR** (121 Mhz, $CDCl_3$): δ = 42.49 (b)

1,3-di-tert-butyl-2,4-dichloro-1,3,2,4-diazadiphosphetidine

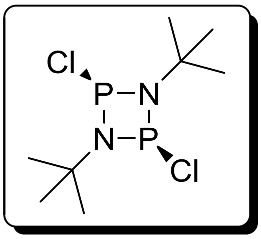

The title-compound was prepared following a procedure of *Wright et al.*[5].

Yield: 63 %; ^{1}**H-NMR** (300 MHz, $CDCl_3$): δ = 1.39 (s, 18H); ^{13}**C-APT** (75 MHz, $CDCl_3$): δ = 54.5 (t, *J* = 6.7 Hz), 30.6 (t, *J* = 6.2 Hz); ^{31}**P-NMR** (122 MHz, $CDCl_3$): δ = 207.65.

Catalyst 15 - *cis*-1,3-di-tert-butyl-2,4-bis(((*1R,2R*)-2-(dimethylamino)cyclohexyl)amino)-1,3,2,4-diazadiphosphetidine-2,4-disulfide

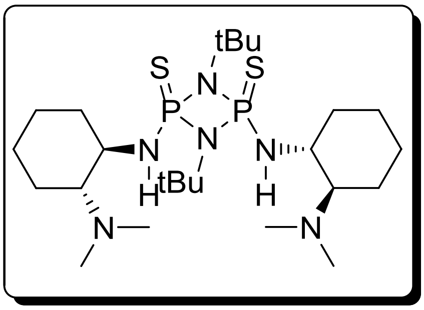

A solution of (*R*,*R*)-*N*,*N'*-dimethyl-cyclohexane-1,2-diamine (200 mg, 1.4 mmol) and Et_3N (284 mg, 1.4 mmol) in Et_2O (2 mL) was added dropwise to a solution of *cis*-($^tBuNPCl)_2$ (196 mg, 0.7 mmol) in Et_2O (4 mL) at 0 °C. After stirring at this temperature for 1h, the mixture was allowed to warm to room temperature and stirred for further 16 h. The resulting suspension was filtered under argon and the filtrate concentrated in vacuo. The residue was redissolved in toluene (5 mL), elemental sulphur was added (90 mg, 2.8 mmol) and stirred for 16 h at 50 °C. Solvent was removed in vacuo and the crude product was purified by column chromatography on silica (EtOAc/MeOH/Net_3 80/20/1 R_f: 0.15) yielding 54% (210 mg, 0.76 mmol) of **15** as a white solid.

Yield: 210 mg (54 %); **m.p.** 205 °C; **^{1}H-NMR** (300 Mhz, $CDCl_3$): δ = 4.50(s, 2H, NH), 3.04 (s, 2H), 2-84-2.81 (m, 2H), 2.18 (s, 12H), 2.11 (t, J = 9.0 Hz, 2H), 1.86-1.73 (m, 4H), 1.63 (s, 2H), 1.58 (s, 18H), 1.28-1.11 (m, 8H); **^{13}C-APT** (75 MHz, $CDCl_3$) δ = 68.4 (t, J_{PC} = 5.9 Hz), 56.8, 55.1, 41.2, 33.8, 30.2 (t, J_{PC} = 4.6 Hz), 25.4, 24.6, 21.7; **^{31}P{^{1}H}-NMR** (121 Mhz, $CDCl_3$): δ = 46.37; **FT-IR (ATR)**: ν [cm^{-1}] : 2985 (s), 1639 (s), 1531 (s), 1512 (m), 1400 (m), 1242 (w), 1002 (w); **HRMS** (ESI^+): calcd. for [$C_{24}H_{52}N_6P_2S_2$ + H] 551.3242; found: 551.3237; X-ray crystal data: CCDC-958719 (**15**) contains the supplementary crystallographic data for this paper.

Catalyst 16 - 1,3-di-tert-butyl-2-(((*1R,2R*)-2-(dimethylamino)cyclohexyl)amino)-4-(phenylamino)-1,3,2,4-diazadiphosphetidine-2,4-disulfide

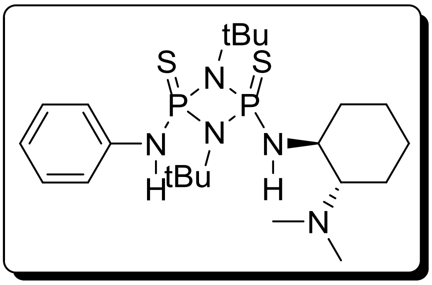

A solution of aniline (130 mg, 1.4 mmol) and Et_3N (141 mg, 1.4 mmol) in THF (4 mL) was added dropwise to a solution of *cis*-($^tBuNPCl)_2$ (385 mg, 1.4 mmol) in THF (10 mL) at -78 °C. After stirring at this temperature for 1h, the mixture was allowed to warm to room temperature and stirred for further 16 h. To the suspension was then added a solution of (*R*,*R*)-*N*,*N'*-dimethyl-cyclohexan-1,2-diamin (199 mg, 1.4 mmol) and Et_3N (141 mg, 1.4 mmol) in THF (2 mL) at -78 °C. After 0.5h the mixture was allowed to warm to rt and stirred over night. The resulting suspension was filtered under argon and the filtrate concentrated in vacuo. The residue was redissolved in toluene (10 mL), elemental sulphur was added (96 mg, 3 mmol) and stirred for 16 h at 50 °C. Solvent was removed in vacuo and the crude product was purified by column chromatography on silica (gradient EtOAc/n-hexane 1:1 to EtOAc) yielding 26% (183 mg, 0.36 mmol) of **16** as a white solid.

Yield: 183 mg (26%); **m.p.** 191 °C; **^{1}H-NMR** (300 Mhz, $CDCl_3$): δ = 7.26 (t, *J* = 7.8 Hz, 2H), 7.15 (d, *J* = 8.2 Hz, 2H), 7.03 (t, *J* = 7.3 Hz, 1H), 5.35 (d, J_{PH} = 13.6 Hz, 1H, NH), 4.24 (s, 1H, NH)), 3.19-3.10 (m, 1H), 2.87-2.83 (m, 1H), 2.10 (m, 1H), 2.08 (s, 6H), 1.72 (m, 2H), 1.56-1.50 (m, 10H), 1.45 (s, 9H), 1.23-1.02 (m, 4H); **^{13}C APT** (75 MHz, $CDCl_3$) δ = 140.0 (d, J_{PC} = 7.0 Hz), 129.7, 123.8, 120.5 (d, J_{PC} = 5.5 Hz), 68.5 (d, J_{PC} = 11.3 Hz), 57.5, 56.9, 55.3, 41.5, 35.0, 29.9, 29.8, 25.4, 24.6, 21.8; **^{31}P{^{1}H}-NMR** (121 Mhz, $CDCl_3$): δ = 47.74 (d, J_{PP} = 35.8 Hz), 38.83 (d, J_{PP} = 35.8 Hz) **FT-IR (ATR)**: 3248 (s), 2974 (m), 2937 (m) , 2868 (w), 1598 (w), 1494 (m), 1386 (m), 1369 (m), 1055 (s), 902 (s); **HRMS** (ESI^+): calcd. for [$C_{22}H_{41}N_5P_2S_2$ + H] 502.2351; found: 502.2344; X-ray crystal data: CCDC-958720 (**16**) contains the supplementary crystallographic data for this paper.

DOSY and Variable temperature NMR

With epimerization of quinine/cinchonidine-derived catalysts at C9 (Catalyst **5-7f**), NMR-Spectra show a dual set of signals the ratio of which was solvent-dependent. We were able to attribute this behaviour to conformational isomerism by means of Diffusion Ordered NMR and variable temperature experiments with compound **7a**. While coalescence could be achieved at 328 K on a 200 Mhz-instrument in toluene-d, shim and signal/noise ratio was generally bad. We thus chose to employ DMSO-d_6 for all measurements with **6-7f**, as signal-ratio (major/minor) for **6-7f** is generally between 20/1-15/1 in this solvent and exchanging NH-protons are well visible. Minor conformer-peaks are given where they could be assigned properly.

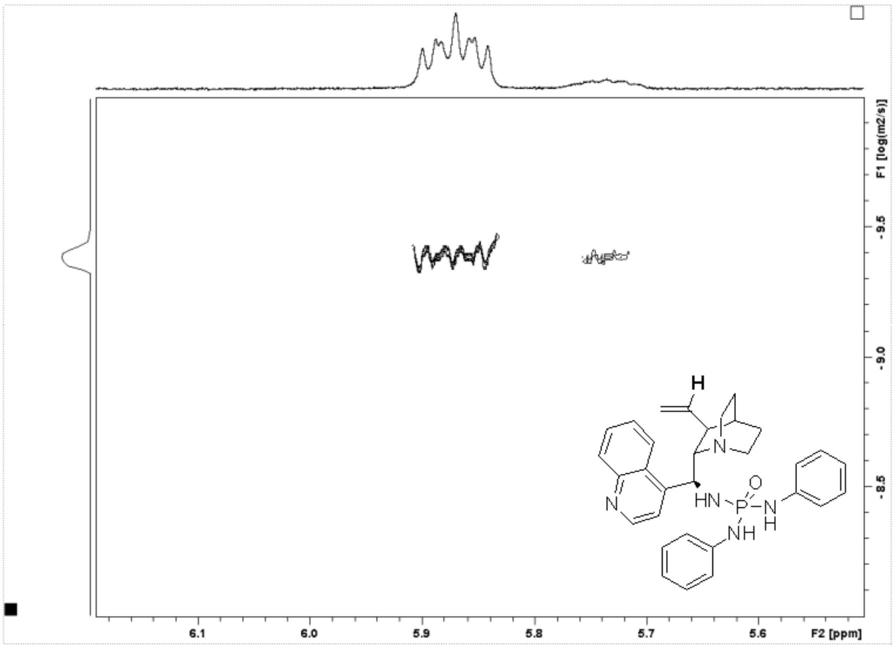

DOSY-Spectrum of **7a**: Dual-peak for the highlighted proton (bold), shows only one compound.

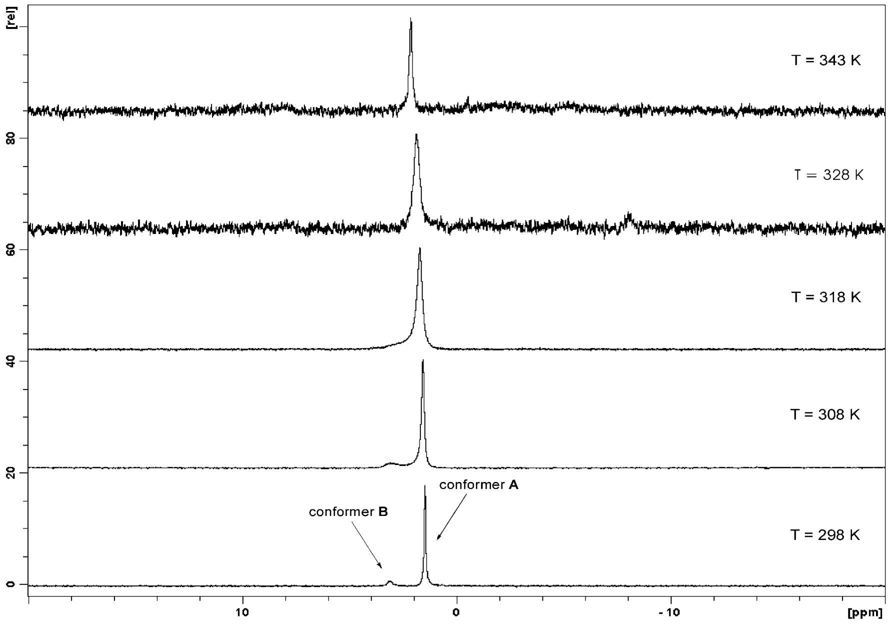

Temperature dependent ^{31}P-NMR of **7a** in toluene: Coalescence at 328 K.

NMR-Spectra

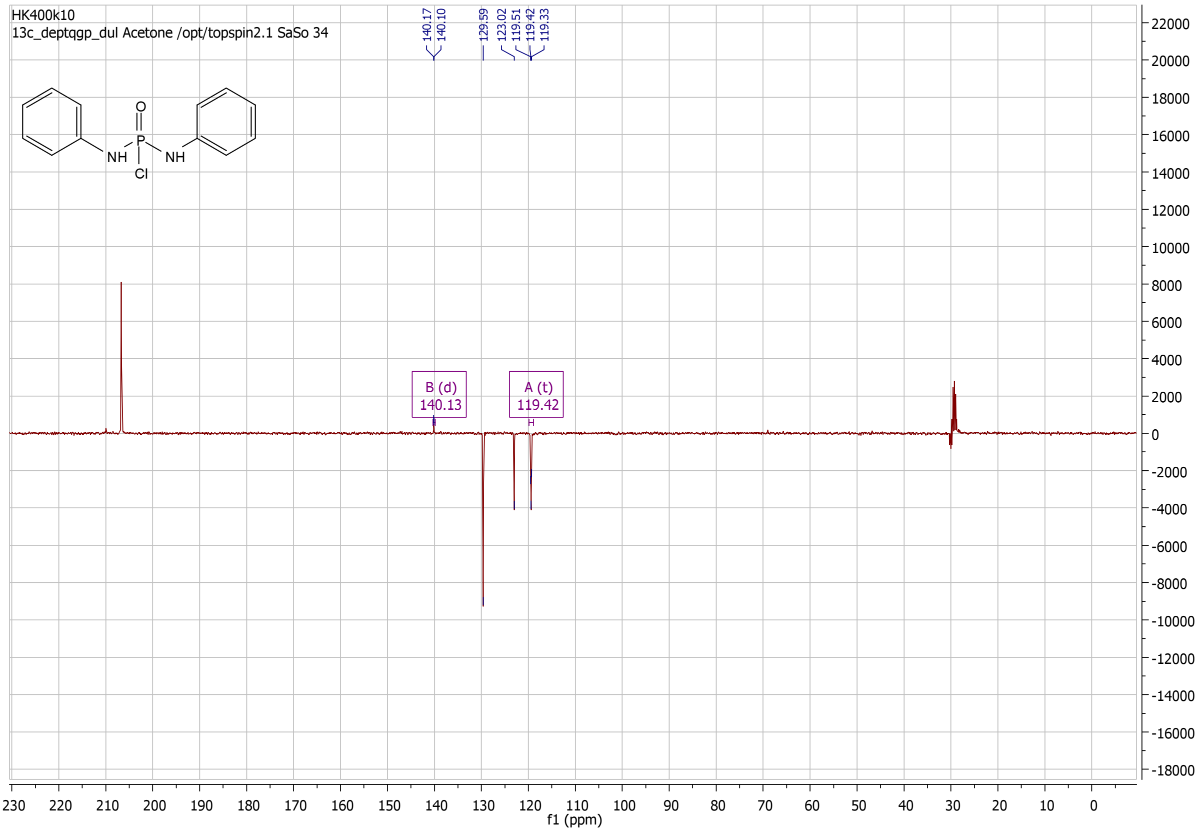
HK400k10
13c_deptqgp_dul Acetone /opt/topspin2.1 SaSo 34
140.17
140.10
129.59
123.02
119.51
119.42
119.33
O
P
NH
NH
Cl
B (d)
140.13
A (t)
119.42
f1 (ppm)

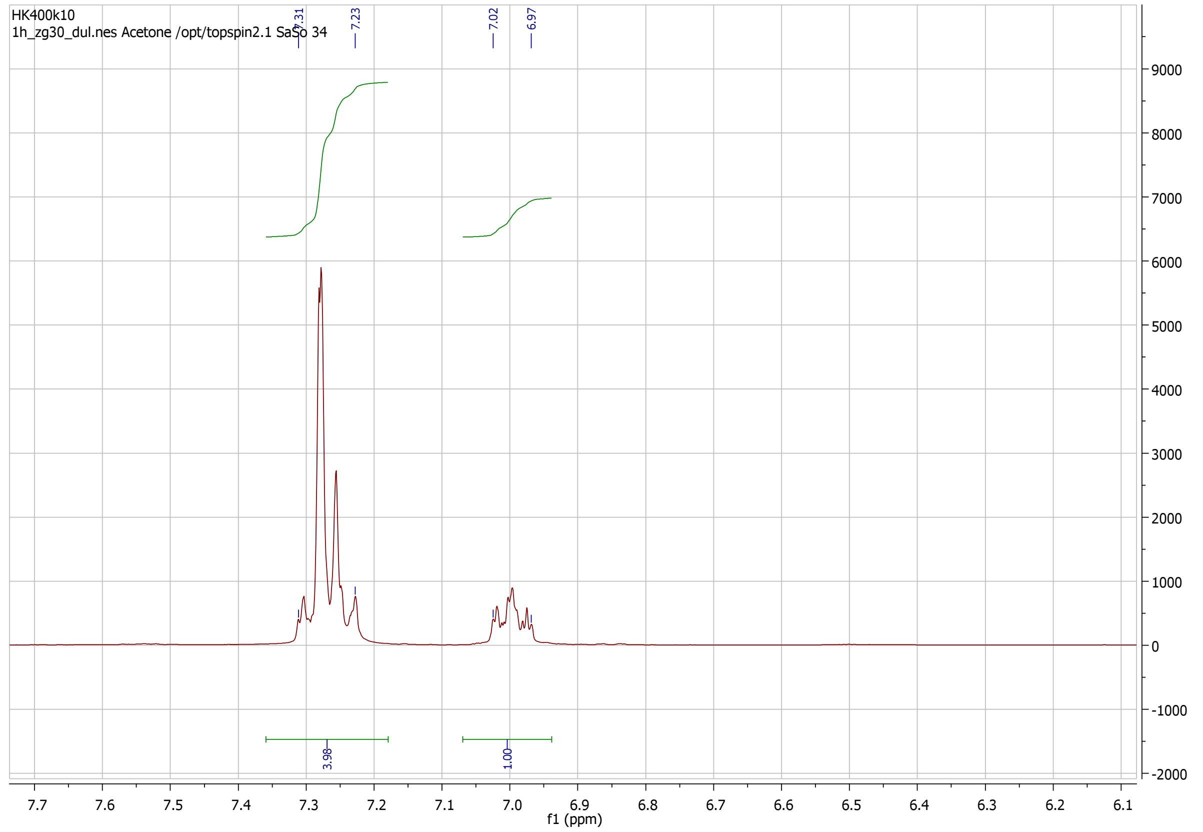
HK400k10
1h_zg30_dul.nes Acetone /opt/topspin2.1 SaSo 34
7.31
7.23
7.02
6.97
3.98
1.00
f1 (ppm)

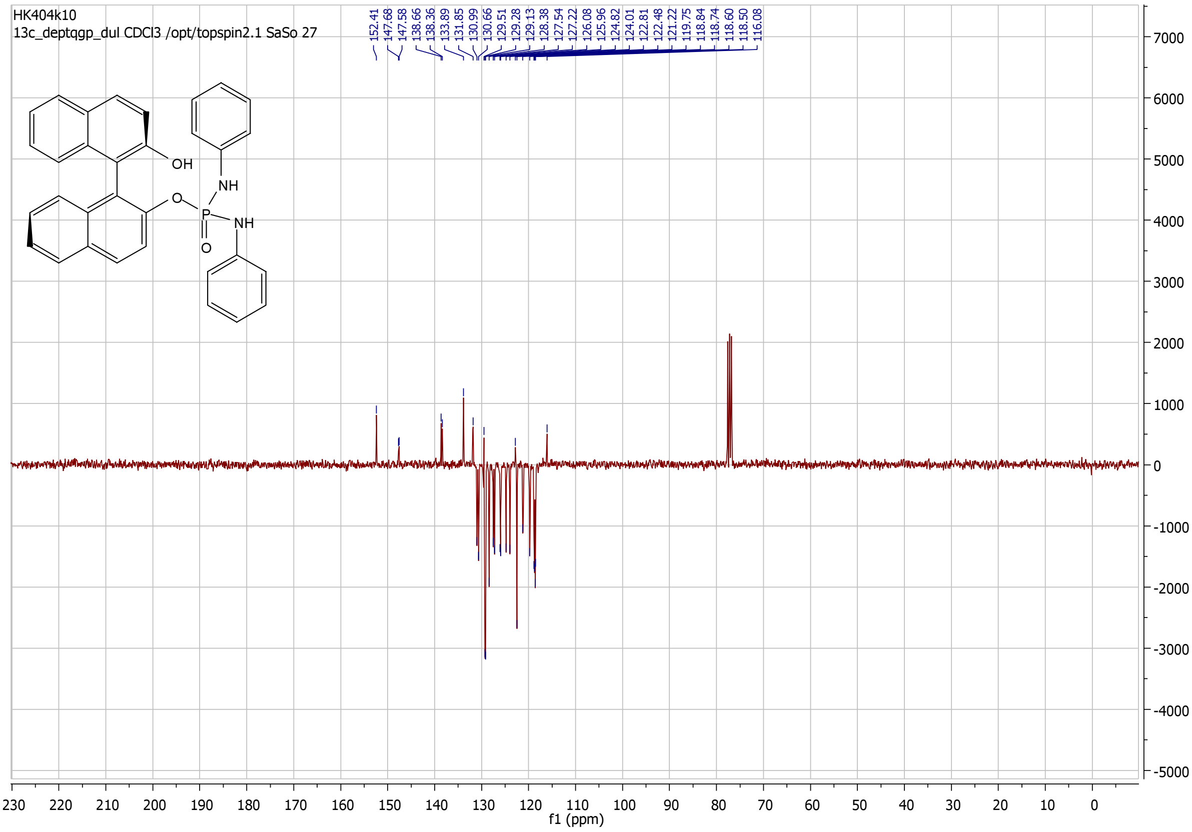
HK404k10
13c_deptqgp_dul CDCl3 /opt/topspin2.1 SaSo 27
152.41
147.68
147.58
138.66
138.36
133.89
131.85
130.99
130.66
129.51
129.28
129.13
128.38
127.54
127.22
126.08
125.96
124.82
124.01
122.81
122.48
121.22
119.75
118.84
118.74
118.60
118.50
116.08
OH
O
NH
P
NH
O
f1 (ppm)

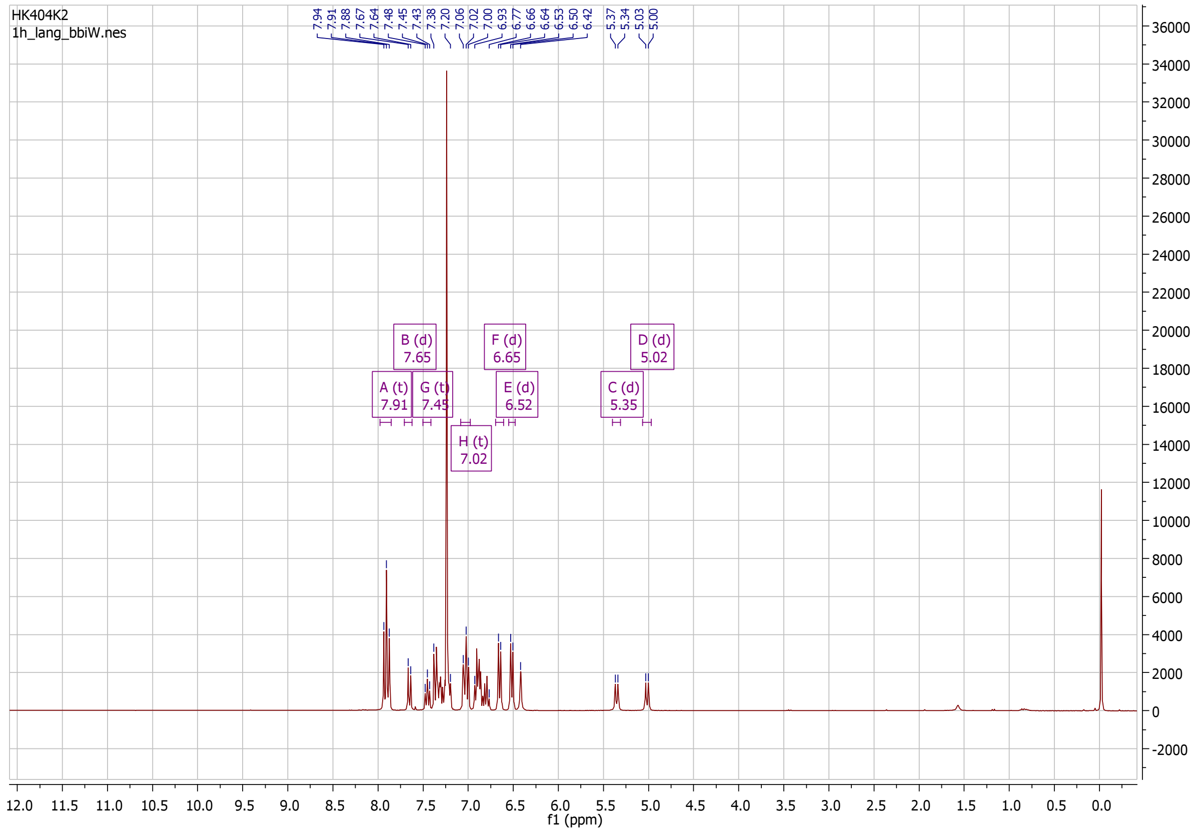
HK404K2
1h_lang_bbiW.nes
7.94
7.91
7.88
7.67
7.64
7.48
7.45
7.43
7.38
7.20
7.06
7.02
7.00
6.93
6.77
6.66
6.64
6.53
6.50
6.42
5.37
5.34
5.03
5.00
B (d)
7.65
F (d)
6.65
D (d)
5.02
A (t)
7.91
G (t)
7.45
E (d)
6.52
C (d)
5.35
H (t)
7.02
f1 (ppm)

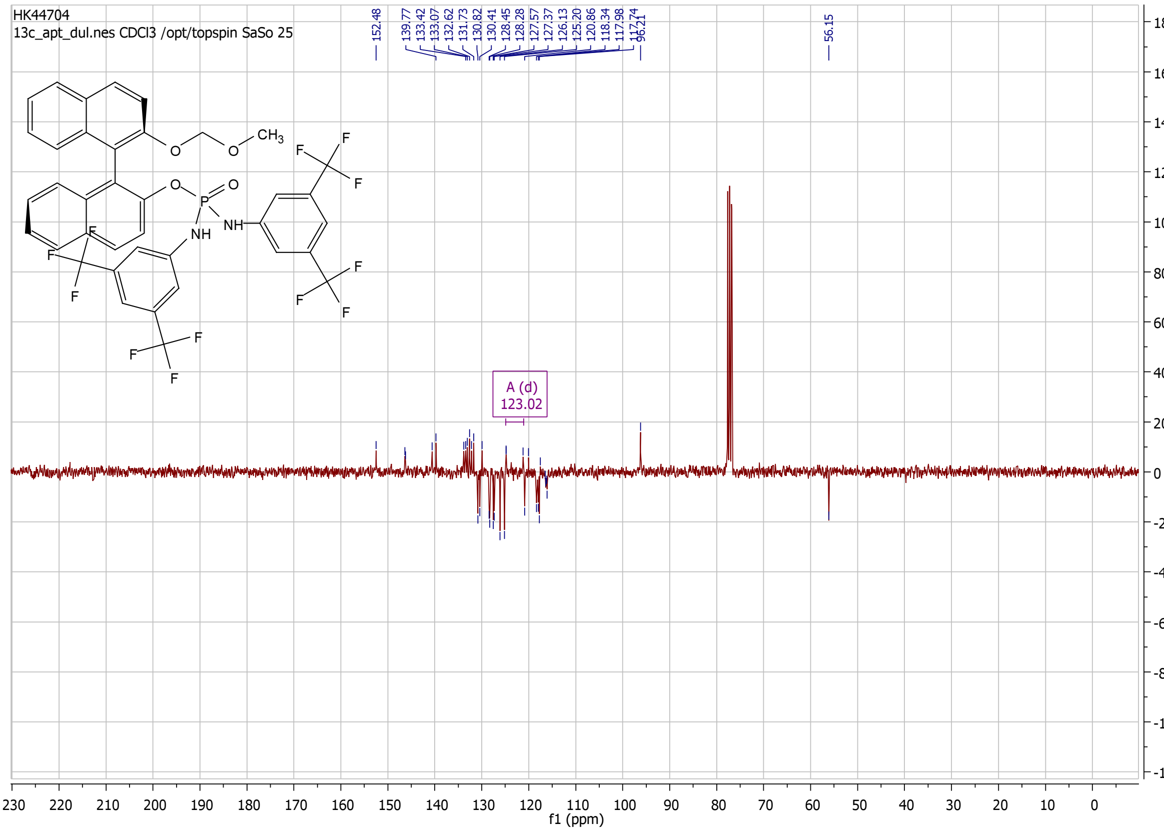
HK44704
13c_apt_dul.nes CDCl3 /opt/topspin SaSo 25
152.48
139.77
133.42
133.07
132.62
131.73
130.82
130.41
128.45
128.28
127.57
127.37
126.13
125.20
120.86
118.34
117.98
96.21
56.15
A (d)
123.02
CH3
O
NH
P
F
f1 (ppm)

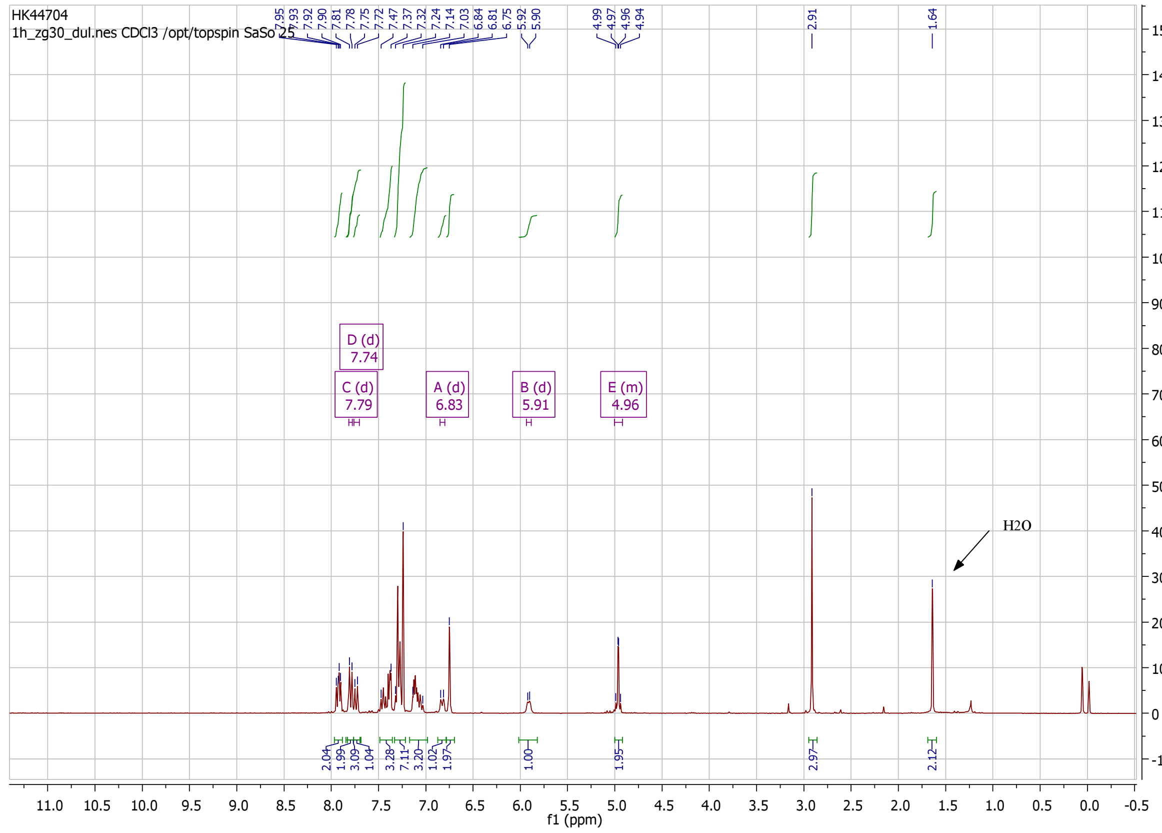
HK44704
1h_zg30_dul.nes CDCl3 /opt/topspin SaSo 25
7.95
7.93
7.92
7.90
7.81
7.78
7.75
7.72
7.47
7.37
7.32
7.24
7.14
7.03
6.84
6.81
6.75
5.92
5.90
4.99
4.97
4.96
4.94
2.91
1.64
D (d)
7.74
C (d)
7.79
A (d)
6.83
B (d)
5.91
E (m)
4.96
H2O
2.04
1.99
1.95
3.09
1.04
3.28
7.11
3.20
1.02
1.97
1.00
1.95
2.97
2.12
f1 (ppm)

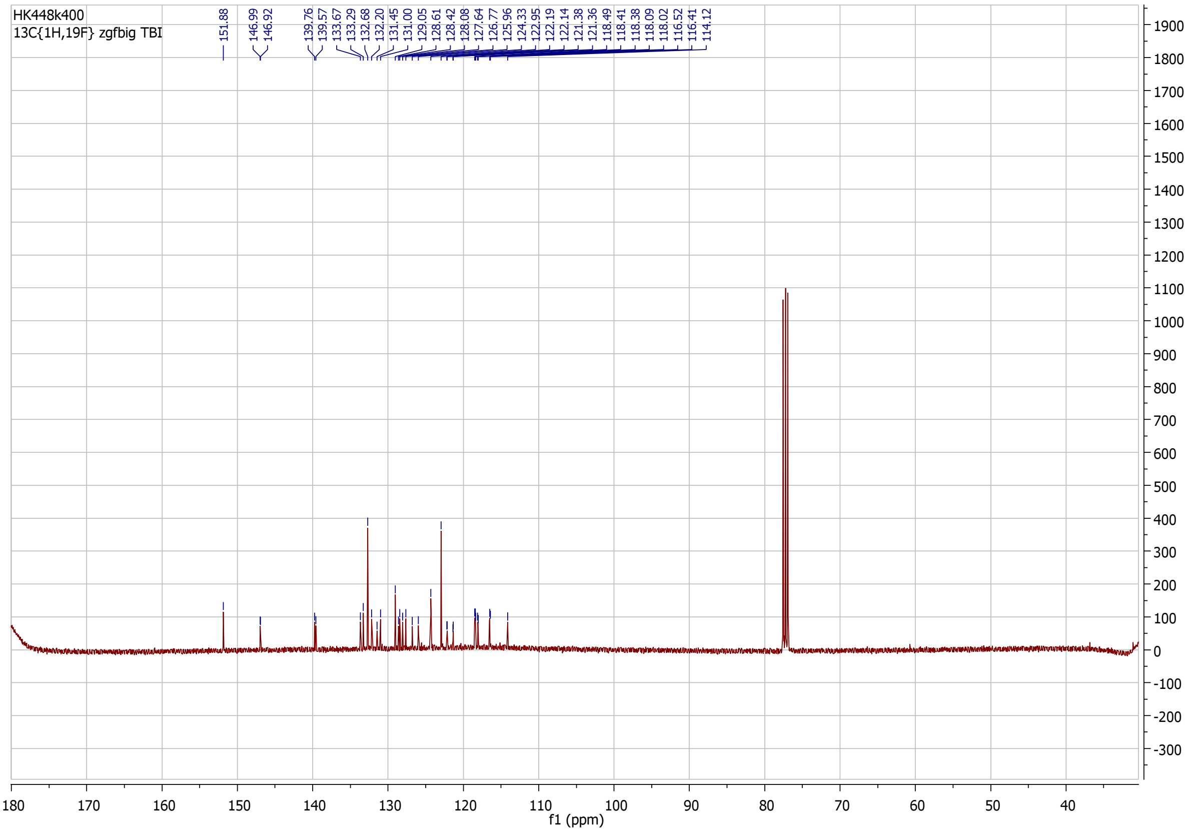

HK448k400
13C{1H,19F} zgfbig TBI
151.88
146.99
146.92
139.76
139.57
133.67
133.29
132.68
132.20
131.45
131.00
129.05
128.61
128.42
128.08
127.64
126.77
125.96
124.33
122.95
122.19
122.14
121.38
121.36
118.49
118.41
118.38
118.09
118.02
116.52
116.41
114.12
f1 (ppm)

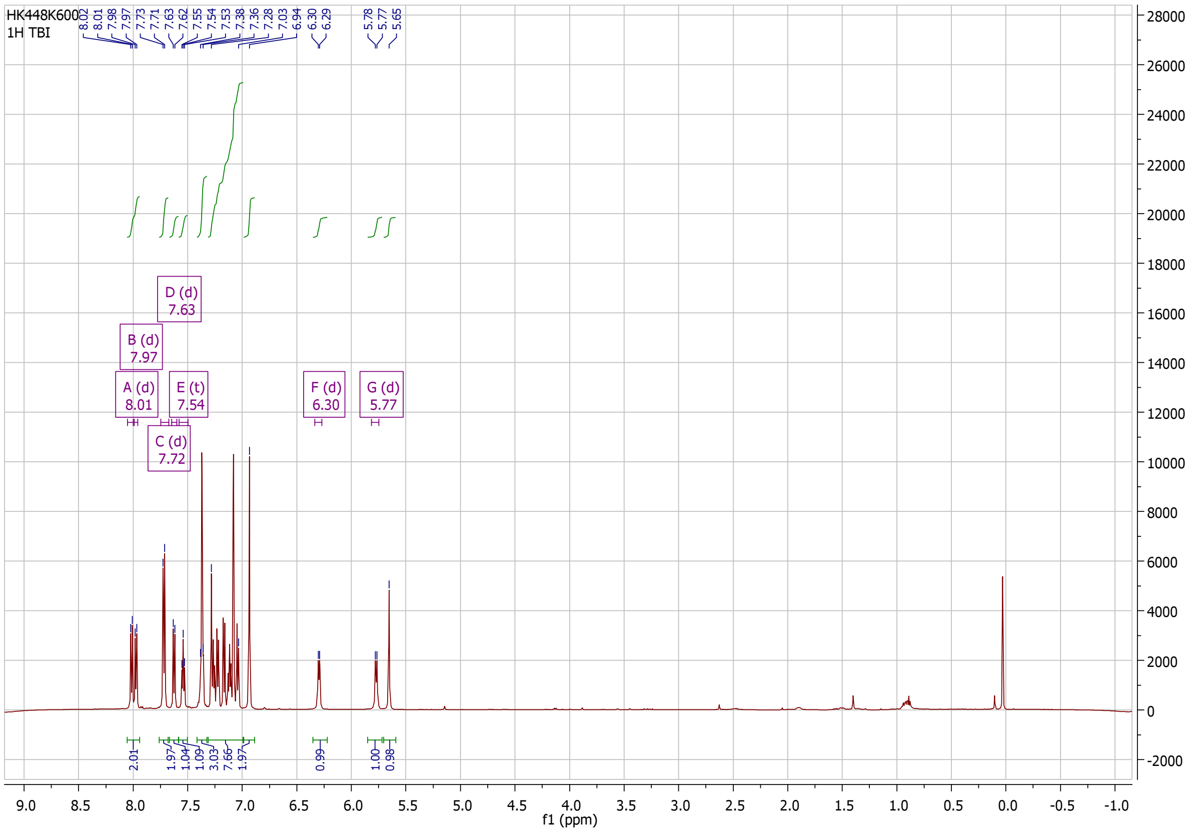

HK448K600
1H TBI
8.02
8.01
7.98
7.97
7.73
7.71
7.63
7.62
7.55
7.54
7.53
7.38
7.36
7.28
7.03
6.94
6.30
6.29
5.78
5.77
5.65
D (d) 7.63
B (d) 7.97
A (d) 8.01
E (t) 7.54
F (d) 6.30
G (d) 5.77
C (d) 7.72
2.01
1.97
1.04
1.09
3.03
7.66
1.97
0.99
1.00
0.98
f1 (ppm)

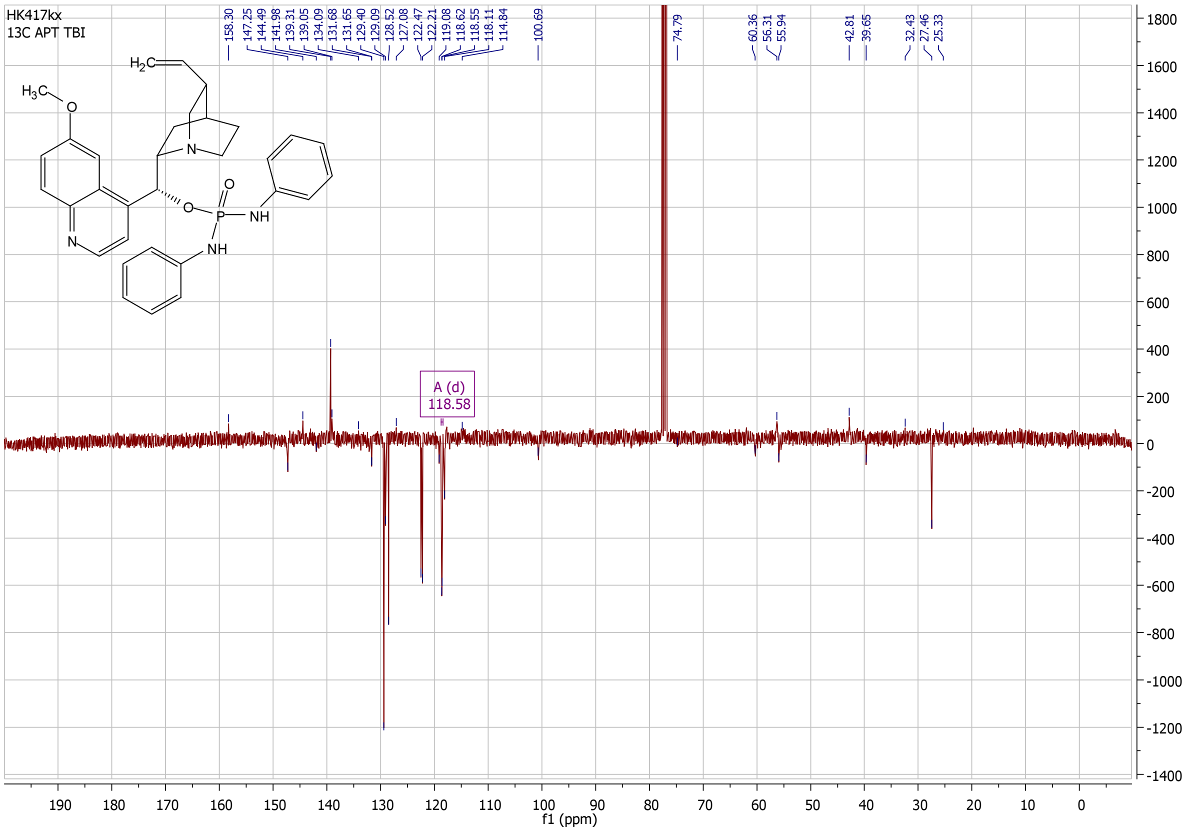
HK417kx
13C APT TBI
158.30
147.25
144.49
141.98
139.31
139.05
134.09
131.68
131.65
129.40
129.09
128.52
127.08
122.47
122.21
119.08
118.62
118.55
118.11
114.84
100.69
74.79
60.36
56.31
55.94
42.81
39.65
32.43
27.46
25.33
A (d)
118.58
f1 (ppm)

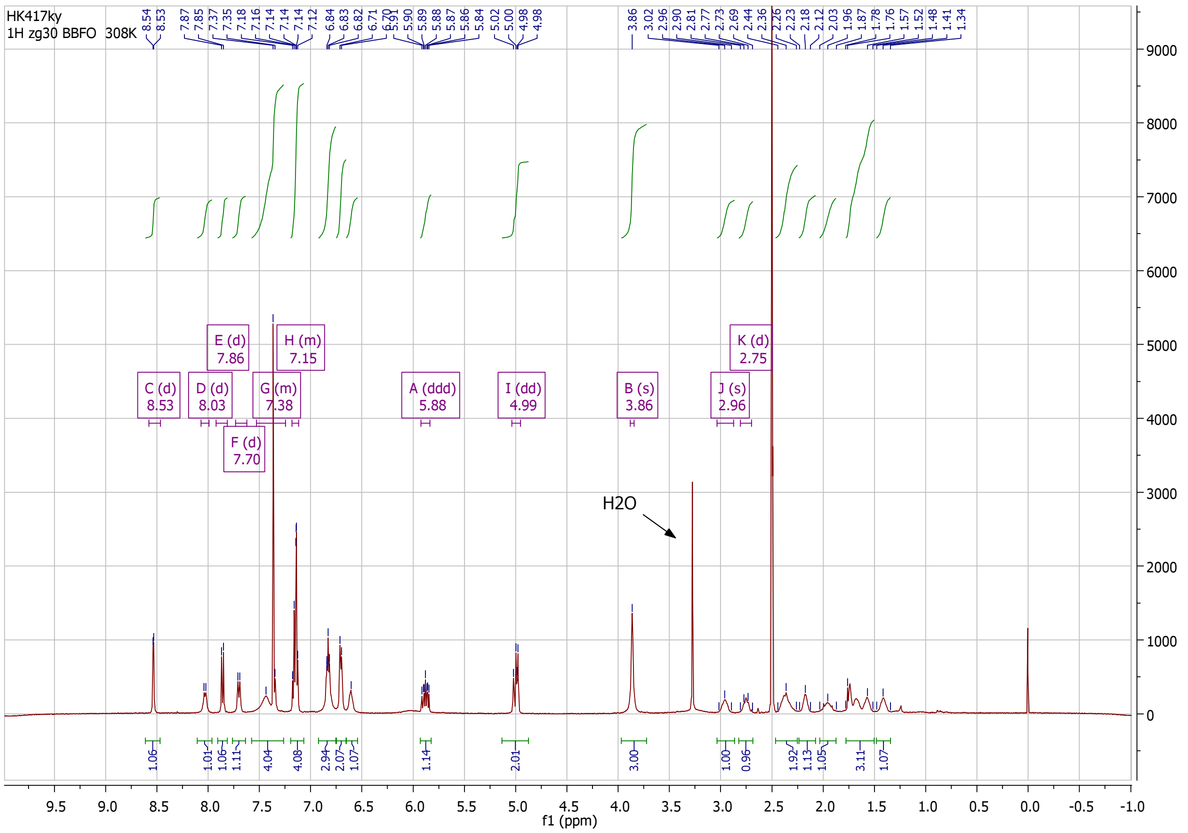
HK417ky
1H zg30 BBFO 308K
C (d)
8.53
D (d)
8.03
E (d)
7.86
F (d)
7.70
G (m)
7.38
H (m)
7.15
A (ddd)
5.88
I (dd)
4.99
B (s)
3.86
J (s)
2.96
K (d)
2.75
H2O
f1 (ppm)

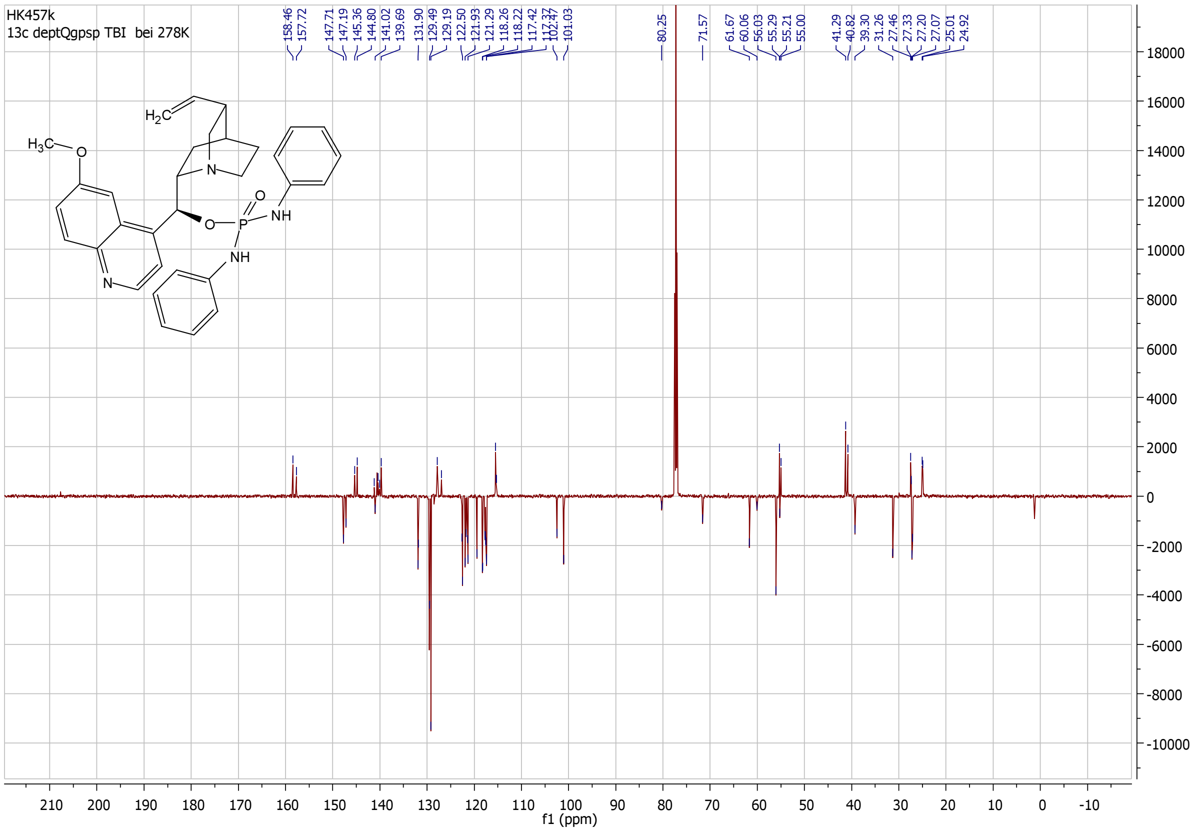
HK457k
13c deptQgpsp TBI bei 278K
f1 (ppm)

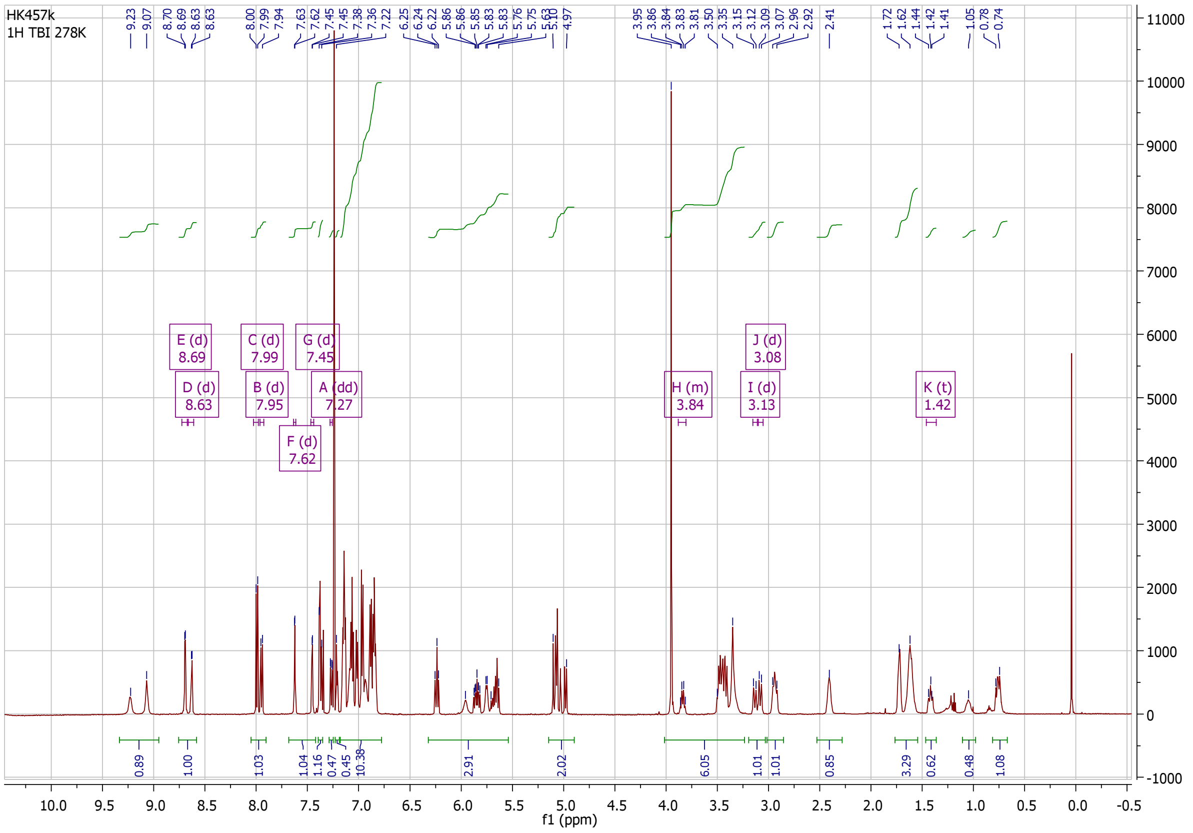
HK457k
1H TBI 278K
f1 (ppm)

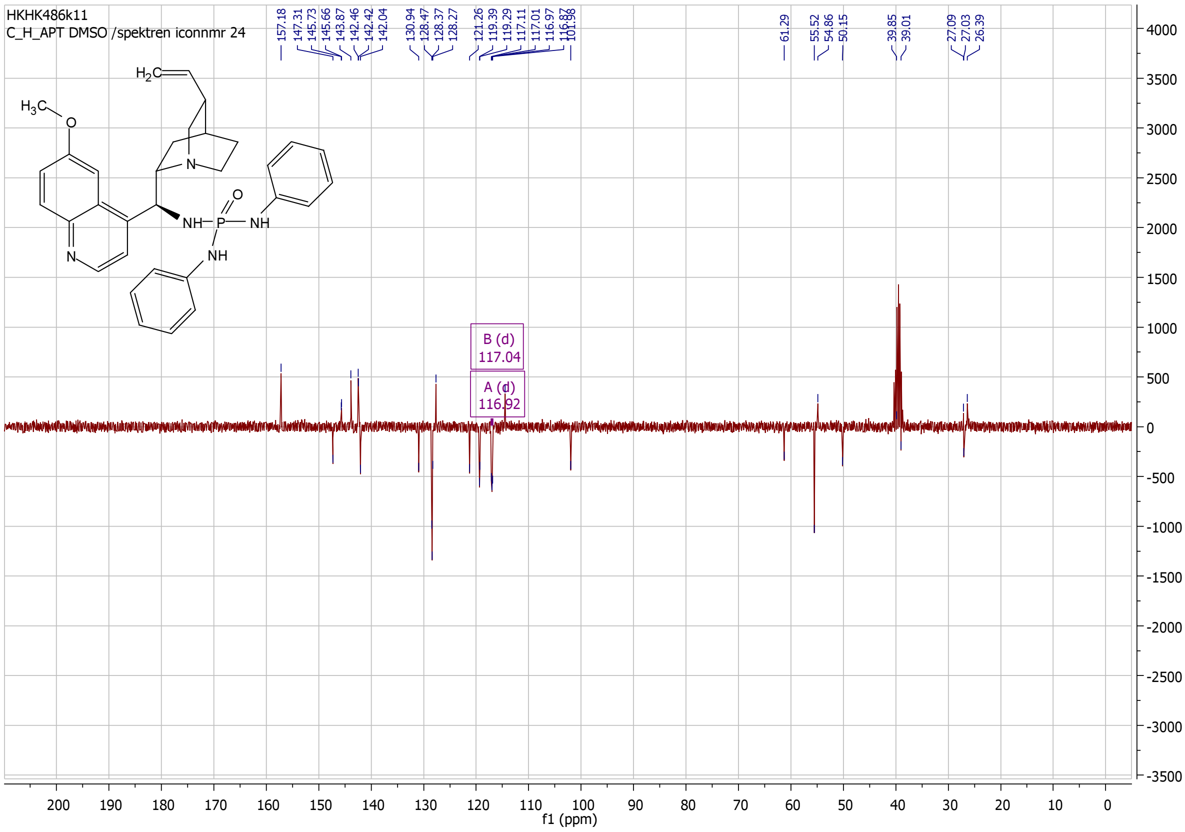
HKHK486k11
C_H_APT DMSO /spektren iconnmr 24
157.18
147.31
145.73
145.66
143.87
142.46
142.42
142.04
130.94
128.47
128.37
128.27
121.26
119.39
119.29
117.11
117.01
116.97
116.87
101.98
61.29
55.52
54.86
50.15
39.85
39.01
27.09
27.03
26.39
B (d)
117.04
A (d)
116.92
f1 (ppm)

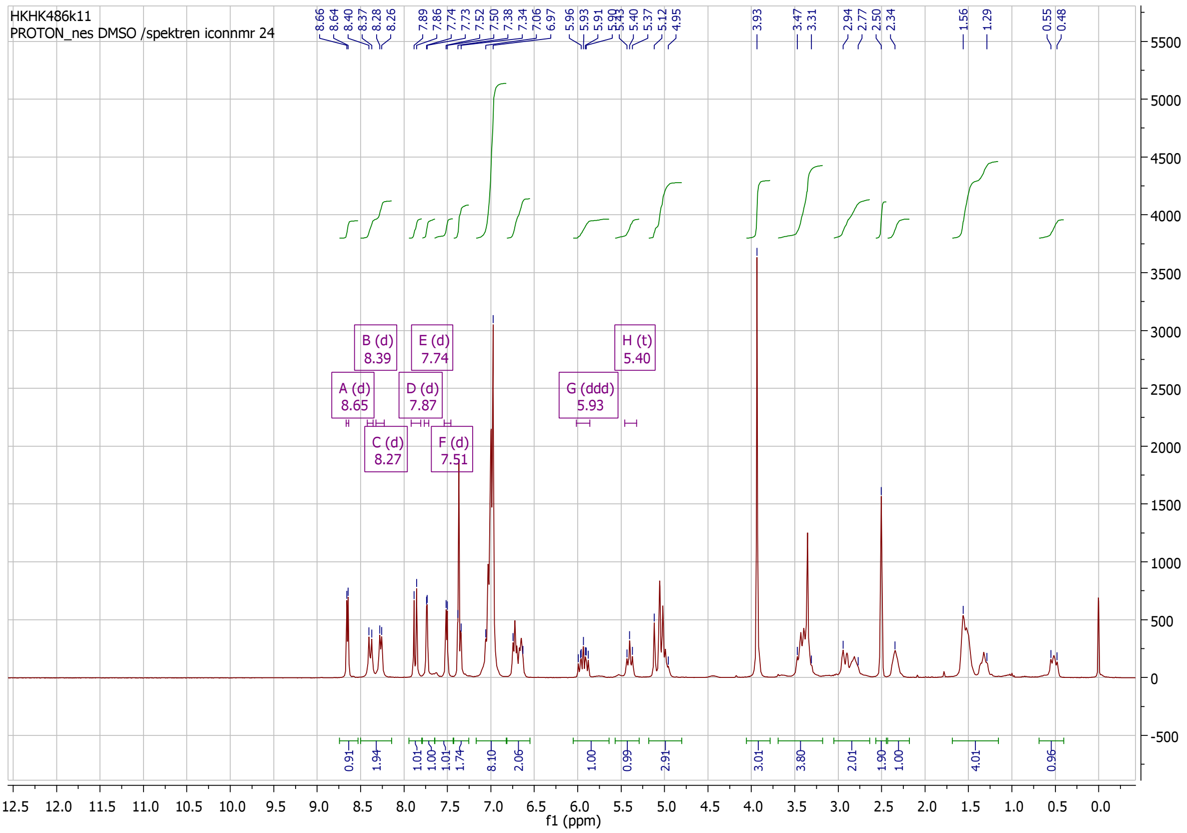
HKHK486k11
PROTON_nes DMSO /spektren iconnmr 24
A (d)
8.65
B (d)
8.39
C (d)
8.27
D (d)
7.87
E (d)
7.74
F (d)
7.51
G (ddd)
5.93
H (t)
5.40
f1 (ppm)

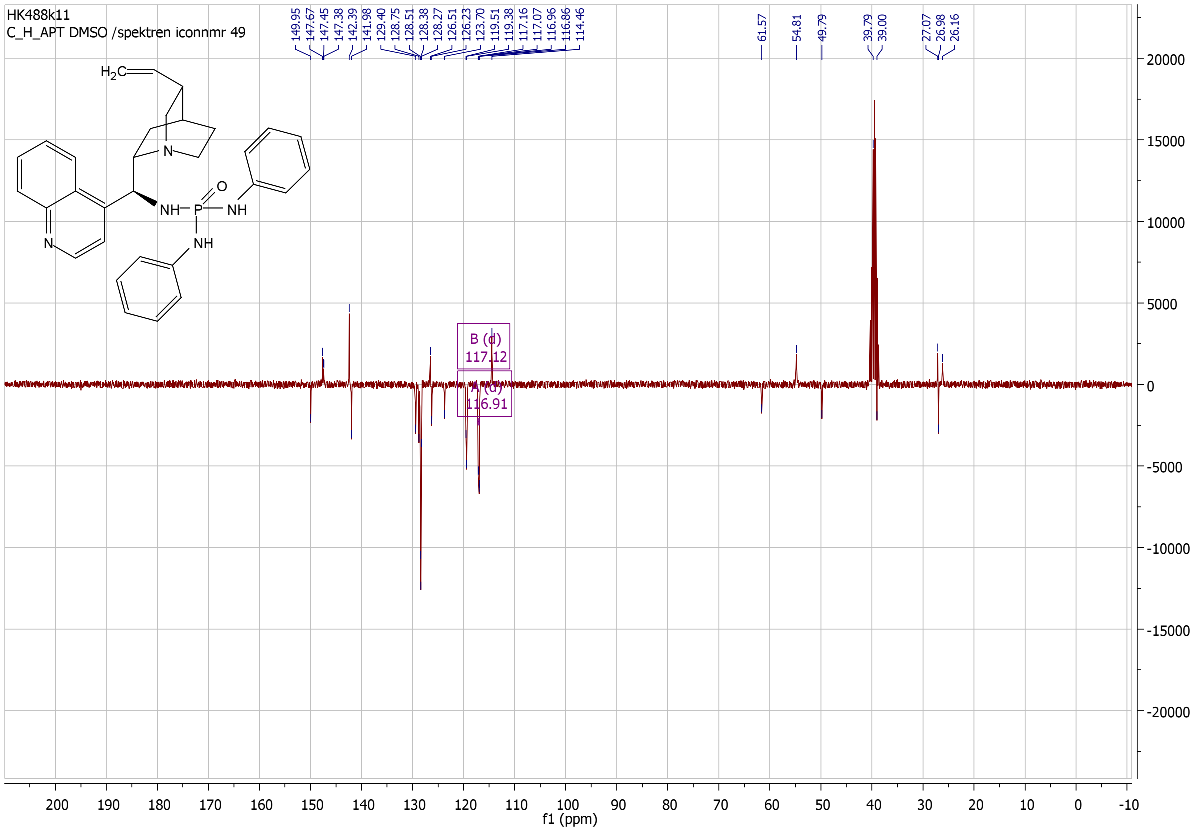
HK488k11
C_H_APT DMSO /spektren iconnmr 49
B (d) 117.12
A (d) 116.91
f1 (ppm)

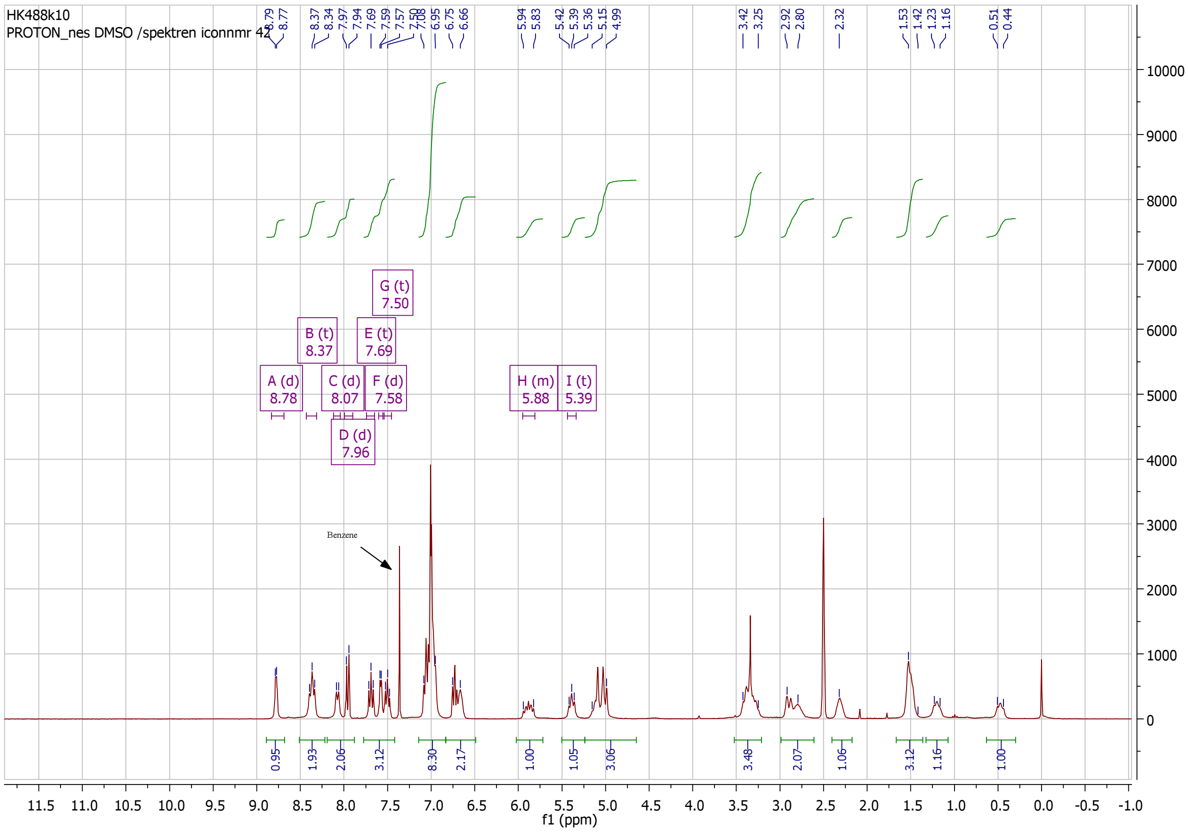
HK488k10
PROTON_nes DMSO /spektren iconnmr 42
A (d) 8.78
B (t) 8.37
C (d) 8.07
D (d) 7.96
E (t) 7.69
F (d) 7.58
G (t) 7.50
H (m) 5.88
I (t) 5.39
Benzene
f1 (ppm)

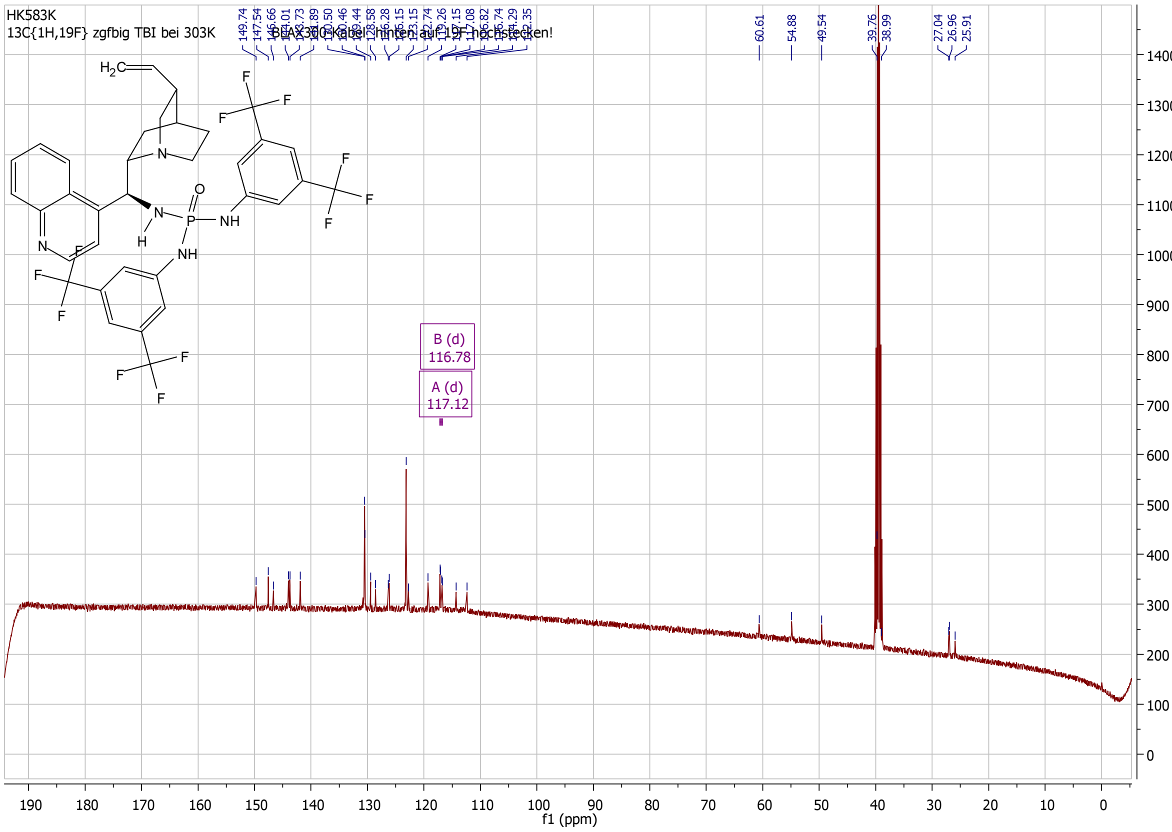
HK583K
13C{1H,19F} zgfbig TBI bei 303K
149.74
147.54
143.89
130.50
128.58
60.61
54.88
49.54
39.76
38.99
27.04
26.96
25.91
B (d)
116.78
A (d)
117.12
f1 (ppm)

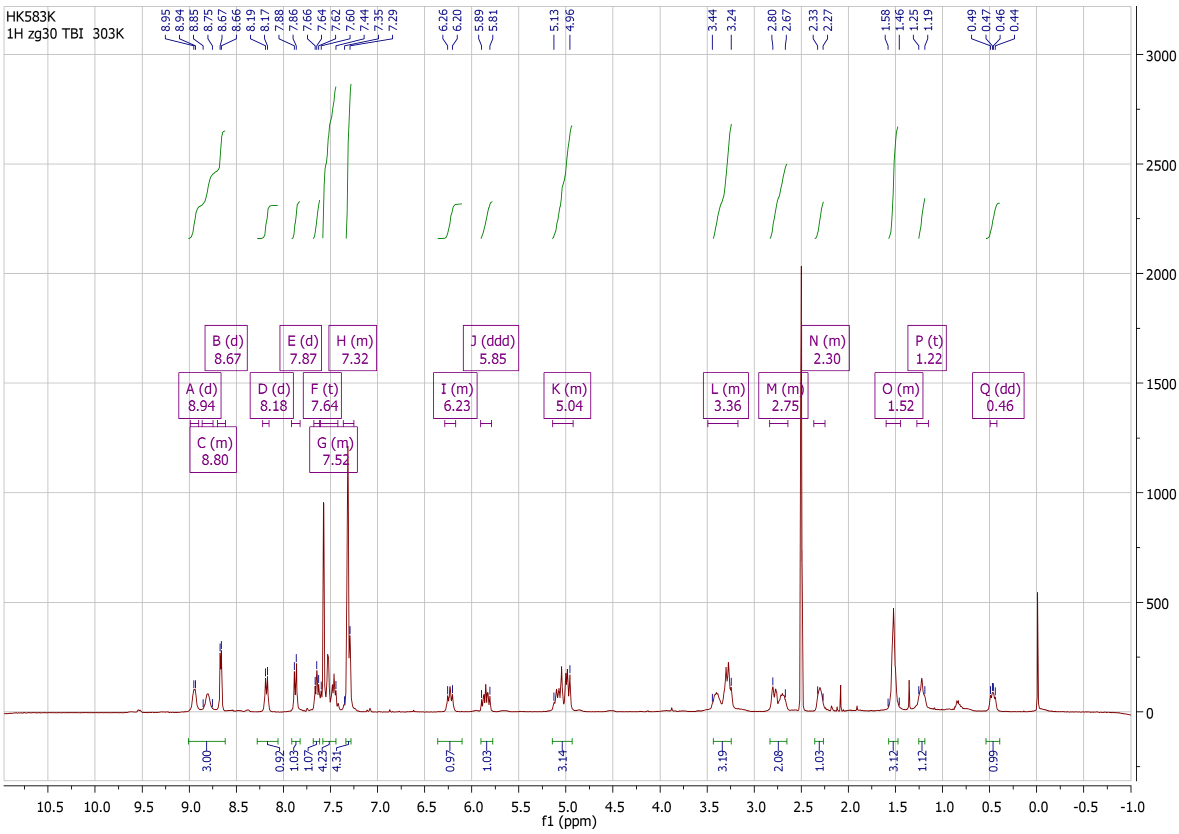
HK583K
1H zg30 TBI 303K
8.95
8.94
8.85
8.75
8.67
8.66
8.19
8.17
7.88
7.86
7.66
7.64
7.62
7.60
7.44
7.35
7.29
6.26
6.20
5.89
5.81
5.13
4.96
3.44
3.24
2.80
2.67
2.33
2.27
1.58
1.46
1.25
1.19
0.49
0.47
0.46
0.44
A (d)
8.94
B (d)
8.67
C (m)
8.80
D (d)
8.18
E (d)
7.87
F (t)
7.64
G (m)
7.52
H (m)
7.32
I (m)
6.23
J (ddd)
5.85
K (m)
5.04
L (m)
3.36
M (m)
2.75
N (m)
2.30
O (m)
1.52
P (t)
1.22
Q (dd)
0.46
3.00
0.92
1.03
1.07
4.23
4.31
0.97
1.03
3.14
3.19
2.08
1.03
3.12
1.12
0.99
f1 (ppm)

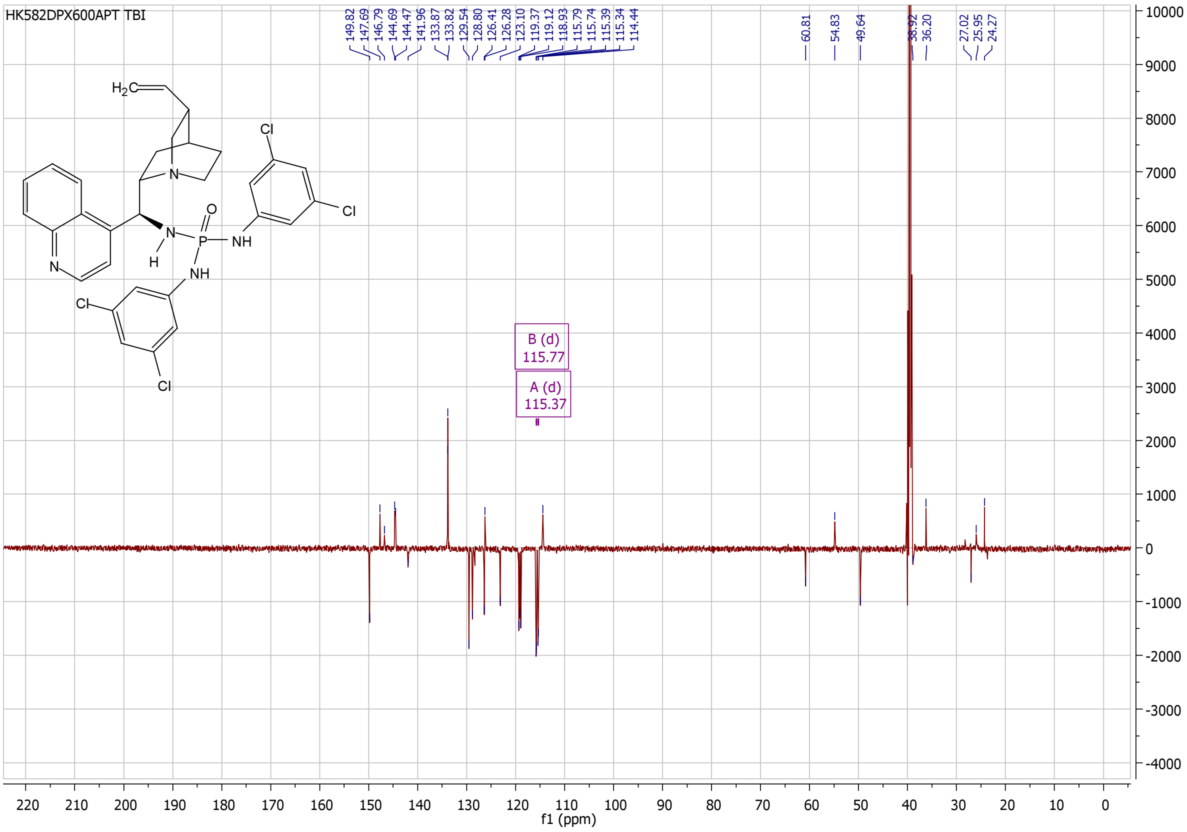
HK582DPX600APT TBI
149.82
147.69
146.79
144.69
144.47
141.96
133.87
133.82
129.54
128.80
126.41
126.28
123.10
119.37
119.12
118.93
115.79
115.74
115.39
115.34
114.44
60.81
54.83
49.64
38.92
36.20
27.02
25.95
24.27
B (d)
115.77
A (d)
115.37
f1 (ppm)

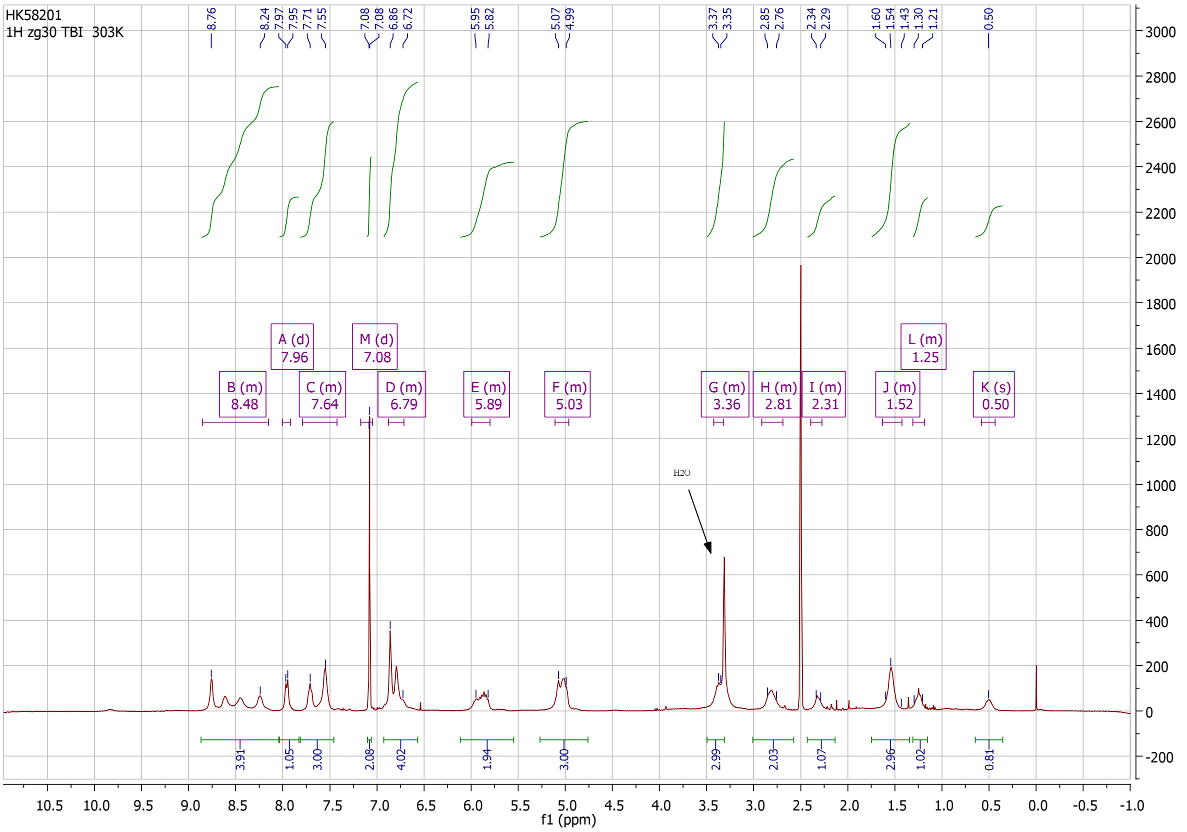
HK58201
1H zg30 TBI 303K
8.76
8.24
7.97
7.95
7.71
7.55
7.08
7.08
6.86
6.72
5.95
5.82
5.07
4.99
3.37
3.35
2.85
2.76
2.34
2.29
1.60
1.54
1.43
1.30
1.21
0.50
A (d)
7.96
M (d)
7.08
L (m)
1.25
B (m)
8.48
C (m)
7.64
D (m)
6.79
E (m)
5.89
F (m)
5.03
G (m)
3.36
H (m)
2.81
I (m)
2.31
J (m)
1.52
K (s)
0.50
H2O
3.91
1.05
3.00
2.08
4.02
1.94
3.00
2.99
2.03
1.07
2.96
1.02
0.81
f1 (ppm)

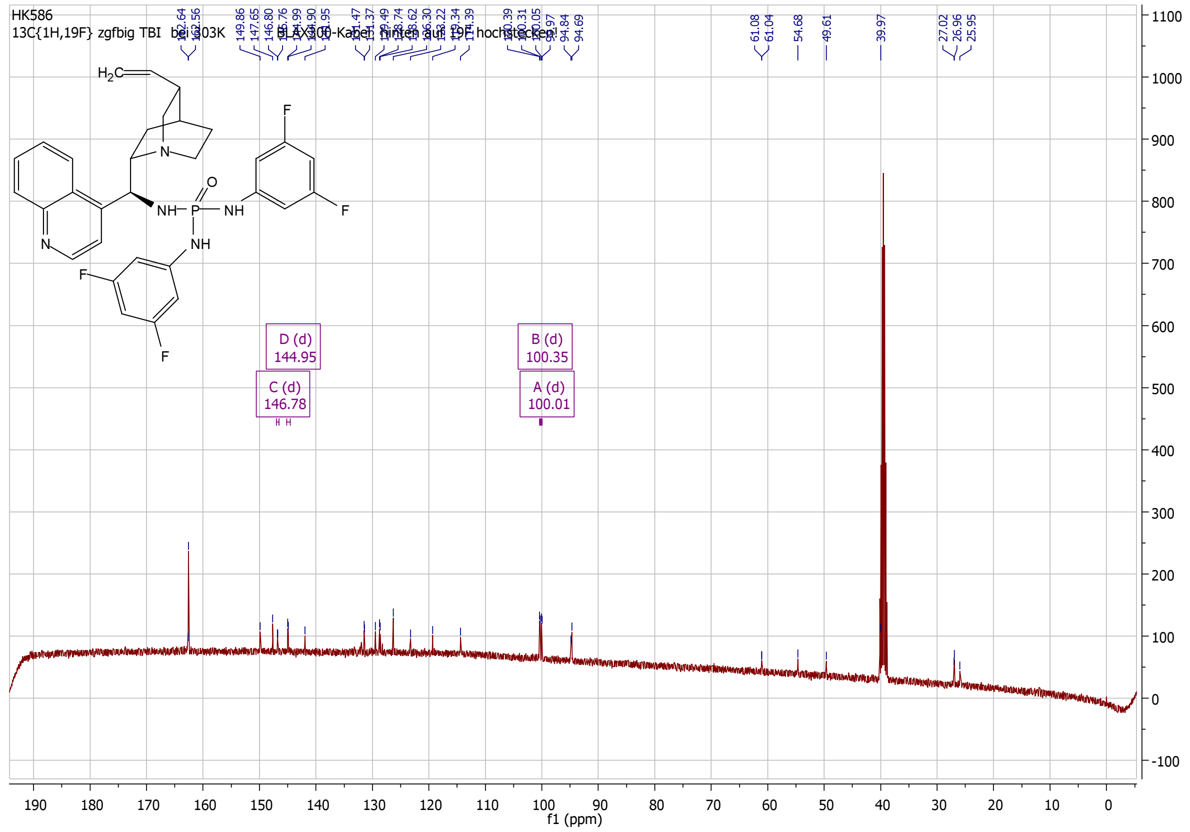
HK586
13C{1H,19F} zgfbig TBI bei 303K
149.86
147.65
146.80
61.08
61.04
54.68
49.61
39.97
27.02
26.96
25.95
94.84
94.69
D (d)
144.95
C (d)
146.78
B (d)
100.35
A (d)
100.01
f1 (ppm)

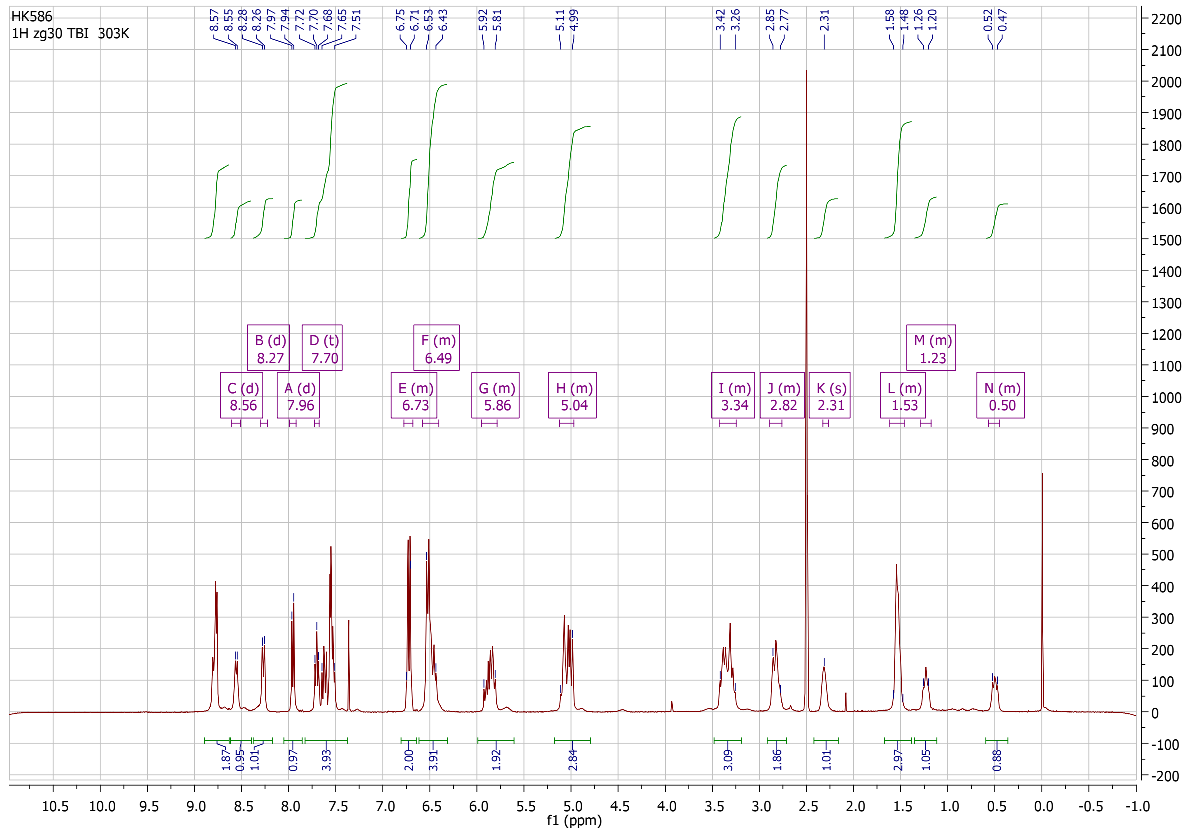
HK586
1H zg30 TBI 303K
8.57
8.55
8.28
8.26
7.97
7.94
7.72
7.70
7.68
7.65
7.51
6.75
6.71
6.53
6.43
5.92
5.81
5.11
4.99
3.42
3.26
2.85
2.77
2.31
1.58
1.48
1.26
1.20
0.52
0.47
C (d)
8.56
B (d)
8.27
A (d)
7.96
D (t)
7.70
E (m)
6.73
F (m)
6.49
G (m)
5.86
H (m)
5.04
I (m)
3.34
J (m)
2.82
K (s)
2.31
L (m)
1.53
M (m)
1.23
N (m)
0.50
1.87
0.95
1.01
0.97
3.93
2.00
3.91
1.92
2.84
3.09
1.86
1.01
2.97
1.05
0.88
f1 (ppm)

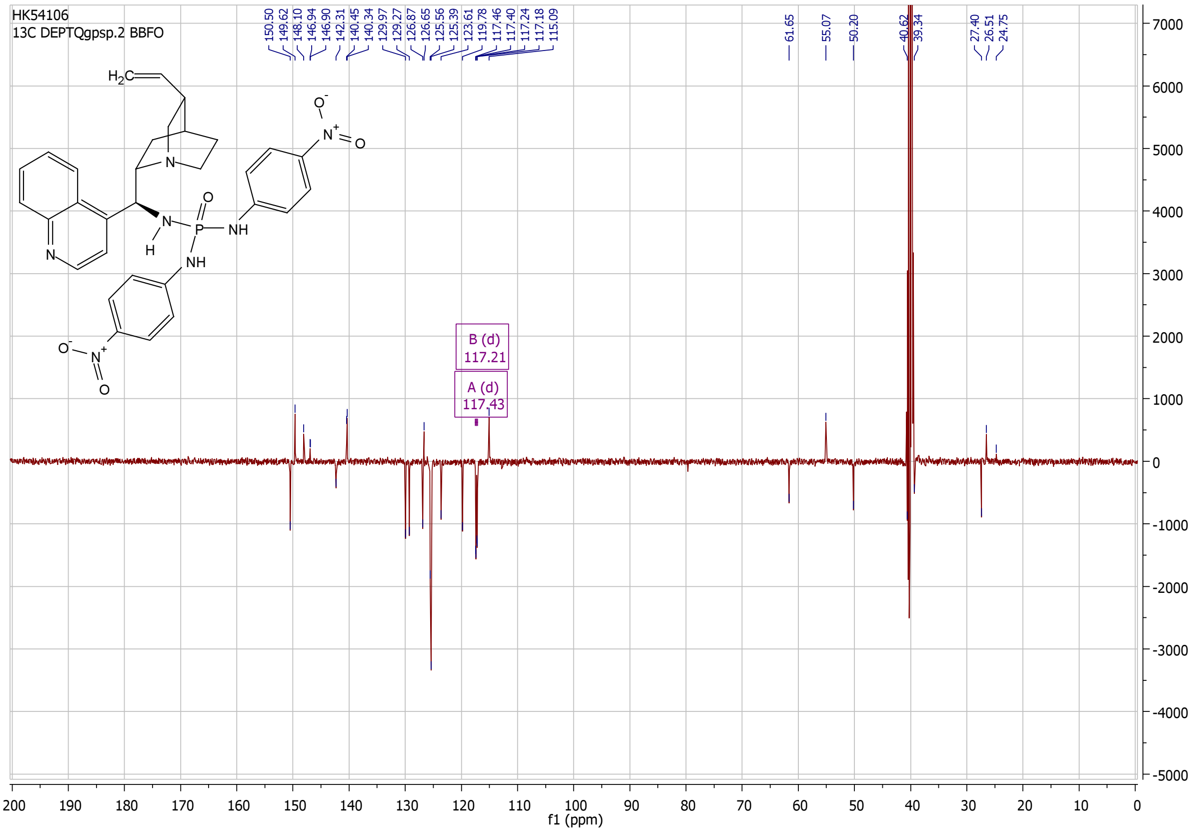
HK54106
13C DEPTQgpsp.2 BBFO
150.50 149.62 148.10 146.94 146.90 142.31 140.45 140.34 129.97 129.27 126.87 126.65 125.56 125.39 123.61 119.78 117.46 117.40 117.24 117.18 115.09
61.65 55.07 50.20 40.62 39.34 27.40 26.51 24.75
B (d) 117.21
A (d) 117.43
f1 (ppm)

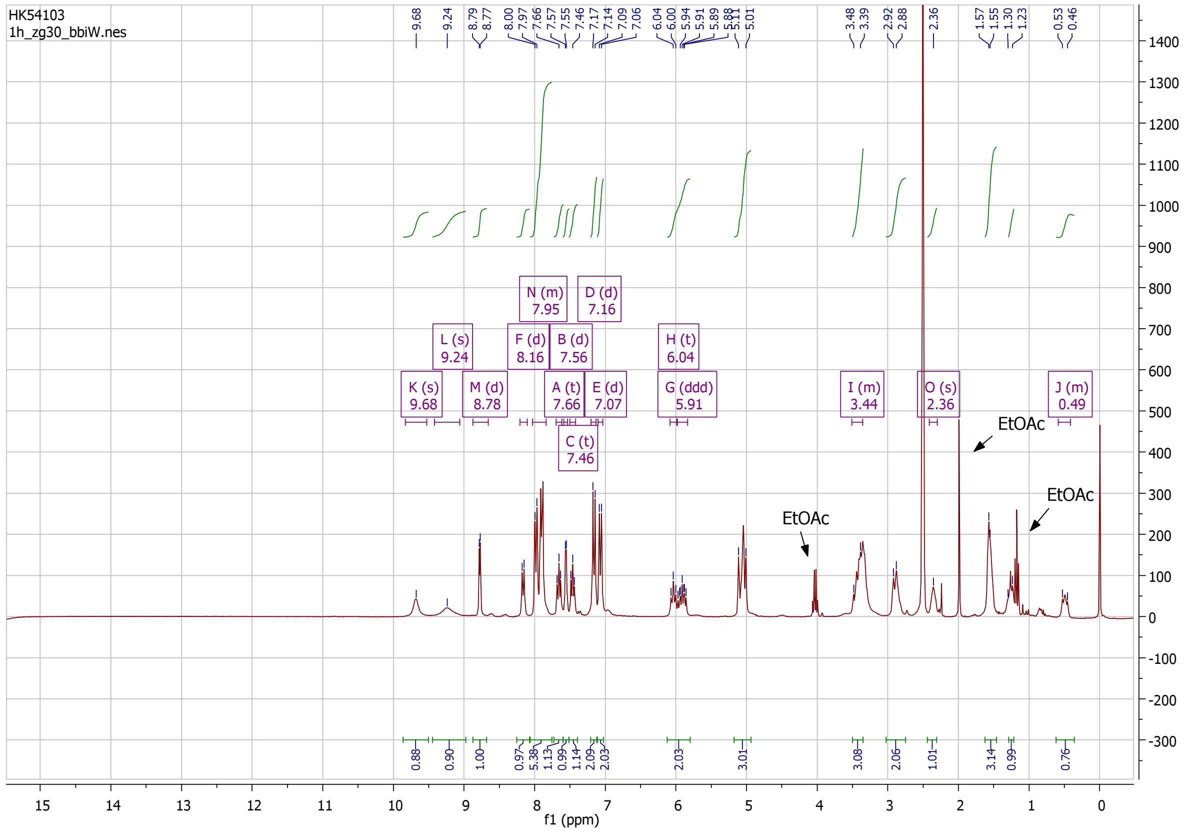
HK54103
1h_zg30_bbiW.nes
9.68 9.24 8.79 8.77 8.00 7.97 7.66 7.57 7.55 7.46 7.17 7.14 7.09 7.06 6.04 6.00 5.94 5.91 5.89 5.88 5.11 5.01
3.48 3.39 2.92 2.88 2.36 1.57 1.55 1.30 1.23 0.53 0.46
N (m) 7.95
D (d) 7.16
L (s) 9.24
F (d) 8.16
B (d) 7.56
H (t) 6.04
K (s) 9.68
M (d) 8.78
A (t) 7.66
E (d) 7.07
G (ddd) 5.91
I (m) 3.44
O (s) 2.36
J (m) 0.49
C (t) 7.46
EtOAc
EtOAc
EtOAc
0.88 0.90 1.00 0.97 5.38 1.13 0.99 1.14 2.09 2.03 2.03 3.01 3.08 2.06 1.01 3.14 0.99 0.76
f1 (ppm)

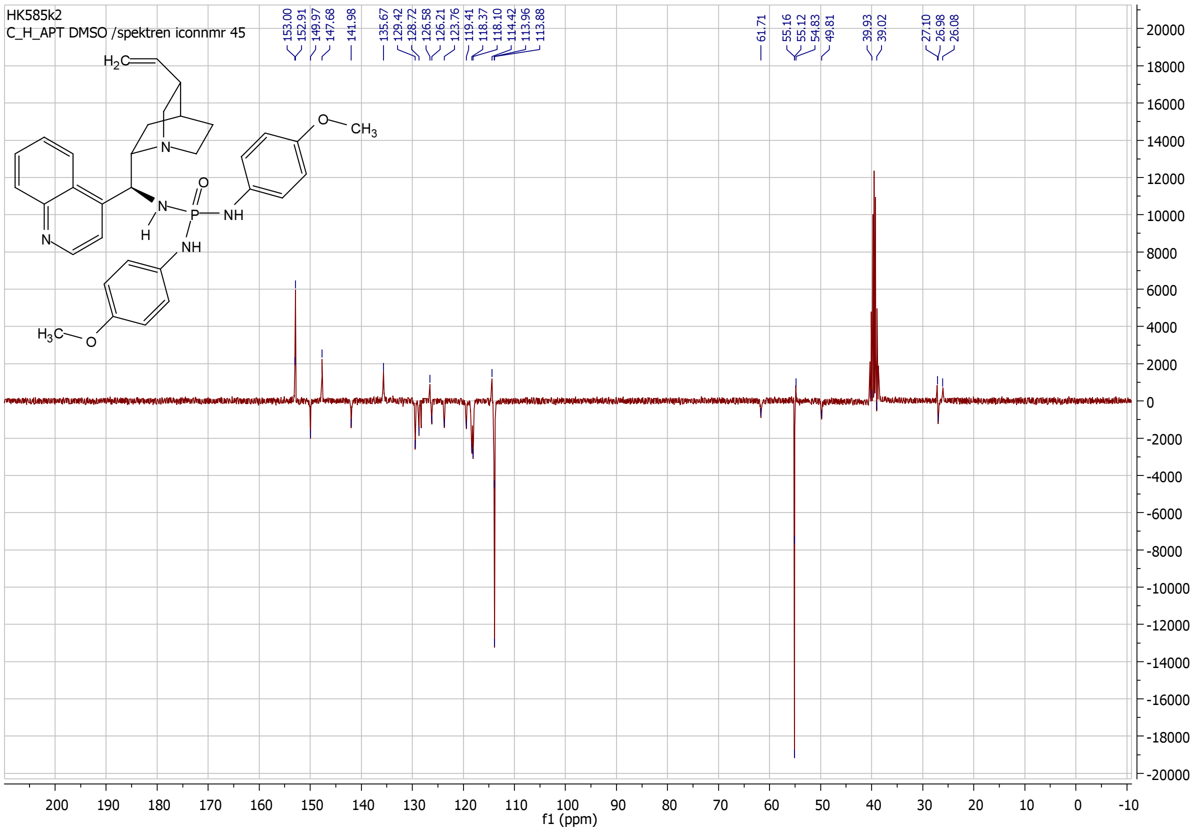
HK585k2
C_H_APT DMSO /spektren iconnmr 45
153.00 152.91 149.97 147.68 141.98 135.67 129.42 128.72 126.58 126.21 123.76 119.41 118.37 118.10 114.42 113.96 113.88
61.71 55.16 55.12 54.83 49.81 39.93 39.02 27.10 26.98 26.08
f1 (ppm)

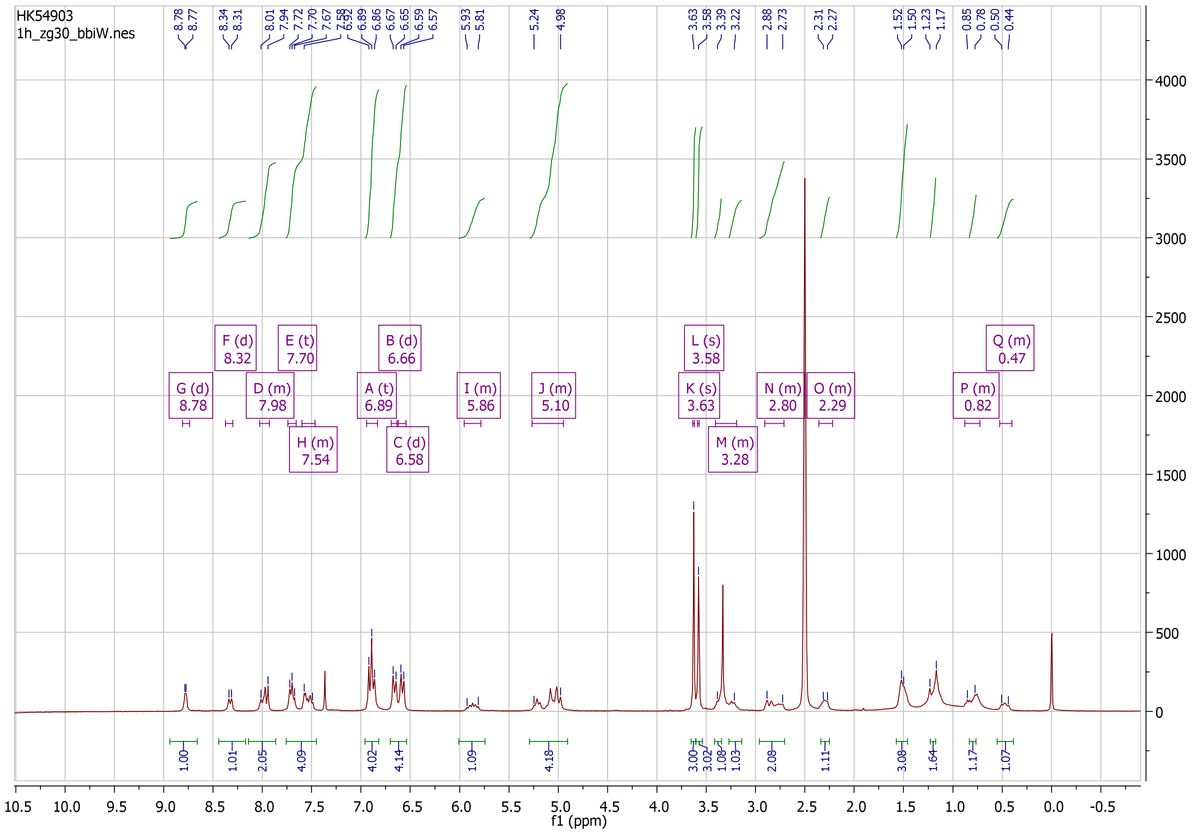
HK54903
1h_zg30_bbiW.nes
G (d) 8.78
F (d) 8.32
D (m) 7.98
E (t) 7.70
H (m) 7.54
A (t) 6.89
B (d) 6.66
C (d) 6.58
I (m) 5.86
J (m) 5.10
K (s) 3.63
L (s) 3.58
M (m) 3.28
N (m) 2.80
O (m) 2.29
P (m) 0.82
Q (m) 0.47
f1 (ppm)

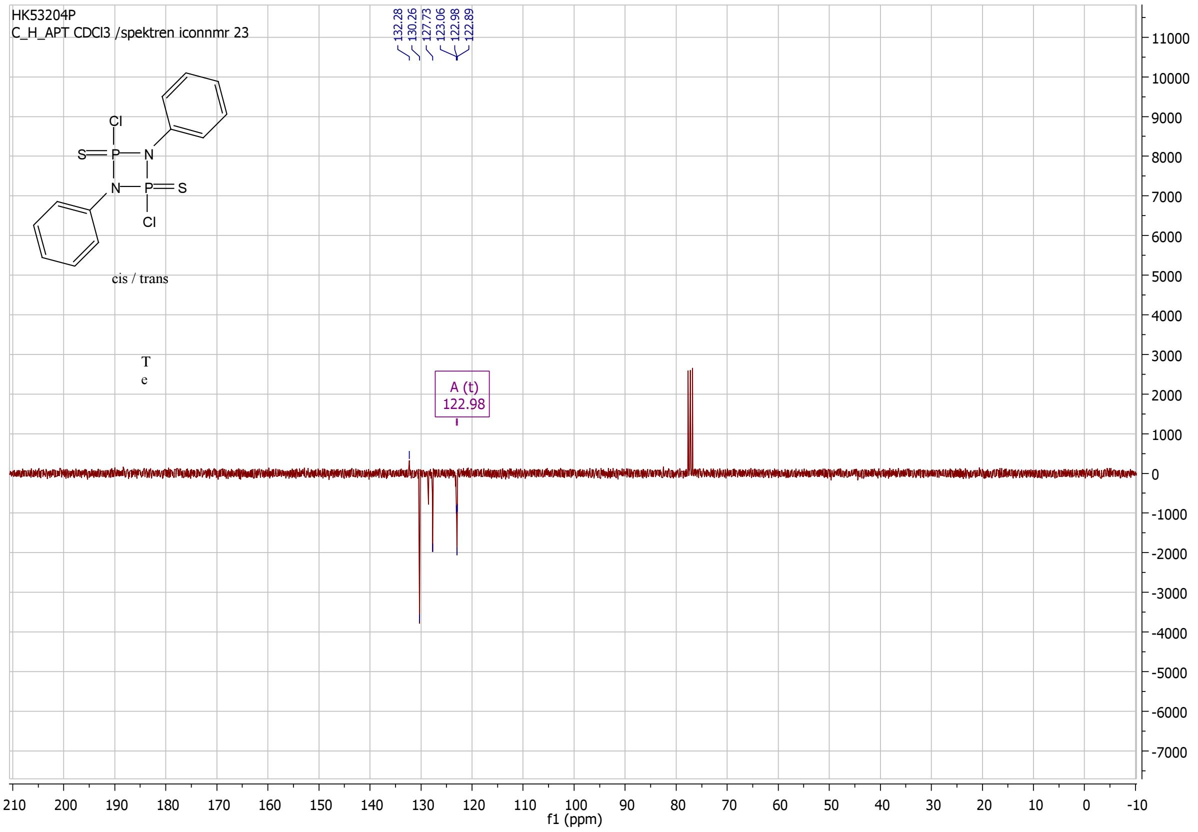
HK53204P
C_H_APT CDCl3 /spektren iconnmr 23
132.28
130.26
127.73
123.06
122.98
122.89
Cl
S
P
N
N
P
S
Cl
cis / trans
A (t)
122.98
f1 (ppm)

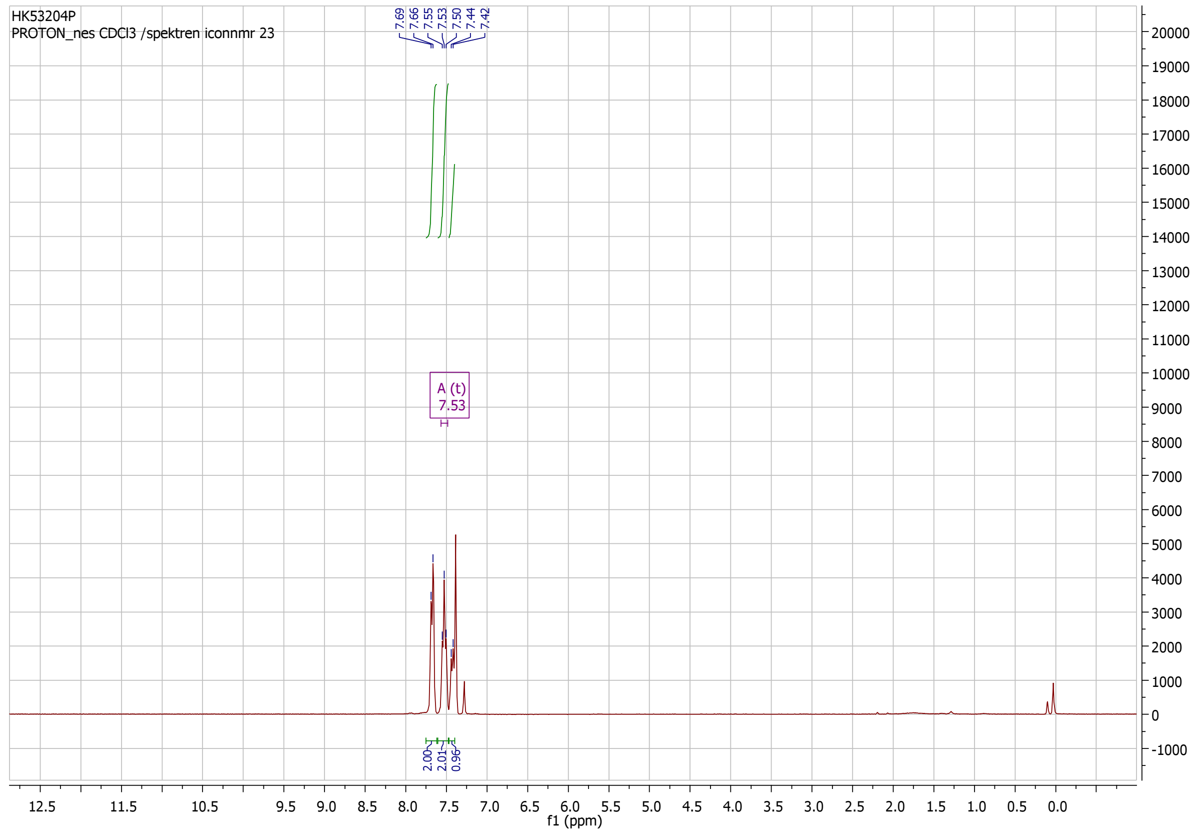
HK53204P
PROTON_nes CDCl3 /spektren iconnmr 23
7.69
7.66
7.55
7.53
7.50
7.44
7.42
A (t)
7.53
2.00
2.01
0.96
f1 (ppm)

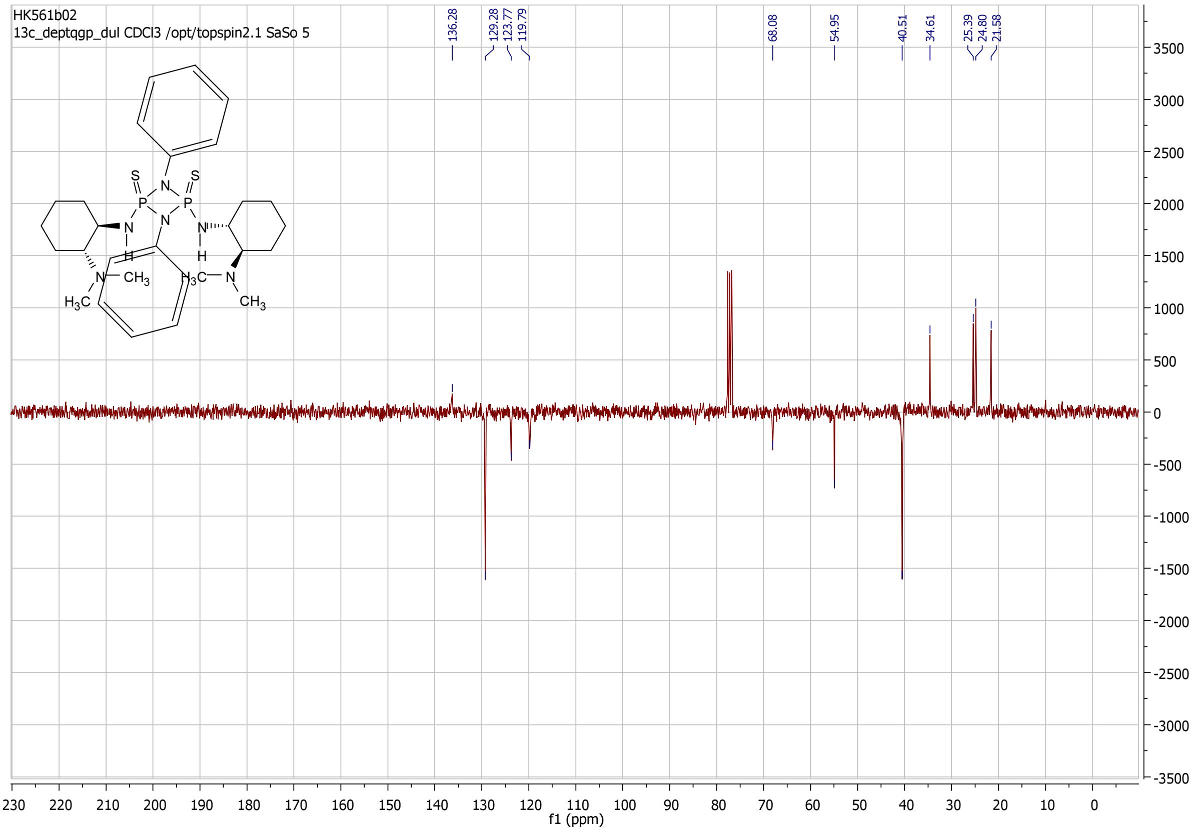
HK561b02
13c_deptqgp_dul CDCl3 /opt/topspin2.1 SaSo 5
136.28
129.28
123.77
119.79
68.08
54.95
40.51
34.61
25.39
24.80
21.58
f1 (ppm)

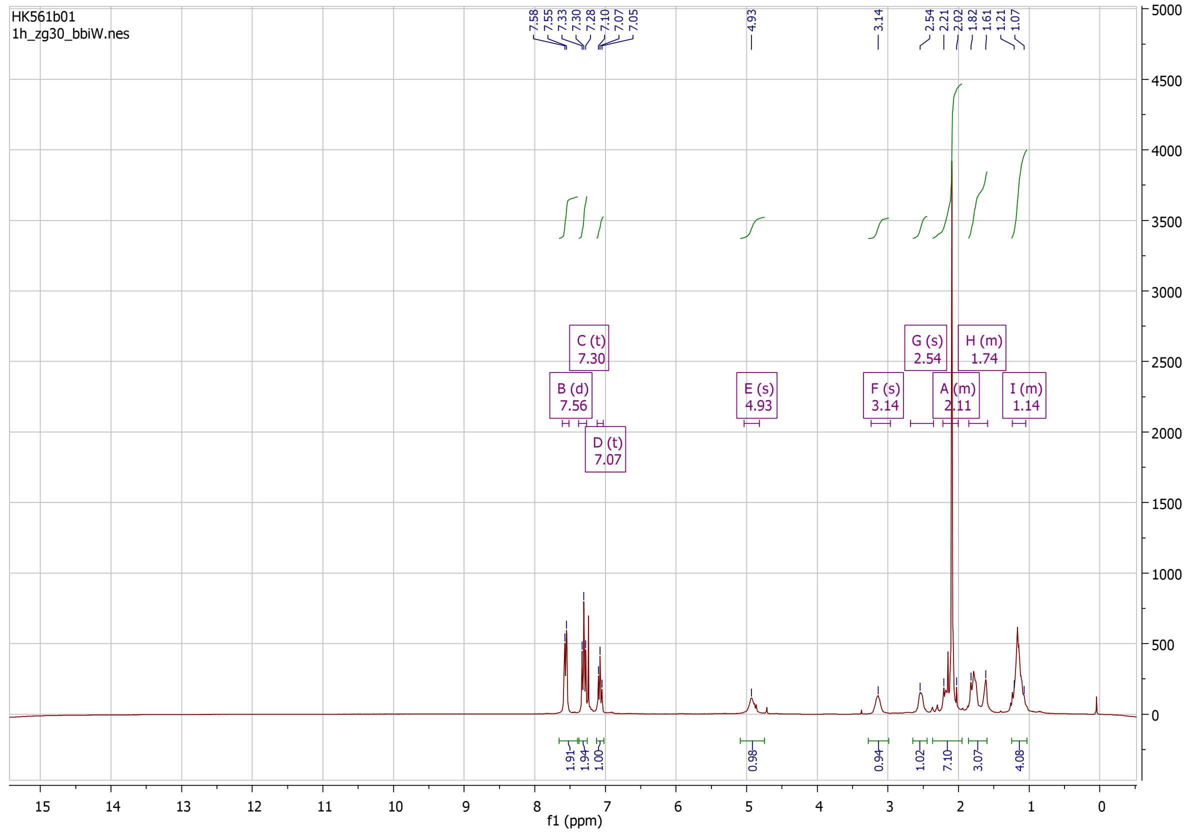
HK561b01
1h_zg30_bbiW.nes
7.58
7.55
7.33
7.30
7.28
7.10
7.07
7.05
4.93
3.14
2.54
2.21
2.02
1.82
1.61
1.21
1.07
C (t) 7.30
B (d) 7.56
D (t) 7.07
E (s) 4.93
F (s) 3.14
G (s) 2.54
A (m) 2.11
H (m) 1.74
I (m) 1.14
1.91
1.94
1.00
0.98
0.94
1.02
7.10
3.07
4.08
f1 (ppm)

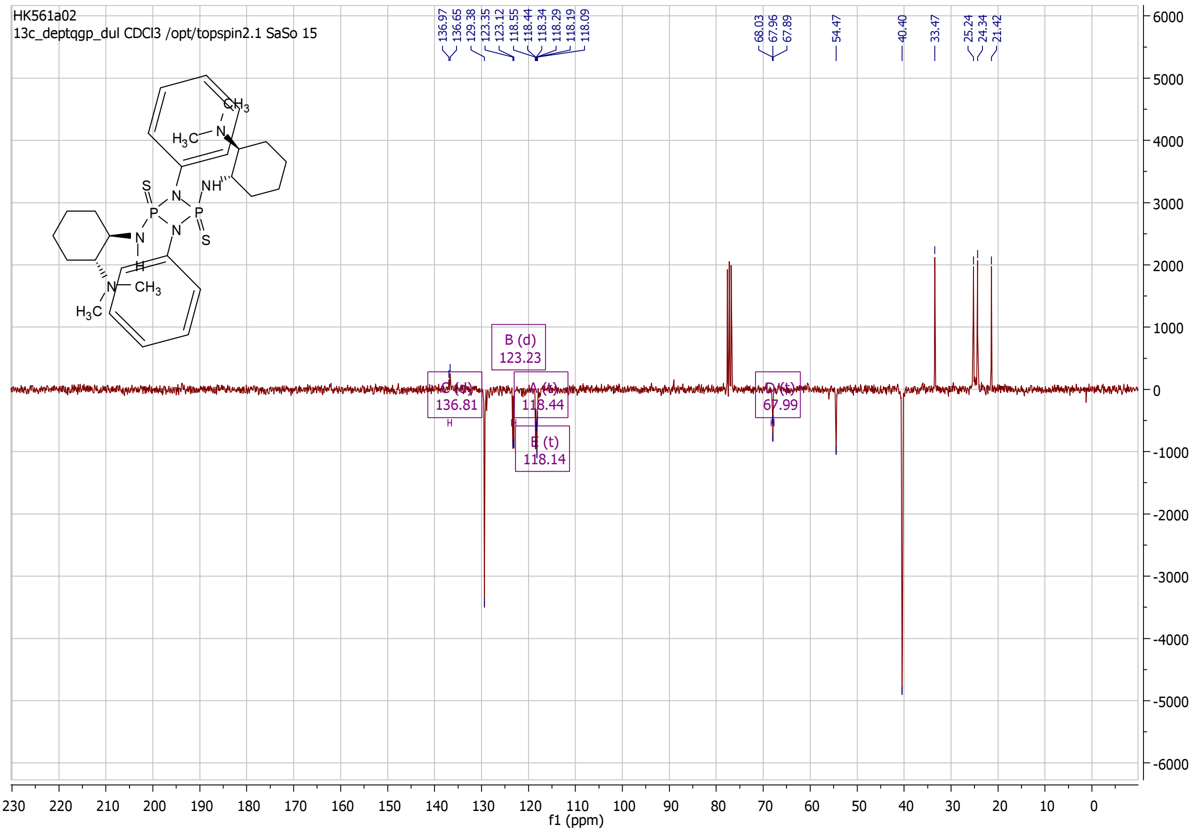
HK561a02
13c_deptqgp_dul CDCl3 /opt/topspin2.1 SaSo 15
136.97
136.65
129.38
123.35
123.12
118.55
118.44
118.34
118.29
118.19
118.09
68.03
67.96
67.89
54.47
40.40
33.47
25.24
24.34
21.42
B (d)
123.23
C (d)
136.81
A (t)
118.44
E (t)
118.14
D (t)
67.99
f1 (ppm)

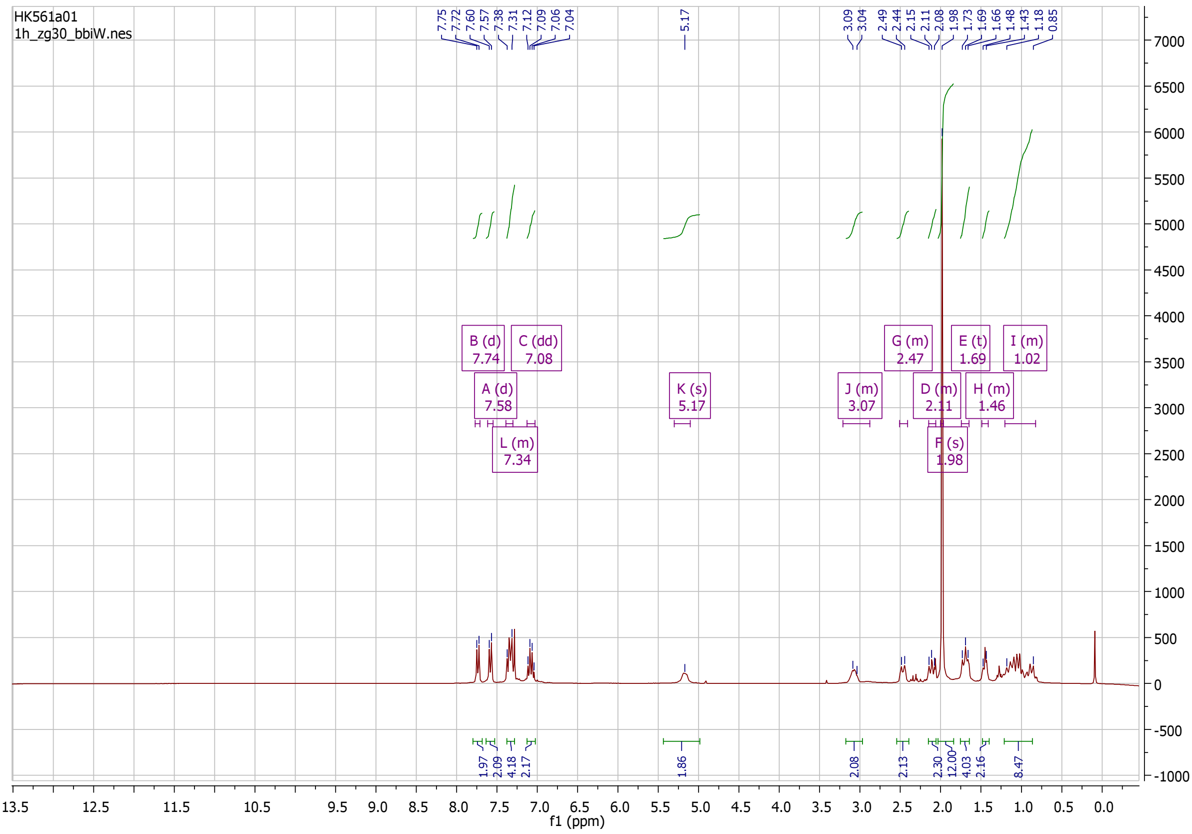
HK561a01
1h_zg30_bbiW.nes
7.75
7.72
7.60
7.57
7.38
7.31
7.12
7.09
7.06
7.04
5.17
3.09
3.04
2.49
2.44
2.15
2.11
2.08
1.98
1.73
1.69
1.66
1.48
1.43
1.18
0.85
B (d)
7.74
C (dd)
7.08
A (d)
7.58
L (m)
7.34
K (s)
5.17
J (m)
3.07
G (m)
2.47
D (m)
2.11
F (s)
1.98
E (t)
1.69
H (m)
1.46
I (m)
1.02
1.97
2.09
4.18
2.17
1.86
2.08
2.13
2.30
12.00
4.03
2.16
8.47
f1 (ppm)

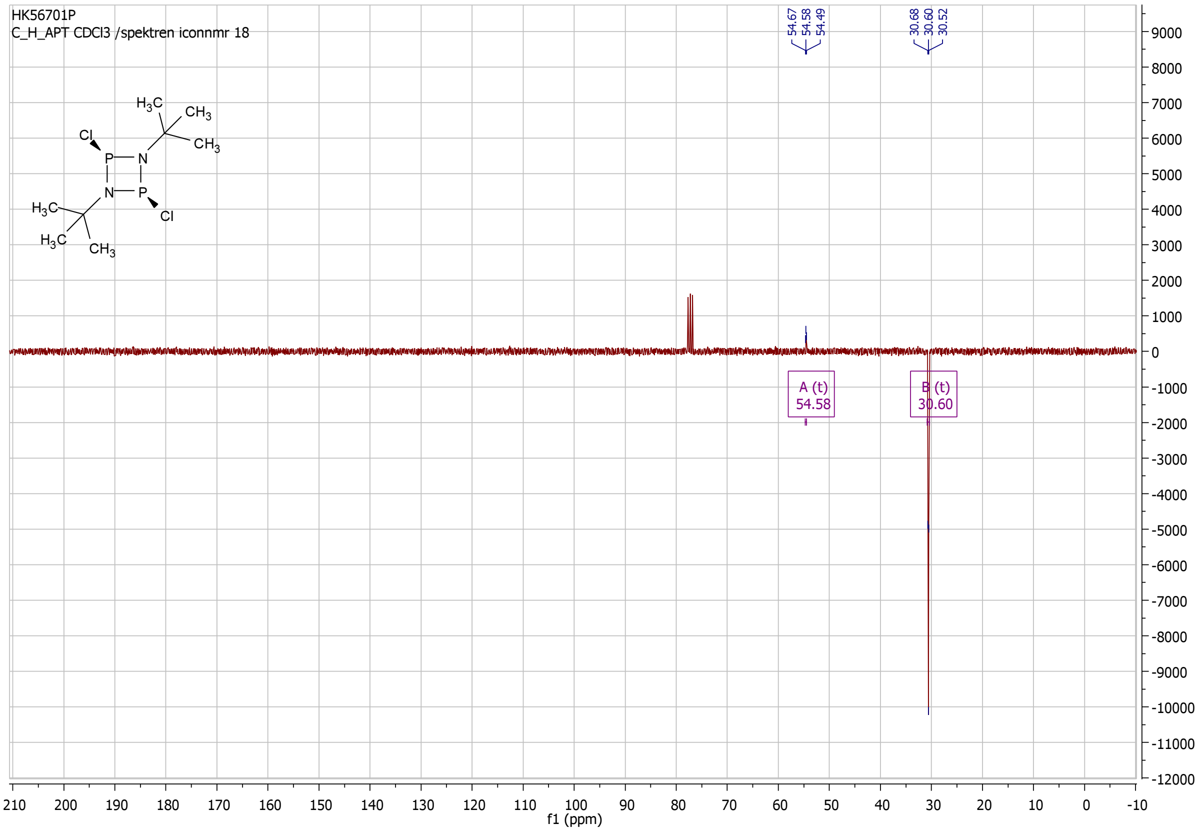
HK56701P
C_H_APT CDCl3 /spektren iconnmr 18
54.67
54.58
54.49
30.68
30.60
30.52
A (t)
54.58
B (t)
30.60
f1 (ppm)

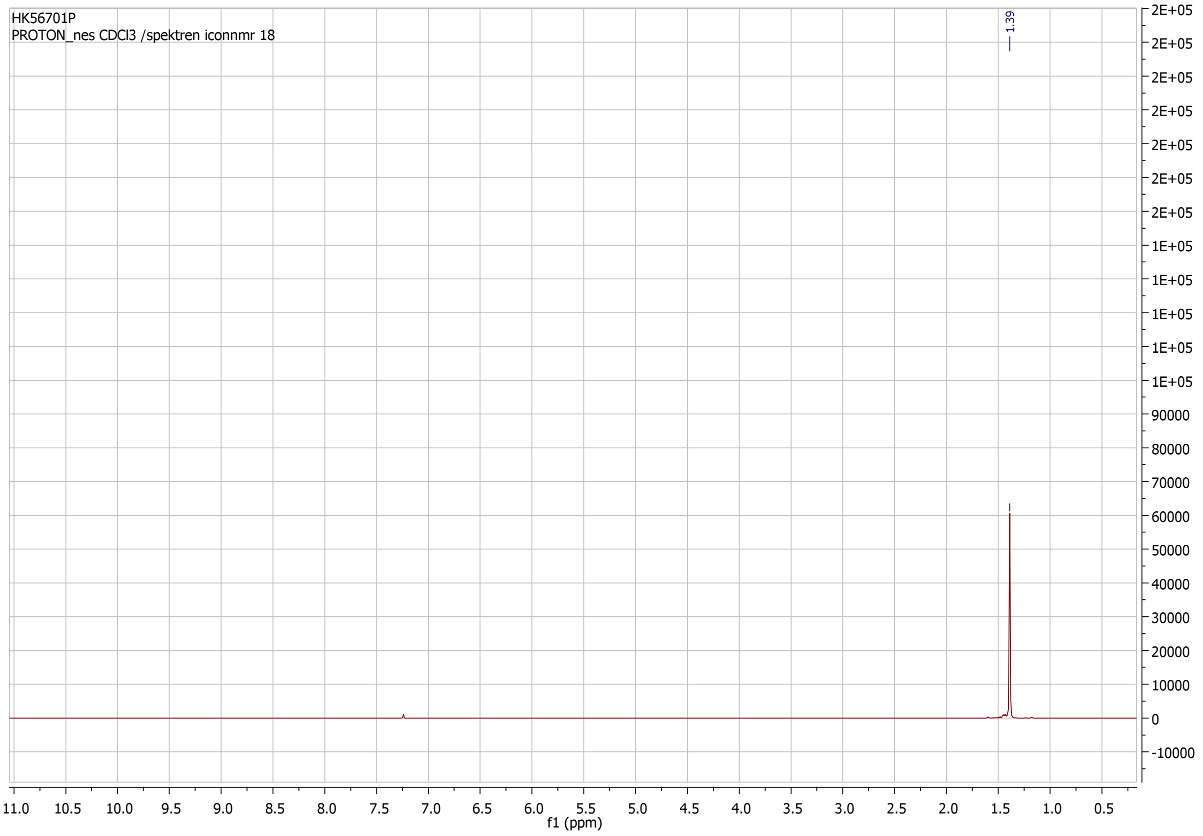
HK56701P
PROTON_nes CDCl3 /spektren iconnmr 18
1.39
f1 (ppm)

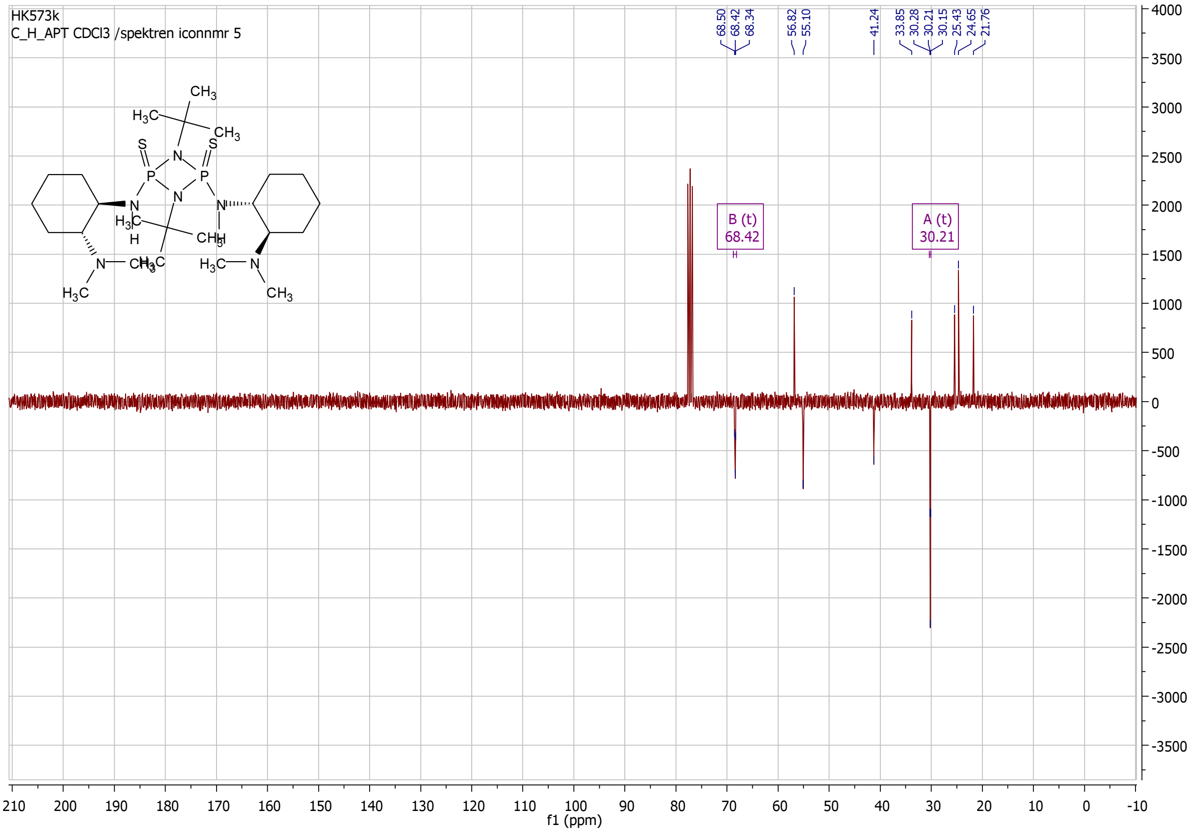
HK573k
C_H_APT CDCl3 /spektren iconnmr 5
68.50
68.42
68.34
56.82
55.10
41.24
33.85
30.28
30.21
30.15
25.43
24.65
21.76
B (t)
68.42
A (t)
30.21
f1 (ppm)

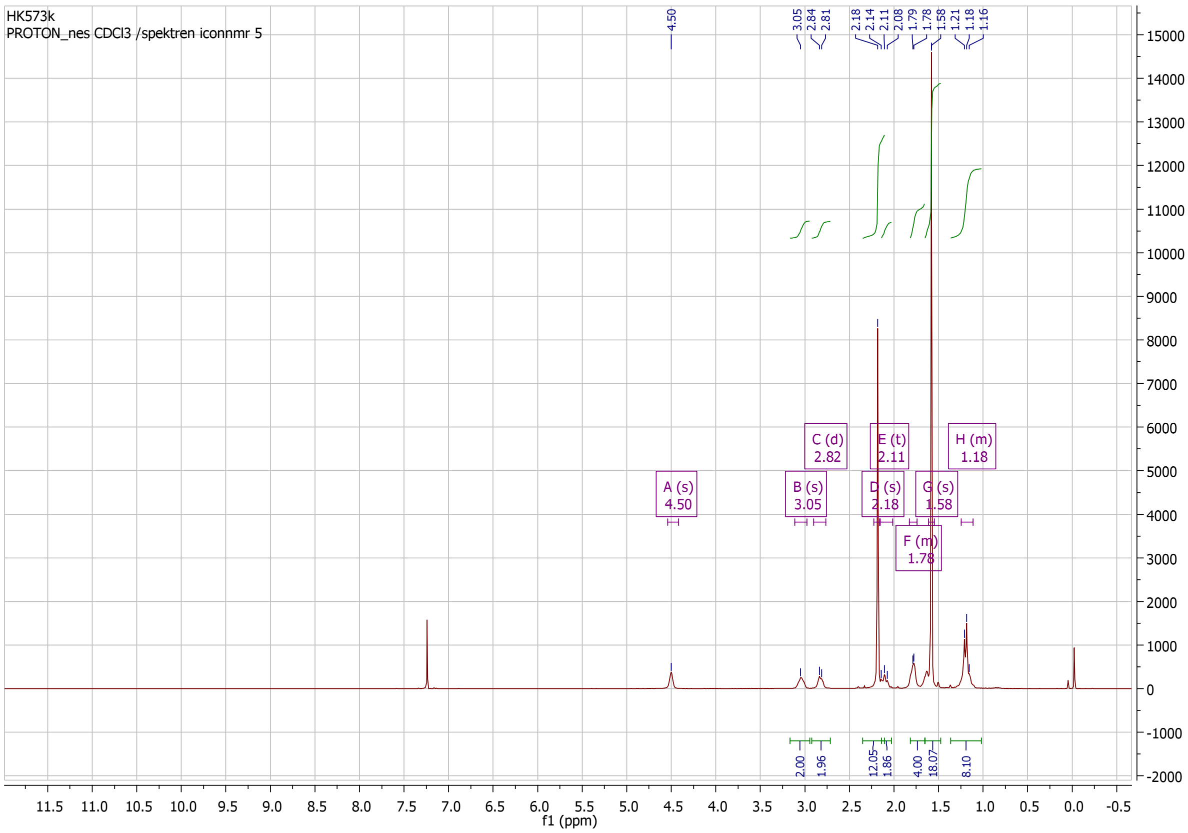
HK573k
PROTON_nes CDCl3 /spektren iconnmr 5
4.50
3.05
2.84
2.81
2.18
2.14
2.11
2.08
1.79
1.78
1.58
1.21
1.18
1.16
A (s)
4.50
B (s)
3.05
C (d)
2.82
D (s)
2.18
E (t)
2.11
F (m)
1.78
G (s)
1.58
H (m)
1.18
2.00
1.96
12.05
1.86
4.00
18.07
8.10
f1 (ppm)

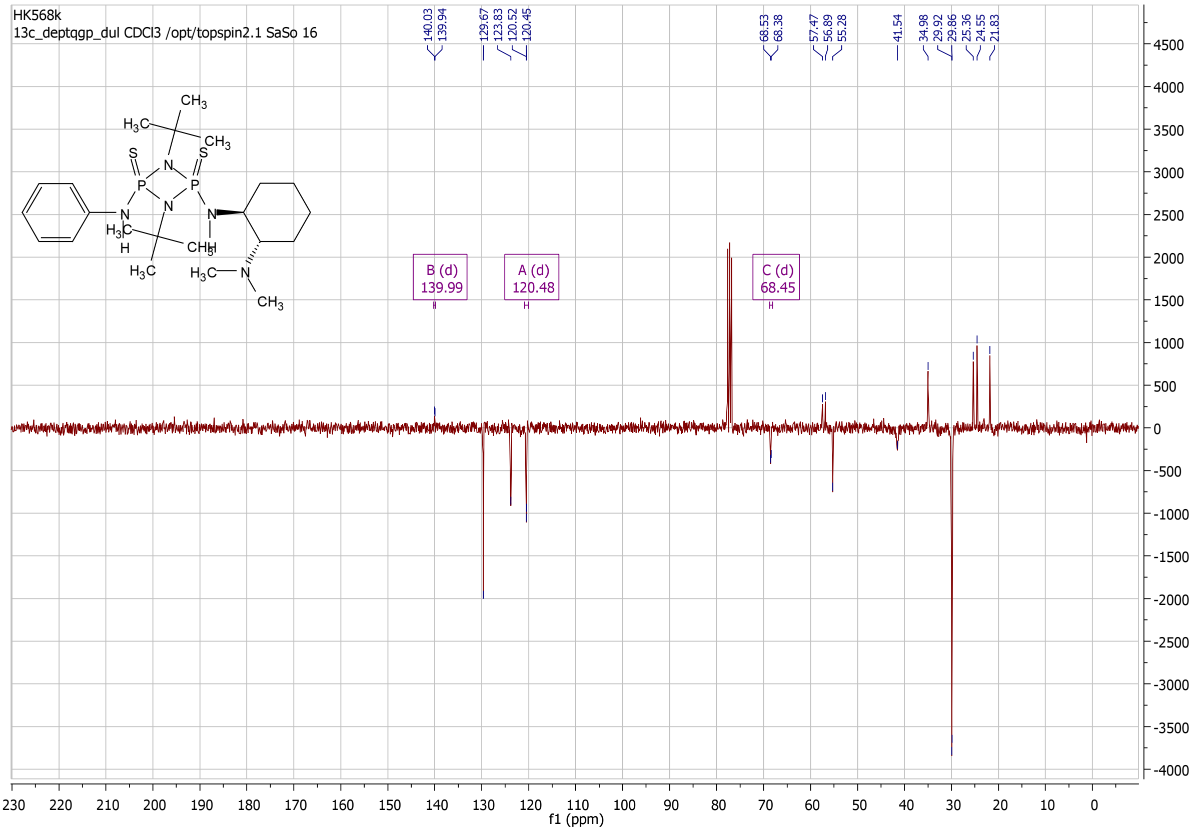
HK568k
13c_deptqgp_dul CDCl3 /opt/topspin2.1 SaSo 16
140.03
139.94
129.67
123.83
120.52
120.45
68.53
68.38
57.47
56.89
55.28
41.54
34.98
29.92
29.86
25.36
24.55
21.83
B (d)
139.99
A (d)
120.48
C (d)
68.45
f1 (ppm)

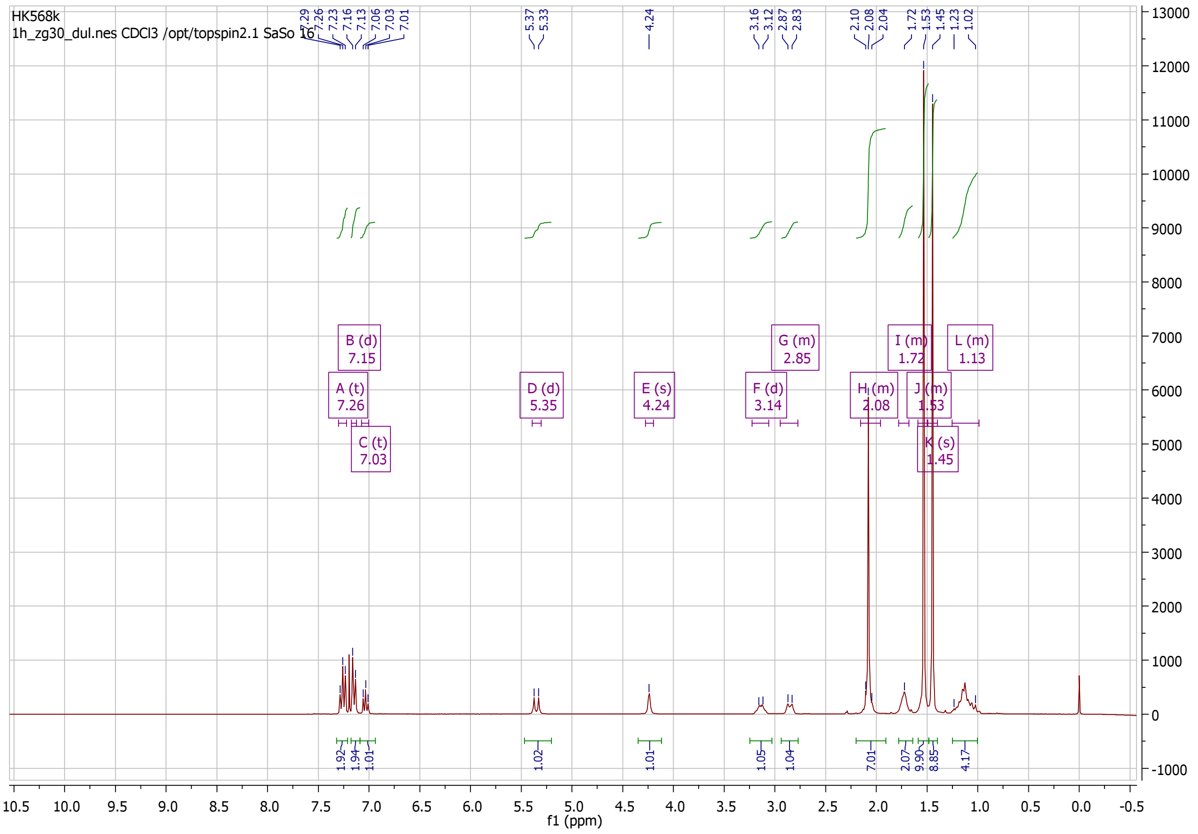
HK568k
1h_zg30_dul.nes CDCl3 /opt/topspin2.1 SaSo 16
7.29
7.26
7.23
7.16
7.13
7.06
7.03
7.01
5.37
5.33
4.24
3.16
3.12
2.87
2.83
2.10
2.08
2.04
1.72
1.53
1.45
1.23
1.02
B (d)
7.15
A (t)
7.26
C (t)
7.03
D (d)
5.35
E (s)
4.24
F (d)
3.14
G (m)
2.85
H (m)
2.08
I (m)
1.72
J (m)
1.53
K (s)
1.45
L (m)
1.13
1.92
1.94
1.01
1.02
1.01
1.05
1.04
7.01
2.07
9.90
8.85
4.17
f1 (ppm)

Computed structures

Stationary points of compounds shown in Scheme 2 (TPSS/def2-TZVP)

Nitrobenzene

Energy = -437.0025446832

N	2.3026023	-2.4446133	-0.2283546
O	1.7898057	-1.3366944	-0.4093276
O	1.6767375	-3.5042355	-0.1353929
C	3.7802223	-2.5071246	-0.1145949
C	4.3834401	-3.7435729	0.1001370
C	4.5112047	-1.3273604	-0.2268989
C	5.7713848	-3.7939711	0.2054012
H	3.7669578	-4.6311440	0.1805468
C	5.8983677	-1.3951174	-0.1202771
H	3.9911269	-0.3914396	-0.3937298
C	6.5267082	-2.6239960	0.0951749
H	6.2632017	-4.7472292	0.3738036
H	6.4887414	-0.4879298	-0.2058308
H	7.6088038	-2.6701426	0.1774149

Urea

Energy = -225.3938022577

C	-2.1903978	-2.4157126	-0.5272419
N	-1.4160692	-3.5315326	-0.8261857
N	-1.4492770	-1.3089568	-0.1269501
H	-0.5330126	-3.6394596	-0.3416993
H	-0.5087907	-1.2114408	-0.4907076
H	-1.9867210	-0.4512953	-0.1268111
H	-1.9583944	-4.3829854	-0.8989155
O	-3.4106971	-2.4081869	-0.6087887

Urea-Nitrobenze complex **Ia**

Energy = -662.4047657828

C	-2.2366690	-2.3818114	-0.5630741
O	-3.4580772	-2.3471535	-0.6799713
N	-1.4781938	-3.4875627	-0.9120450
N	-1.4969267	-1.3190818	-0.0711660
N	2.3282599	-2.4999201	-0.1700065
O	1.7879861	-1.4096639	-0.3920955
O	1.7142566	-3.5622373	-0.0142478
H	-0.5521164	-3.6003331	-0.5131954
H	-0.5064674	-1.2614525	-0.2833911
C	3.8013811	-2.5351930	-0.0858832
C	4.4284776	-3.7547185	0.1626998
C	4.5101458	-1.3478907	-0.2594624
C	5.8183256	-3.7787108	0.2388657
H	3.8307325	-4.6493257	0.2908872
C	5.8993465	-1.3909756	-0.1798321
H	3.9741556	-0.4260655	-0.4512083
C	6.5513166	-2.6015426	0.0682000

H	6.3293881	-4.7168270	0.4318571
H	6.4732619	-0.4791024	-0.3121431
H	7.6353966	-2.6277578	0.1286385
H	-1.9997409	-0.4425390	-0.0558311
H	-2.0181326	-4.3344728	-1.0248157

Thiourea

Energy = -548.3416535588

C	-2.1716569	-2.4157276	-0.5263981
N	-1.4172408	-3.5280963	-0.7922161
N	-1.4462018	-1.3123887	-0.1611096
H	-0.4990319	-3.6208510	-0.3726056
H	-0.4788916	-1.2308155	-0.4534907
H	-1.9717247	-0.4498693	-0.1223501
H	-1.9432161	-4.3842714	-0.9013962
S	-3.8327091	-2.4055405	-0.6381929

Thiourea-Nitrobenzene complexe **Ib**

Energy = -985.3546826253

C	-2.1707044	-2.3887276	-0.5441921
N	-1.4577405	-3.5367169	-0.6959629
N	-1.4356429	-1.2806886	-0.2577446
N	2.3075829	-2.4942557	-0.1943027
O	1.7623791	-1.3946282	-0.3585673
O	1.6915354	-3.5662431	-0.1037993
H	-0.4762422	-3.5976394	-0.4423571
H	-0.4236517	-1.2725923	-0.3421409
C	3.7756364	-2.5317713	-0.1004198
C	4.4040576	-3.7628429	0.0832434
C	4.4832430	-1.3344351	-0.1984802
C	5.7927665	-3.7877732	0.1721662
H	3.8080396	-4.6650649	0.1536404
C	5.8713563	-1.3793145	-0.1076234
H	3.9470216	-0.4040703	-0.3416074
C	6.5241005	-2.6008278	0.0775850
H	6.3044391	-4.7342731	0.3164729
H	6.4440896	-0.4601302	-0.1807952
H	7.6074705	-2.6280068	0.1486909
H	-1.9299522	-0.4016039	-0.2664302
H	-1.9906247	-4.3877917	-0.7895954
S	-3.8405278	-2.3427330	-0.6974674

Squaramide

Energy = -414.9861841254

C	-0.4139029	-0.1685634	-0.6179295
C	0.9677091	0.0050922	-0.6451320
C	0.8784311	1.4148553	-0.1786493
C	-0.6569153	1.2156860	-0.1300577
O	-1.6578684	1.8387226	0.1597800
O	1.7030901	2.2791220	0.0380440
N	-1.2821601	-1.1768376	-0.8578895
N	2.0441620	-0.7305702	-1.0005343
H	-1.0203276	-1.9663713	-1.4319424
H	1.9980977	-1.7388206	-1.0495795
H	2.9542544	-0.3395635	-0.7897898
H	-2.2667608	-0.9391221	-0.8599095

Squaramide-Nitrobenzene complex **II**

Energy = -852.0020366711

C	-0.4030616	-0.0779147	-0.7857935
C	0.9535991	-0.0285539	-0.4515634
C	0.8673969	1.3856862	-0.0052481
C	-0.6327388	1.3287906	-0.3688491
O	-1.6130719	2.0506074	-0.3535243
O	1.6782171	2.1766307	0.4407185
N	-1.2436938	-1.0042428	-1.2755050
N	1.9920044	-0.8788520	-0.5026753
O	-0.3036998	-3.8136671	-2.2546414
O	1.7127932	-3.8610923	-1.4045546
H	-0.9290075	-1.9106497	-1.6032566
H	1.8921903	-1.8520275	-0.7681403
N	0.7713026	-4.3845336	-2.0181370
H	2.8824929	-0.5677235	-0.1396953
H	-2.1970458	-0.7283891	-1.4676092
C	0.9387223	-5.7650572	-2.4942160
C	2.1167451	-6.4435151	-2.1828841
C	-0.0872365	-6.3454499	-3.2396676
C	2.2643559	-7.7507931	-2.6362519
H	2.8844160	-5.9491167	-1.5998418
C	0.0808057	-7.6518722	-3.6882657
H	-0.9834826	-5.7762131	-3.4550781
C	1.2512179	-8.3529901	-3.3868763
H	3.1711806	-8.3003583	-2.4038362
H	-0.7027032	-8.1233835	-4.2731160
H	1.3744886	-9.3731065	-3.7386505

Phosphorustriamide (open chain)

Energy = -584.7128977733

N	-1.9276303	-3.5729937	0.4978181
N	-2.5822086	-1.4252961	-1.2558295
H	-0.9256664	-3.4266128	0.4409421
H	-1.6222686	-1.1365345	-1.4096062
P	-2.9637664	-2.3077662	0.1197841
H	-2.8464473	-0.2860419	1.1899825
O	-4.3573119	-2.8017827	0.0358479
N	-2.4247280	-1.2070207	1.2649614
H	-2.2160775	-4.4765377	0.1403108
H	-2.4527402	-1.5515120	2.2200718
H	-3.0422318	-1.7393379	-2.1025987

Phosphorustriamide-Nitrobenzene complex **III**

Energy = -1021.722293751

N	-2.1936822	-3.6765784	1.3649879
N	-3.0035359	-2.1060319	-0.8569782
H	-1.2136434	-3.5464200	1.1237095
H	-2.0344854	-1.9600723	-1.1306645
P	-3.2962724	-2.5825600	0.7269810
H	-3.2052836	-0.3450148	1.2034140
O	-4.6815234	-3.0917837	0.8713656
N	-2.7454950	-1.1991780	1.5043338
O	0.9369455	-3.1508332	0.2126640
N	1.2043544	-2.4688129	-0.7829302
O	0.3614608	-1.9602802	-1.5303667
H	-2.4875797	-4.6432413	1.2750340
H	-2.7250032	-1.2744181	2.5169714
H	-3.5190966	-2.6484740	-1.5418307
C	2.6306266	-2.2486549	-1.1023749
C	2.9562462	-1.4849210	-2.2213715
C	3.5969875	-2.8144035	-0.2731222
C	4.3023622	-1.2855672	-2.5160254
H	2.1661591	-1.0660217	-2.8330967
C	4.9380647	-2.6045490	-0.5827679
H	3.2906056	-3.3993455	0.5858838
C	5.2899630	-1.8436003	-1.7003925
H	4.5803616	-0.6942291	-3.3829605
H	5.7087210	-3.0354209	0.0488024
H	6.3381753	-1.6847709	-1.9366382

Cyclophosphazane

Energy = -2164.464585537

S	-1.4572065	2.0187056	1.6885690
S	2.1120576	-1.5498423	1.6906168
N	-0.4689588	-0.5630343	0.1196281
N	1.1247690	1.0309460	0.1196294
C	-1.4680391	-1.5622594	0.1707745
C	-1.1098741	-2.9180593	0.1849540
C	-2.8239165	-1.2043413	0.1837542
C	-2.1007861	-3.8968582	0.2031078
H	-0.0623214	-3.2015559	0.1994478
C	-3.8025351	-2.1954284	0.2020100
H	-3.1076144	-0.1568286	0.1971177
C	-3.4508578	-3.5454314	0.2086598
H	-1.8092739	-4.9433925	0.2182297
H	-4.8491321	-1.9041069	0.2164707
H	-4.2185603	-4.3132672	0.2247137
C	2.1238393	2.0301812	0.1707761
C	1.7657088	3.3859990	0.1841025
C	3.4796989	1.6722283	0.1846083
C	2.7566456	4.3647724	0.2022711
H	0.7181553	3.6695270	0.1979155
C	4.4583429	2.6632907	0.2028526
H	3.7633658	0.6247160	0.1986420
C	4.1067047	4.0133065	0.2086568
H	2.4651613	5.4113236	0.2167700
H	5.5049227	2.3719412	0.2179582
H	4.8744237	4.7811261	0.2246958
P	1.2402904	-0.6782931	0.2189603
P	-0.5844800	1.1462044	0.2178804
H	2.5219979	-1.9597977	-1.2245577
H	1.4833844	-0.9215979	-2.1281262
H	-1.8657699	2.4272923	-1.2253667
N	1.8078911	-1.2458810	-1.2263042
H	-0.8275030	1.3894516	-2.1298039
N	-1.1516389	1.7133578	-1.2277363

Cyclophosphazane-Nitrobenzene complex **IV**

Energy = -2601.478648201

S	-1.1490962	1.5451806	2.1521885
S	2.4457587	-2.0586348	2.0852307
N	-0.1305581	-1.0114653	0.5615417
N	1.4580226	0.5860281	0.6204687
C	-1.1248172	-2.0130664	0.5707111
C	-0.7628061	-3.3684538	0.5741416
C	-2.4830073	-1.6614762	0.5587957
C	-1.7496410	-4.3514054	0.5577019
H	0.2854514	-3.6479163	0.6109107
C	-3.4572419	-2.6565672	0.5439506
H	-2.7706034	-0.6152650	0.5734583
C	-3.1011072	-4.0057217	0.5402355
H	-1.4538825	-5.3969824	0.5659135
H	-4.5051740	-2.3691552	0.5396956
H	-3.8660506	-4.7766166	0.5311214
C	2.4504981	1.5859668	0.6980962
C	2.0865479	2.9392942	0.7640904
C	3.8092794	1.2363603	0.6920206
C	3.0719621	3.9225181	0.8137128
H	1.0373952	3.2159122	0.7978603
C	4.7820672	2.2316532	0.7433821
H	4.0980820	0.1908857	0.6598374
C	4.4240610	3.5790924	0.8010762
H	2.7744345	4.9661288	0.8697734
H	5.8303537	1.9455097	0.7424849
H	5.1878716	4.3500113	0.8431110
P	1.5840937	-1.1220385	0.6444089
P	-0.2577603	0.6925493	0.6776638
H	2.7582067	-2.4185479	-0.8464882
H	1.7907726	-1.2833152	-1.7076513
H	-1.3954953	2.0780432	-0.7563038
N	2.1901001	-1.5829346	-0.8213812
O	0.1982564	0.9207475	-3.7692540
N	0.8273724	0.0402834	-4.3688447
O	1.3845438	-0.9153594	-3.8145956
H	-0.3909803	1.0150184	-1.6608684
C	0.9191328	0.1352364	-5.8365742
C	1.6200193	-0.8513889	-6.5283154
C	0.3015472	1.2079354	-6.4778522
C	1.7015283	-0.7534335	-7.9142604
H	2.0818271	-1.6655285	-5.9828600
C	0.3940784	1.2893314	-7.8642458
H	-0.2309846	1.9491385	-5.8941534
C	1.0911272	0.3126023	-8.5800595
H	2.2415713	-1.5099105	-8.4749073
H	-0.0780888	2.1158088	-8.3860428
H	1.1590972	0.3825662	-9.6617371
N	-0.8346121	1.2369483	-0.7725089

Transition states (TPSS/def-SVP)

ProR

RRO1O2 = TS14b

Energy = -4023.993093528 vi -306.640015

C	2.2277137	0.8394790	-3.1136250
C	2.6053345	0.7820642	-4.4695255
C	3.1857974	1.1635279	-2.1328556
C	3.9376067	1.0234193	-4.8311059
H	1.8492443	0.5418646	-5.2217410
C	4.5117741	1.4236332	-2.5088085
H	2.8845754	1.2062926	-1.0814378
C	4.8936653	1.3472073	-3.8568831
H	4.2256778	0.9707979	-5.8865704
H	5.2525990	1.6737782	-1.7405780
H	5.9318595	1.5411184	-4.1473135
C	-2.8212137	0.3987590	-2.6202854
C	-3.4704604	-0.7819009	-2.2042033
C	-3.5826877	1.5174340	-3.0183372
C	-4.8708259	-0.8268302	-2.1892181
H	-2.8804059	-1.6571384	-1.9035290
C	-4.9823498	1.4608675	-2.9723852
H	-3.0707512	2.4160051	-3.3767815
C	-5.6348262	0.2899007	-2.5611749
H	-5.3702547	-1.7478314	-1.8684883
H	-5.5643158	2.3365038	-3.2807937
H	-6.7287621	0.2455468	-2.5366752
N	-1.4025872	0.4590567	-2.6335216
N	0.8780187	0.5982946	-2.7136107
S	-0.3838253	3.3805992	-3.5356195
S	-0.1933595	-1.3589557	-4.9982625
C	-0.8388756	3.4457863	-0.2511167
C	-0.0155894	4.0729471	0.8986523
C	-2.3002712	3.2898738	0.2261371
H	-0.8365209	4.1427363	-1.1109142
C	-0.5659475	5.4649697	1.2426082
H	-0.0694098	3.4290148	1.7924356
C	-2.9002743	4.6433087	0.6385266
H	-2.3277822	2.5771612	1.0721994
H	-2.8873307	2.8410841	-0.5933628
C	-2.0344633	5.3424310	1.6969315
H	0.0237669	5.9283876	2.0516516
H	-0.5052048	6.1311256	0.3602316
H	-3.9276879	4.5022291	1.0188510
H	-2.9801534	5.2906693	-0.2562332
H	-2.4314021	6.3475491	1.9252432
H	-2.0645686	4.7647159	2.6401113
C	0.1694769	-3.3159245	-2.2674536
C	-0.7944681	-4.2712782	-1.5145096
C	1.6356614	-3.5831590	-1.8566924

H	0.0763557	-3.4808088	-3.3566582
C	-0.3616722	-5.7414854	-1.7513100
H	-0.6755249	-4.0522158	-0.4363384
C	2.0479858	-5.0488598	-2.0602409
H	1.7553791	-3.3058660	-0.7917260
C	1.0916855	-5.9956243	-1.3203992
H	-1.0458605	-6.4156901	-1.2043474
H	-0.4666749	-5.9834206	-2.8263465
H	3.0892271	-5.2003802	-1.7193573
H	2.0334589	-5.2870440	-3.1417118
H	1.3662075	-7.0502329	-1.5075291
H	1.1855901	-5.8315405	-0.2282310
C	1.8774143	4.6280051	-0.7408842
H	1.3173120	4.1312457	-1.5470100
H	2.9554575	4.4593702	-0.8807629
H	1.6669905	5.7082242	-0.7441081
C	2.3108383	4.6665491	1.6925162
H	1.9844387	4.2111250	2.6378037
H	2.1851422	5.7584634	1.6909224
H	3.3627769	4.4115570	1.4967817
N	1.4887589	4.0620365	0.5902641
N	-0.2488076	2.1973949	-0.7549691
N	-0.1949828	-1.9139143	-2.0264963
P	-0.2238012	-0.7131916	-3.1615034
P	-0.3180106	1.7886482	-2.3965612
O	-0.2770041	0.2971182	1.4718205
O	-0.8266374	-1.7424219	0.7724976
N	-0.6932456	-0.8980909	1.7013792
H	-1.1971678	-2.2976759	3.1481122
H	-1.1120068	0.7648443	3.6795764
C	-1.4483321	-0.5820981	5.3672401
C	-2.0654847	0.4426940	6.1194102
C	-1.3661730	-1.8747644	5.9292718
C	-2.6034589	0.1806833	7.3837798
H	-2.1248759	1.4525697	5.6974576
C	-1.9009431	-2.1344177	7.1963473
H	-0.8659355	-2.6784485	5.3818163
C	-2.5230924	-1.1110692	7.9264428
H	-3.0866303	0.9853368	7.9481706
H	-1.8248287	-3.1415367	7.6195596
H	-2.9399493	-1.3183951	8.9178290
C	-0.9672704	-1.2431803	2.9988805
C	-0.9114690	-0.2587963	4.0211453
H	-0.2851851	1.3906539	-0.0985338
H	-0.3589765	-1.6521271	-1.0350370
O	0.9773456	2.4532791	3.3903040
C	1.3477654	1.2632140	3.3573298
C	2.1637023	0.7609418	2.1618755
C	1.0279748	0.3218031	4.4183938
C	2.6819292	-0.6329714	2.1995145
C	1.7444370	-0.9561203	4.5482952
H	0.7646050	0.7960964	5.3694845
C	3.4299811	-1.1299911	1.1124854
C	2.4653566	-1.4627404	3.3308000
C	3.9426107	-2.4300575	1.1414173
H	3.5961116	-0.4834258	0.2472675

C	2.9926842	-2.7654859	3.3523739
C	3.7227798	-3.2512053	2.2626955
H	4.5178885	-2.8071642	0.2895173
H	2.8236206	-3.3679347	4.2504176
H	4.1305920	-4.2675311	2.2847399
O	2.4616417	1.5315943	1.2327989
O	1.7266158	-1.6208563	5.5895019
H	1.7434090	3.0358291	0.6349705
H	2.2889893	-2.9104719	-2.4410525
N	-2.2088160	-3.9879544	-1.8140430
C	-2.6524149	-4.2969474	-3.1700661
H	-3.6627985	-3.8769732	-3.3198252
H	-2.7072002	-5.3881176	-3.3931044
H	-1.9863735	-3.8180733	-3.9066339
C	-3.1229154	-4.4804688	-0.7917627
H	-3.1937488	-5.5938789	-0.7358701
H	-4.1400730	-4.1000248	-0.9962538
H	-2.8059516	-4.1020706	0.1951780

RRO2

Energy = -4023.985566964 vi -251.779999

C	3.0592520	0.3252216	-1.2971107
C	4.2641723	0.2368891	-2.0215363
C	3.0866228	0.4867389	0.1002813
C	5.4877281	0.2936832	-1.3415989
H	4.2257977	0.1158030	-3.1073119
C	4.3166392	0.5574736	0.7704228
H	2.1538775	0.5696285	0.6643619
C	5.5175037	0.4553224	0.0523253
H	6.4230553	0.2186662	-1.9069611
H	4.3168443	0.6856546	1.8573731
H	6.4778079	0.5029171	0.5779665
C	-0.7691246	0.3392459	-4.6521696
C	-1.7527590	-0.6671614	-4.7355089
C	-0.7729078	1.4055950	-5.5755804
C	-2.7260095	-0.5944742	-5.7412377
H	-1.7497245	-1.4983940	-4.0187702
C	-1.7695391	1.4746933	-6.5577778
H	0.0114213	2.1664982	-5.5163306
C	-2.7485231	0.4746510	-6.6495192
H	-3.4877688	-1.3800106	-5.8048620
H	-1.7659994	2.3092051	-7.2679373
H	-3.5197634	0.5255447	-7.4255690
N	0.2251103	0.2922396	-3.6408020
N	1.8019767	0.2611477	-1.9811172
S	1.8241154	3.0920231	-3.4507611
S	2.3813741	-2.0462800	-4.1119416
C	-1.0123526	3.2610407	-1.6194805
C	-1.3397994	3.8055996	-0.2122648
C	-2.3362481	3.0909581	-2.3978331
H	-0.3779459	3.9901865	-2.1598289
C	-2.0485496	5.1644629	-0.2981282
H	-1.9945500	3.0711609	0.2952897
C	-3.1069119	4.4162680	-2.4916807

H	-2.9423353	2.3180711	-1.8894641
H	-2.0999632	2.7083956	-3.4056761
C	-3.3576405	5.0159487	-1.1004276
H	-2.2851830	5.5508365	0.7081169
H	-1.3919091	5.9051749	-0.7945516
H	-4.0667355	4.2594579	-3.0149111
H	-2.5269125	5.1349248	-3.1029989
H	-3.8466177	6.0029976	-1.1798084
H	-4.0471223	4.3610080	-0.5352985
C	0.2493240	-3.4129892	-1.8731872
C	-1.1298256	-4.1197480	-1.9536450
C	0.9442244	-3.7057576	-0.5238351
H	0.9010693	-3.7953168	-2.6798604
C	-0.9585406	-5.6357750	-1.6698673
H	-1.7540743	-3.6961031	-1.1404323
C	1.0853339	-5.2099652	-0.2466840
H	0.3539669	-3.2398948	0.2896985
C	-0.2808443	-5.9070347	-0.3179588
H	-1.9473800	-6.1278253	-1.7063531
H	-0.3485500	-6.0872121	-2.4755876
H	1.5549925	-5.3691616	0.7414815
H	1.7660673	-5.6581475	-0.9965727
H	-0.1708416	-6.9956281	-0.1592095
H	-0.9270459	-5.5326229	0.5008884
C	1.1341501	4.4564047	0.0644703
H	1.3778321	3.9618755	-0.8880916
H	1.9671645	4.3274154	0.7705770
H	0.9353054	5.5264453	-0.0962126
C	-0.3458089	4.3801479	2.0387752
H	-1.2542378	3.9145153	2.4446341
H	-0.4692974	5.4696138	1.9707017
H	0.5150934	4.1363178	2.6758092
N	-0.0809425	3.8189193	0.6745076
N	-0.2345145	2.0241682	-1.5078042
N	0.1137778	-1.9712778	-2.1082553
P	1.1580331	-1.0277155	-2.9957047
P	0.9064920	1.5545667	-2.6647402
O	-2.7629389	0.9988019	1.0600505
O	-1.7511890	-0.1131657	-0.5675397
N	-2.0010242	0.0593457	0.6847028
H	-0.6757855	-1.4540118	1.1778392
H	-2.7623897	-0.4143053	3.2044818
C	-1.1926865	-1.7782570	3.8624442
C	-2.0115755	-2.3089460	4.8803581
C	0.1056322	-2.3072206	3.6965088
C	-1.5534050	-3.3513278	5.6975955
H	-3.0161265	-1.8983031	5.0282528
C	0.5633629	-3.3472143	4.5151424
H	0.7692804	-1.9004547	2.9247048
C	-0.2659339	-3.8752916	5.5165618
H	-2.2063786	-3.7552994	6.4787321
H	1.5737876	-3.7444259	4.3714147
H	0.0928164	-4.6894254	6.1553818
C	-1.4155035	-0.7732292	1.5954497
C	-1.7148321	-0.6735436	2.9944788
H	-0.8050327	1.2138759	-1.1605638

H	-0.5726933	-1.4627292	-1.5242845
O	0.5592206	1.4771192	2.0679756
C	0.2575763	1.1707867	3.2435363
C	1.3841227	0.8777659	4.2398005
C	-1.1215995	0.9940292	3.6601747
C	1.0051832	0.7325337	5.6760656
C	-1.4632493	0.9919837	5.1074489
H	-1.8432844	1.5466984	3.0390694
C	2.0216636	0.5483580	6.6330577
C	-0.3488734	0.7990351	6.0927244
C	1.6993918	0.4327832	7.9889332
H	3.0573339	0.5004631	6.2822629
C	-0.6613108	0.6851537	7.4584051
C	0.3560665	0.5041139	8.4020839
H	2.4929070	0.2887814	8.7301413
H	-1.7152666	0.7430115	7.7475480
H	0.1067876	0.4173618	9.4651496
O	2.5500351	0.8205093	3.8579576
O	-2.6337792	1.1011889	5.4830121
H	0.1449767	2.8046908	0.8451390
H	1.9323516	-3.2129515	-0.5276197
N	-1.8472078	-3.8021898	-3.2004386
C	-1.2411451	-4.3034962	-4.4317157
H	-1.7765626	-3.8689620	-5.2939673
H	-1.2798777	-5.4128469	-4.5378349
H	-0.1897928	-3.9790428	-4.5004942
C	-3.2780747	-4.0684502	-3.1310991
H	-3.7747738	-3.6322838	-4.0162231
H	-3.7012079	-3.5899174	-2.2307888
H	-3.5428599	-5.1527735	-3.1033821

RSO1

Energy = -4023.978562771 v_i -111.0000

C	3.0318650	0.7790882	-1.0583916
C	3.8065313	1.9576445	-0.9954365
C	3.4338433	-0.3534805	-0.3190603
C	4.9460128	2.0047654	-0.1800547
H	3.5214928	2.8140790	-1.6150211
C	4.5886170	-0.3002217	0.4705421
H	2.8294049	-1.2632575	-0.3693365
C	5.3453969	0.8787821	0.5553665
H	5.5437511	2.9229615	-0.1451152
H	4.8927606	-1.1885724	1.0348623
H	6.2439862	0.9157432	1.1799509
C	-1.0151286	0.8065186	-4.1625134
C	-2.3392022	0.5112335	-3.7895204
C	-0.7213586	1.2391779	-5.4687223
C	-3.3713916	0.6678697	-4.7245290
H	-2.5304503	0.1412880	-2.7772765
C	-1.7598638	1.3831141	-6.3984813
H	0.3160707	1.4576956	-5.7359362
C	-3.0843428	1.1049905	-6.0272031
H	-4.4027208	0.4355990	-4.4375373
H	-1.5339397	1.7186475	-7.4164996

H	-3.8934678	1.2234788	-6.7563176
N	0.0237152	0.6926136	-3.1905315
N	1.8647687	0.7262727	-1.8508429
S	1.1461329	3.6288522	-3.1425800
S	2.4515384	-1.0538912	-4.3913785
C	-0.8994125	3.4025216	-0.5074460
C	-0.6415668	3.9483349	0.9208777
C	-2.4151481	3.1756306	-0.6895212
H	-0.5769276	4.1633273	-1.2437688
C	-1.3735813	5.2767702	1.1433773
H	-0.9907123	3.2015132	1.6534765
C	-3.2093562	4.4655009	-0.4256913
H	-2.7463918	2.3693406	-0.0077259
H	-2.5899094	2.8150335	-1.7173036
C	-2.8893914	5.0588169	0.9553210
H	-1.1786011	5.6717991	2.1564956
H	-1.0208520	6.0327482	0.4165968
H	-4.2921453	4.2655531	-0.5099999
H	-2.9636503	5.2087366	-1.2080929
H	-3.4158235	6.0187714	1.0982761
H	-3.2539270	4.3756722	1.7464523
C	0.7143915	-3.1891831	-2.3198034
C	-0.6319582	-3.7369362	-2.8711463
C	1.1449162	-3.9477864	-1.0438318
H	1.5000834	-3.3049774	-3.0894022
C	-0.5513932	-5.2734825	-3.0572867
H	-1.3902168	-3.5275911	-2.0937465
C	1.1873811	-5.4707919	-1.2421468
H	0.4393628	-3.7065743	-0.2257219
C	-0.1598966	-5.9955539	-1.7592463
H	-1.5252440	-5.6441546	-3.4262428
H	0.1965194	-5.5114181	-3.8384647
H	1.4599936	-5.9614189	-0.2896369
H	1.9818271	-5.7268851	-1.9709842
H	-0.1109742	-7.0876984	-1.9274055
H	-0.9390987	-5.8242737	-0.9915327
C	1.6401773	4.9043619	0.2818501
H	1.4546036	4.6214891	-0.7679622
H	2.7080129	4.7774928	0.5112441
H	1.3389703	5.9451153	0.4703058
C	1.2041122	4.2637381	2.6387360
H	0.7844666	3.4560741	3.2545105
H	0.8268856	5.2569152	2.9200307
H	2.2997671	4.2427455	2.7307814
N	0.8667135	4.0012892	1.1965307
N	-0.0817802	2.2161788	-0.7695325
N	0.6364753	-1.7484155	-2.0223718
P	1.2597381	-0.5293949	-2.9532660
P	0.7237267	1.9367255	-2.2428183
O	-1.3794342	-0.5521277	-0.2664444
O	-2.8164146	-2.2054051	-0.6397759
N	-2.2401830	-1.4293120	0.1619278
H	-3.1307246	-2.3246340	1.8031908
C	-2.4890235	-1.4978227	1.5027268
H	-0.4174959	1.3475189	-0.3184747
H	-0.1509278	-1.4402241	-1.4132995

O	0.5294507	1.4300080	2.2061520
C	0.4964157	0.4129824	2.9247638
C	0.9406855	0.5322522	4.3830160
C	-0.0865913	-0.8423824	2.4422013
C	1.0870991	-0.7279939	5.1602630
C	0.2132823	-2.0970896	3.1910277
H	0.0467920	-0.9472503	1.3531789
C	1.5836351	-0.6682374	6.4768752
C	0.7565211	-1.9839010	4.5867348
C	1.7595501	-1.8416546	7.2169815
H	1.8256375	0.3150036	6.8924345
C	0.9433702	-3.1566940	5.3386310
C	1.4429583	-3.0873643	6.6445101
H	2.1471756	-1.7918656	8.2399959
H	0.6870806	-4.1111928	4.8683528
H	1.5885031	-4.0062424	7.2225935
O	1.1905622	1.6417105	4.8562626
O	-0.0202362	-3.2007260	2.6922501
H	1.1434490	3.0139721	0.9991814
C	-1.8485176	-0.5656710	2.4038504
H	-1.7823348	0.4391490	1.9538227
C	-2.4115720	-0.5211704	3.8015157
C	-2.5889425	0.7198685	4.4468032
C	-2.7563865	-1.6954671	4.5035999
C	-3.1008304	0.7903338	5.7501243
H	-2.3267051	1.6424261	3.9157874
C	-3.2684260	-1.6261106	5.8061883
H	-2.6169325	-2.6720572	4.0286353
C	-3.4434013	-0.3841601	6.4345259
H	-3.2348555	1.7657886	6.2299648
H	-3.5315539	-2.5493605	6.3334731
H	-3.8450347	-0.3326589	7.4520473
H	2.1362129	-3.5739312	-0.7296275
N	-1.0784573	-2.9943424	-4.0632784
C	-0.3986127	-3.3147479	-5.3140348
H	-0.6624226	-2.5526456	-6.0684823
H	-0.6722671	-4.3126485	-5.7334109
H	0.6944115	-3.2728668	-5.1793372
C	-2.5288861	-2.9917655	-4.2191604
H	-2.8075055	-2.2876279	-5.0223576
H	-2.9994926	-2.6567992	-3.2806160
H	-2.9515900	-3.9908212	-4.4903188

RSO1O2

Energy = -4023.981503765 v_i -247.279999

C	2.7616150	0.2730005	-0.3671535
C	3.4860321	-0.8931187	-0.0376902
C	2.9354858	1.4305531	0.4208902
C	4.3470762	-0.8957298	1.0662571
H	3.3932463	-1.7847120	-0.6629319
C	3.8070591	1.4155999	1.5158597
H	2.3859038	2.3403341	0.1708137
C	4.5150792	0.2528613	1.8526700
H	4.8985986	-1.8113304	1.3054581

H	3.9271180	2.3259643	2.1134809
H	5.1927567	0.2444957	2.7125661
C	0.1187164	0.4391105	-4.7248074
C	-0.7484852	-0.5968516	-5.1307597
C	0.3603278	1.5316300	-5.5839680
C	-1.3631005	-0.5261130	-6.3881333
H	-0.9302709	-1.4536392	-4.4685385
C	-0.2841352	1.5965757	-6.8268511
H	1.0596793	2.3138922	-5.2742944
C	-1.1458736	0.5693036	-7.2380081
H	-2.0313564	-1.3361532	-6.7006818
H	-0.0921807	2.4507119	-7.4855701
H	-1.6388107	0.6181364	-8.2147018
N	0.7418606	0.3753216	-3.4487255
N	1.8612574	0.2810341	-1.4577540
S	2.2719682	3.1410656	-2.8934916
S	2.9533220	-1.9353007	-3.4813081
C	-0.9509907	3.2684004	-1.7934800
C	-1.6531158	3.8706432	-0.5560855
C	-2.0293022	2.9258239	-2.8497380
H	-0.2633558	4.0195027	-2.2284576
C	-2.4383766	5.1373236	-0.9307317
H	-2.3444646	3.1078681	-0.1539689
C	-2.8654918	4.1542837	-3.2368108
H	-2.6742928	2.1241716	-2.4424874
H	-1.5244707	2.5107159	-3.7383174
C	-3.4988377	4.8075838	-2.0003503
H	-2.9370827	5.5650443	-0.0443722
H	-1.7457395	5.9060031	-1.3240140
H	-3.6484440	3.8657652	-3.9598514
H	-2.2176914	4.8909318	-3.7508862
H	-4.0373560	5.7318623	-2.2745586
H	-4.2477030	4.1206509	-1.5614260
C	0.3369259	-3.4011864	-1.8599740
C	-0.9453743	-4.1077414	-2.3747889
C	0.5598143	-3.7053353	-0.3624762
H	1.2121378	-3.7594200	-2.4320866
C	-0.8578490	-5.6281585	-2.0783249
H	-1.7844975	-3.6939220	-1.7830257
C	0.6130632	-5.2112706	-0.0664019
H	-0.2579960	-3.2419971	0.2179904
C	-0.6491065	-5.9167847	-0.5831179
H	-1.7780928	-6.1236257	-2.4391054
H	-0.0162057	-6.0644538	-2.6504189
H	0.7342547	-5.3690621	1.0206318
H	1.5036711	-5.6543568	-0.5548989
H	-0.5854421	-7.0073496	-0.4106962
H	-1.5290372	-5.5562916	-0.0145252
C	0.5739286	4.8513784	0.2482802
H	1.0911357	4.3443701	-0.5808804
H	1.2160380	4.8600671	1.1408130
H	0.3054618	5.8803142	-0.0340202
C	-1.3298276	4.6792197	1.8188635
H	-2.2861336	4.1606490	1.9812039
H	-1.5003286	5.7539400	1.6651362
H	-0.6653684	4.4915079	2.6732609

N	-0.6707361	4.0921973	0.6013234
N	-0.1330856	2.1147477	-1.4061907
N	0.2532767	-1.9498716	-2.0977430
P	1.4787427	-0.9820657	-2.6500655
P	1.1938023	1.6137102	-2.3195024
O	-1.8331021	0.6777311	0.1519516
O	-2.2791055	-1.3150053	-0.7132342
N	-2.1496302	-0.5647419	0.2984190
H	-2.5241475	-2.1122154	1.6276902
H	-2.1933709	0.8659492	2.4377192
C	-2.4449739	-0.5848603	4.0416755
C	-2.8153097	0.4166449	4.9643538
C	-2.5193347	-1.9338969	4.4504692
C	-3.2585369	0.0843313	6.2507902
H	-2.7611027	1.4701443	4.6652825
C	-2.9629259	-2.2662895	5.7369981
H	-2.2148602	-2.7282498	3.7627775
C	-3.3354155	-1.2605728	6.6410249
H	-3.5463658	0.8775458	6.9489186
H	-3.0168888	-3.3188634	6.0349898
H	-3.6830990	-1.5238804	7.6457045
C	-2.3132064	-1.0460309	1.5661447
C	-1.9907040	-0.1864144	2.6718955
H	-0.7039146	1.3459369	-0.9802393
H	-0.6098808	-1.5052382	-1.7332293
O	0.2153851	2.3660684	2.4429902
C	0.2264150	1.3412672	3.1530943
C	0.5075733	1.5200557	4.6516457
C	-0.0881577	0.0118333	2.6487173
C	0.9051046	0.3036224	5.4109443
C	0.4456488	-1.1688865	3.3780213
H	0.0328790	-0.0442904	1.5608508
C	1.3177193	0.4359395	6.7510199
C	0.8975754	-0.9754506	4.7966836
C	1.7298033	-0.6873476	7.4734355
H	1.3084740	1.4354472	7.1974918
C	1.3161532	-2.0970883	5.5332396
C	1.7329481	-1.9545030	6.8610999
H	2.0537877	-0.5817306	8.5143278
H	1.3039892	-3.0700873	5.0323434
H	2.0631482	-2.8323918	7.4268698
O	0.4353393	2.6370745	5.1562893
O	0.4675860	-2.2863330	2.8551324
H	-0.3492329	3.1401208	0.9258528
H	1.4896875	-3.2137822	-0.0293627
N	-1.2492673	-3.7681659	-3.7772194
C	-0.3192691	-4.2877993	-4.7759884
H	-0.5504561	-3.8275304	-5.7532945
H	-0.3670051	-5.3941311	-4.9089876
H	0.7149776	-4.0061330	-4.5175290
C	-2.6425177	-3.9982110	-4.1366624
H	-2.8430627	-3.5569194	-5.1297644
H	-3.2998278	-3.5041858	-3.4009408
H	-2.9295231	-5.0760580	-4.1913481

RSO2

Energy = -4023.990593650 v_i -313.929993

C	2.6901484	-1.2470017	-1.1318311
C	3.0718255	-0.5967863	0.0596276
C	3.4055337	-2.3805951	-1.5680020
C	4.1693600	-1.0695650	0.7921207
H	2.4941251	0.2561346	0.4265159
C	4.4824778	-2.8582773	-0.8101491
H	3.1107240	-2.8717895	-2.4997816
C	4.8767936	-2.2035266	0.3662847
H	4.4586153	-0.5537700	1.7147771
H	5.0296647	-3.7419708	-1.1564733
H	5.7287849	-2.5741719	0.9461978
C	-0.8068695	0.4794828	-4.4216590
C	-2.1644655	0.1027852	-4.3670658
C	-0.3510484	1.3271414	-5.4525052
C	-3.0503128	0.5779896	-5.3435470
H	-2.5122809	-0.5563169	-3.5614180
C	-1.2566507	1.8140681	-6.4044513
H	0.7097509	1.5911150	-5.4976220
C	-2.6078886	1.4403627	-6.3583776
H	-4.1043852	0.2816760	-5.3009954
H	-0.8958889	2.4760628	-7.1994544
H	-3.3104791	1.8145874	-7.1105431
N	0.0953094	0.0101967	-3.4311601
N	1.5887207	-0.7484525	-1.8820744
S	2.8371419	1.6555696	-3.5782523
S	0.7780531	-3.0455965	-4.0152774
C	0.5937808	3.2672578	-1.6308465
C	0.7792309	4.1324714	-0.3607262
C	-0.7839210	3.5985732	-2.2507210
H	1.3778905	3.5441008	-2.3609843
C	0.7328526	5.6245022	-0.7316087
H	-0.0274458	3.9152812	0.3601303
C	-0.9147355	5.0895691	-2.5934974
H	-1.5743897	3.2890434	-1.5406790
H	-0.9109166	2.9863504	-3.1592499
C	-0.6263775	5.9690179	-1.3702977
H	0.8746513	6.2506786	0.1646332
H	1.5487342	5.8651684	-1.4404660
H	-1.9251723	5.2996101	-2.9871289
H	-0.2016978	5.3389753	-3.4030975
H	-0.6400455	7.0392740	-1.6432832
H	-1.4202171	5.8219381	-0.6134450
C	-1.5494739	-3.2555174	-1.5736943
C	-3.0528208	-3.0571958	-1.2413348
C	-0.8374585	-4.0832252	-0.4787319
H	-1.4535881	-3.7964807	-2.5326010
C	-3.7251524	-4.4180529	-0.9275093
H	-3.0911482	-2.4476175	-0.3175587
C	-1.5321806	-5.4213847	-0.1914493
H	-0.8093975	-3.4847152	0.4521524
C	-3.0034125	-5.1943571	0.1843932

H	-4.7828147	-4.2443390	-0.6554950
H	-3.7302372	-5.0379122	-1.8439952
H	-0.9988446	-5.9539135	0.6167727
H	-1.4777668	-6.0686414	-1.0887392
H	-3.5142167	-6.1581026	0.3657561
H	-3.0560943	-4.6234618	1.1321944
C	3.2715911	3.5054163	-0.3815042
H	3.0867779	2.7431949	-1.1505902
H	4.0637522	3.1629445	0.3006636
H	3.5756401	4.4519031	-0.8545660
C	2.3405777	4.6850877	1.5588912
H	1.4117172	4.8788690	2.1101937
H	2.7788770	5.6096964	1.1581722
H	3.0654257	4.1857849	2.2188067
N	2.0371721	3.7404387	0.4326544
N	0.7701826	1.8280447	-1.3759984
N	-0.9027268	-1.9481360	-1.7558412
P	0.3354711	-1.5939153	-2.7994198
P	1.3804365	0.8077098	-2.5868614
O	-1.6840448	1.0649015	2.0415255
O	-1.2911178	0.3966364	-0.0346502
N	-1.1402337	0.2537988	1.2352965
H	0.1912794	-1.3375026	1.0019100
H	-1.2919756	-0.6439128	3.6267489
C	0.2117029	-2.2414384	3.6745678
C	-0.4000784	-2.8747133	4.7769384
C	1.3526020	-2.8425155	3.0957185
C	0.0970392	-4.0869226	5.2730285
H	-1.2763202	-2.4133490	5.2435778
C	1.8501785	-4.0510450	3.5950713
H	1.8608929	-2.3584100	2.2552595
C	1.2220982	-4.6796570	4.6830589
H	-0.3939522	-4.5665032	6.1262234
H	2.7338899	-4.5032272	3.1326485
H	1.6131781	-5.6259139	5.0719507
C	-0.3827075	-0.7720038	1.7337698
C	-0.3649958	-0.9762610	3.1420237
H	-0.0072346	1.3811156	-0.8374809
H	-1.1650980	-1.1824373	-1.1050010
O	1.3747466	1.6502031	2.0444526
C	0.6687390	1.6123836	3.0866181
C	-0.3083951	2.7418105	3.3869335
C	0.7475339	0.4817604	3.9890171
C	-1.1313632	2.6339057	4.6247242
C	0.1724478	0.5771849	5.3513761
H	1.6934428	-0.0680263	3.9371775
C	-2.1208625	3.6027754	4.8748113
C	-0.8987365	1.6068900	5.5719366
C	-2.8824366	3.5444851	6.0467049
H	-2.2729298	4.3904912	4.1305679
C	-1.6538901	1.5693135	6.7570163
C	-2.6467241	2.5282141	6.9906762
H	-3.6597918	4.2934822	6.2319083
H	-1.4349586	0.7768688	7.4797379
H	-3.2381413	2.4902263	7.9118191
O	-0.3460935	3.7491754	2.6733278

O	0.5072457	-0.1936671	6.2547572
H	1.7464723	2.8404001	0.9352943
N	-3.7436818	-2.2564627	-2.2701537
C	-4.0732693	-2.9635240	-3.5035455
H	-4.3766510	-2.2306604	-4.2712023
H	-4.9037286	-3.6998592	-3.3971240
H	-3.1858962	-3.4937117	-3.8872113
C	-4.8624147	-1.4787592	-1.7511724
H	-5.2375053	-0.8066752	-2.5432688
H	-4.5186496	-0.8527759	-0.9097832
H	-5.7255015	-2.0953314	-1.4014375
H	0.2090492	-4.2430080	-0.7944346

ProS

SRO1O2

Energy = -4023.994381312 v_i -292.35006

C	2.4107333	-0.3490703	-2.7381591
C	3.4890198	0.4993550	-3.0697639
C	2.6448632	-1.7377085	-2.6245517
C	4.7730463	-0.0307291	-3.2512597
H	3.3039967	1.5655618	-3.2281669
C	3.9290330	-2.2549559	-2.8329149
H	1.8142159	-2.4028183	-2.3761925
C	5.0061606	-1.4085483	-3.1352653
H	5.5953719	0.6447613	-3.5131056
H	4.0856092	-3.3370174	-2.7555877
H	6.0088178	-1.8185794	-3.2939844
C	-2.2681848	1.5489044	-3.1008462
C	-3.0891051	2.1629611	-2.1367106
C	-2.6433029	1.5611224	-4.4582375
C	-4.2731158	2.8000179	-2.5343291
H	-2.7967072	2.1153921	-1.0841145
C	-3.8403409	2.1801200	-4.8420057
H	-1.9911858	1.0803708	-5.1925000
C	-4.6531826	2.8077226	-3.8850962
H	-4.9079944	3.2817397	-1.7824845
H	-4.1317447	2.1851650	-5.8979587
H	-5.5816740	3.3013534	-4.1920745
N	-1.0322547	0.9577415	-2.7008343
N	1.1029840	0.1534352	-2.5539397
S	1.0758395	3.1467740	-3.7082629
S	-0.6571926	-1.5729536	-4.6037708
C	0.6969779	3.6877177	-0.4797470
C	1.4899960	3.8417325	0.8385922
C	-0.6593273	4.4155468	-0.3435237
H	1.2527845	4.1796919	-1.2998601
C	1.7158398	5.3264664	1.1686558
H	0.9302433	3.3557794	1.6555125
C	-0.4767353	5.8977812	0.0149332
H	-1.2640097	3.9006358	0.4258919
H	-1.1912673	4.3138974	-1.3041718

C	0.3645412	6.0583189	1.2888913
H	2.2707874	5.4346481	2.1161240
H	2.3204848	5.8041639	0.3732952
H	-1.4617234	6.3808366	0.1437135
H	0.0235027	6.4183434	-0.8245305
H	0.5466996	7.1252123	1.5091605
H	-0.1898975	5.6441315	2.1529787
C	-1.4471414	-2.8620765	-1.5268541
C	-2.8904366	-2.6033114	-1.0116859
C	-0.7193835	-3.9027130	-0.6470140
H	-1.4953736	-3.2377862	-2.5657488
C	-3.6626858	-3.9371945	-0.8539814
H	-2.7793632	-2.1600632	-0.0029074
C	-1.5140397	-5.2073346	-0.4868600
H	-0.5445157	-3.4538993	0.3500680
C	-2.9242066	-4.9309968	0.0552708
H	-4.6755462	-3.7301545	-0.4612938
H	-3.7970231	-4.3991530	-1.8506838
H	-0.9670377	-5.8984661	0.1807290
H	-1.5947184	-5.7141948	-1.4685788
H	-3.4986886	-5.8719583	0.1389845
H	-2.8490828	-4.5122864	1.0784868
C	3.6333088	3.2271080	-0.4200318
H	3.0315784	3.0294866	-1.3165817
H	4.4663624	2.5109011	-0.3738526
H	4.0185489	4.2577893	-0.4367480
C	3.6176737	3.1979004	2.0431663
H	2.9576161	3.0923120	2.9138501
H	4.1172710	4.1769041	2.0281901
H	4.3667944	2.3926258	2.0508519
N	2.7918350	3.0297356	0.8027321
N	0.5669455	2.2740286	-0.8524857
N	-0.6428236	-1.6340514	-1.5672291
P	-0.4159395	-0.6664042	-2.9005195
P	0.4764247	1.7645045	-2.4628384
C	-1.3174481	0.2812272	3.0163170
H	-0.0680621	1.7337445	-0.2189565
H	-0.4716429	-1.1860971	-0.6483091
O	1.2964570	1.4635920	2.9862728
C	1.5720489	0.2488846	3.0722626
C	2.3690779	-0.4042739	1.9506851
C	1.1097403	-0.5613960	4.1837596
C	2.6628482	-1.8551930	2.0451629
C	1.6151157	-1.9370217	4.3706053
C	3.3433772	-2.4949523	0.9915516
C	2.2922655	-2.5935671	3.1979693
C	3.6387690	-3.8599770	1.0745526
H	3.6212533	-1.9059922	0.1126793
C	2.6074746	-3.9600525	3.2750723
C	3.2712633	-4.5931210	2.2167609
H	4.1596463	-4.3554122	0.2482061
H	2.3261102	-4.4990438	4.1855009
H	3.5098723	-5.6603033	2.2818276
O	2.8323487	0.2930025	1.0335138
O	1.4565553	-2.5542419	5.4288964
H	2.5094688	2.0015385	0.8298980

H	0.2762168	-4.1019847	-1.0818164
N	-3.5779086	-1.5806383	-1.8194221
C	-4.1140483	-2.0312811	-3.1017947
H	-4.4076711	-1.1466514	-3.6932217
H	-5.0090350	-2.6907992	-3.0095623
H	-3.3368493	-2.5627415	-3.6746859
C	-4.5591751	-0.8193848	-1.0547423
H	-5.4263362	-1.4291692	-0.7012044
H	-4.9589499	-0.0055884	-1.6826035
H	-4.0731205	-0.3649795	-0.1743517
H	-1.8734364	1.1560136	3.3492888
C	-0.8859249	-0.7989381	3.8348856
C	-1.4680539	-0.9552061	5.1947598
C	-1.7141205	-2.2479529	5.7028413
C	-1.7867547	0.1586623	6.0034785
C	-2.2835833	-2.4235567	6.9703618
H	-1.4575839	-3.1210737	5.0948338
C	-2.3524799	-0.0173996	7.2704771
H	-1.5777652	1.1712544	5.6410635
C	-2.6063898	-1.3099909	7.7575345
H	-2.4702220	-3.4351351	7.3457599
H	-2.5943249	0.8570222	7.8841695
H	-3.0486697	-1.4461594	8.7501802
H	-0.7486634	-1.7330031	3.2781341
H	0.9533912	-0.0048684	5.1138273
N	-0.9700433	0.2858812	1.6932626
O	-0.2050170	-0.6489665	1.2609222
O	-1.3783965	1.2062133	0.9168414

SRO2 = TS14a

Energy = -4023.994593292 v_i -297.619995

C	1.2094406	-2.9919034	-1.3294191
C	1.9437166	-3.9461982	-2.0595058
C	0.8220407	-3.2567440	-0.0031994
C	2.2933453	-5.1611233	-1.4561929
H	2.2252362	-3.7255123	-3.0927263
C	1.1557880	-4.4866117	0.5816905
H	0.2653705	-2.4919416	0.5464519
C	1.8941239	-5.4371453	-0.1397151
H	2.8648880	-5.9032214	-2.0241373
H	0.8354660	-4.7023639	1.6066158
H	2.1538755	-6.3963320	0.3216817
C	-0.0220356	0.8194660	-4.4641904
C	-1.3084330	1.3975083	-4.5057042
C	0.9587931	1.2232674	-5.3949702
C	-1.5967548	2.3667304	-5.4759542
H	-2.0745558	1.0828212	-3.7856069
C	0.6595598	2.2137421	-6.3399447
H	1.9442857	0.7483480	-5.3731906
C	-0.6189254	2.7884117	-6.3899534
H	-2.5995374	2.8077014	-5.5075971
H	1.4301762	2.5217914	-7.0553137
H	-0.8529484	3.5532697	-7.1380492
N	0.2838819	-0.1650218	-3.4897826

N	0.8009996	-1.7692950	-1.9438415
S	3.4264300	-0.8287417	-3.5280008
S	-1.2569568	-2.9328820	-4.0592028
C	2.7899910	1.8042057	-1.5086462
C	3.4093828	2.2650056	-0.1694239
C	2.0592235	3.0071767	-2.1495318
H	3.6049682	1.4933155	-2.1906928
C	4.4187678	3.3995355	-0.4127860
H	2.6063583	2.6183182	0.4985112
C	3.0059818	4.1952984	-2.3741738
H	1.2142775	3.2974480	-1.4966395
H	1.6241877	2.6789017	-3.1085127
C	3.7120451	4.6001397	-1.0729094
H	4.8731872	3.7299943	0.5366357
H	5.2378552	3.0456141	-1.0685599
H	2.4463066	5.0523511	-2.7891569
H	3.7636676	3.9168778	-3.1314922
H	4.4500812	5.4004436	-1.2584709
H	2.9695456	5.0095447	-0.3611940
C	-3.1578783	-1.4502520	-1.6491749
C	-4.2762781	-0.3744703	-1.6753674
C	-3.1508618	-2.2098188	-0.3023886
H	-3.3386563	-2.1821623	-2.4568979
C	-5.6421172	-1.0280440	-1.3374465
H	-4.0419302	0.3471086	-0.8667826
C	-4.5174900	-2.8231834	0.0374649
H	-2.8420785	-1.5117515	0.4996384
C	-5.6213121	-1.7567663	0.0149358
H	-6.4303449	-0.2524930	-1.3413357
H	-5.9031915	-1.7520094	-2.1327735
H	-4.4728436	-3.3183833	1.0249572
H	-4.7586982	-3.6126353	-0.7010615
H	-6.6082109	-2.2126300	0.2171535
H	-5.4401762	-1.0228857	0.8251424
C	4.8750861	0.1566211	-0.1530290
H	4.3266847	-0.2384292	-1.0207217
H	5.1549448	-0.6698512	0.5161925
H	5.7758934	0.6961178	-0.4826859
C	4.6782995	1.5324472	1.8854988
H	4.0205868	2.2455409	2.4004370
H	5.6512108	1.9829960	1.6442074
H	4.8179508	0.6408187	2.5124702
N	3.9971487	1.0898696	0.6246055
N	1.9327425	0.6242156	-1.3387874
N	-1.8491105	-0.8494423	-1.9289467
P	-0.6627772	-1.5005184	-2.8918179
P	1.7361323	-0.4968053	-2.5950338
O	-0.6280011	1.2182902	-0.1528513
O	-0.5238212	-0.1488703	1.6037270
N	-0.7840023	0.9884205	1.1004039
C	-1.2371977	1.9869299	1.9203343
H	1.0377732	0.8004413	-0.8276103
H	-1.5237642	-0.1099613	-1.2781640
O	1.6241185	1.9525998	2.3789976
C	1.2148393	1.1047837	3.1995196
C	1.6831265	-0.3378388	3.0602907

C	0.2785258	1.4513177	4.2514694
C	1.1958962	-1.3357354	4.0462997
C	-0.0210157	0.4979820	5.3377894
C	1.5957752	-2.6805742	3.9269042
C	0.3666778	-0.9413305	5.1266346
C	1.1567538	-3.6301792	4.8559119
H	2.2470291	-2.9560906	3.0922631
C	-0.0587197	-1.9013965	6.0597018
C	0.3266578	-3.2405124	5.9223085
H	1.4632147	-4.6769320	4.7547904
H	-0.6839980	-1.5622573	6.8916521
H	-0.0117227	-3.9847040	6.6512668
O	2.5267784	-0.6381597	2.2001704
O	-0.6014806	0.8430317	6.3723861
H	3.1723495	0.5283862	0.9862316
H	-2.3766840	-2.9960628	-0.3530069
N	-4.2635451	0.4187436	-2.9173220
C	-4.5916514	-0.3050126	-4.1429226
H	-4.3948587	0.3529737	-5.0075020
H	-5.6564333	-0.6308038	-4.2066511
H	-3.9432849	-1.1902967	-4.2498975
C	-4.9816145	1.6814865	-2.8056925
H	-6.0908177	1.5757358	-2.7321189
H	-4.7710292	2.3024942	-3.6946774
H	-4.6325216	2.2303072	-1.9132999
H	-1.3306624	2.9658325	1.4530809
C	-1.5124152	1.6730675	3.2781638
C	-2.2552156	2.6498517	4.1165345
C	-3.1831429	2.1828068	5.0706443
C	-2.0645248	4.0439933	3.9870042
C	-3.9172793	3.0810382	5.8551053
H	-3.3295825	1.1051820	5.1940143
C	-2.7952683	4.9403123	4.7734765
H	-1.3285305	4.4278061	3.2716838
C	-3.7274891	4.4618568	5.7084970
H	-4.6359455	2.6996506	6.5881107
H	-2.6359601	6.0179868	4.6600409
H	-4.2987128	5.1646074	6.3245735
H	-1.7887041	0.6225263	3.4261089
H	0.3144418	2.5005528	4.5623100

SSO1

Energy = -4023.973921430 v_i -389.200012

C	2.8888266	0.1167462	-1.1803625
C	3.6977276	1.2717676	-1.2187317
C	3.2612104	-0.9572907	-0.3450106
C	4.8374754	1.3578688	-0.4093448
H	3.4393993	2.0822705	-1.9071417
C	4.4175083	-0.8667589	0.4401621
H	2.6406773	-1.8563287	-0.3222640
C	5.2069968	0.2927973	0.4249505
H	5.4553900	2.2619380	-0.4503024
H	4.6995309	-1.7133421	1.0761396
H	6.1049429	0.3604302	1.0475174

C	-0.8559580	-0.1341332	-4.5866921
C	-2.2585386	-0.2013463	-4.5001636
C	-0.2310492	-0.0787071	-5.8472237
C	-3.0333826	-0.2027286	-5.6683015
H	-2.7198204	-0.2679759	-3.5113380
C	-1.0123758	-0.0867147	-7.0105119
H	0.8594484	-0.0324658	-5.8995296
C	-2.4114457	-0.1450551	-6.9250077
H	-4.1255111	-0.2529032	-5.5968282
H	-0.5237696	-0.0448621	-7.9900031
H	-3.0174448	-0.1478896	-7.8374972
N	-0.0786116	-0.1091065	-3.3879796
N	1.7290828	0.0271971	-1.9858427
S	1.4161032	2.6004439	-3.9592581
S	1.8803546	-2.5084922	-4.0043684
C	-0.9395343	3.1020447	-1.5642220
C	-0.9513567	3.9793259	-0.2927062
C	-2.3952109	2.7151952	-1.9115626
H	-0.5285012	3.6932142	-2.4061837
C	-1.7750050	5.2547006	-0.5198580
H	-1.3902355	3.4045993	0.5414487
C	-3.2824457	3.9545465	-2.1036471
H	-2.7883733	2.0659994	-1.1062483
H	-2.3754363	2.1138496	-2.8357795
C	-3.2261325	4.8780588	-0.8787624
H	-1.7790924	5.8830639	0.3868196
H	-1.3340487	5.8547668	-1.3397481
H	-4.3247451	3.6460469	-2.2993574
H	-2.9446761	4.5123774	-2.9991802
H	-3.8082781	5.8000894	-1.0551088
H	-3.6860691	4.3695970	-0.0103751
C	-0.0635924	-3.5979408	-1.3926339
C	-1.5964952	-3.7986506	-1.2325079
C	0.7084263	-4.2780276	-0.2388047
H	0.2739029	-4.0457549	-2.3453604
C	-1.9323521	-5.3015727	-1.0481346
H	-1.8806481	-3.2809786	-0.2941913
C	0.3434694	-5.7600089	-0.0626900
H	0.4884024	-3.7343057	0.7009466
C	-1.1704846	-5.9323499	0.1269233
H	-3.0232508	-5.4150770	-0.9078338
H	-1.6727908	-5.8474368	-1.9753497
H	0.8956571	-6.1815813	0.7971889
H	0.6673171	-6.3278500	-0.9571286
H	-1.4305127	-7.0029147	0.2201421
H	-1.4802943	-5.4477224	1.0740161
C	1.4486963	4.7115403	-0.8358802
H	1.4938327	3.9826703	-1.6591635
H	2.4363923	4.7979436	-0.3597641
H	1.1289825	5.6941348	-1.2136632
C	0.5152446	5.1611612	1.3999344
H	-0.2494350	4.8300451	2.1144046
H	0.3394839	6.1970081	1.0768278
H	1.5135830	5.0744777	1.8533285
N	0.4779361	4.2506360	0.2082495
N	-0.0564419	1.9420753	-1.3983885

N	0.2825823	-2.1715017	-1.4514510
P	0.9584745	-1.3631561	-2.7359638
P	0.7518426	1.2496677	-2.7069355
O	-0.6009967	-0.1087831	0.4067490
O	-2.6130319	0.4568688	1.1432857
N	-1.4821637	-0.0400472	1.3528072
H	-1.9632826	-0.4775001	3.3163074
C	-1.1449712	-0.5158458	2.5991972
C	0.1653161	-1.0548097	2.8341482
H	-0.3863773	1.2247702	-0.7041178
H	-0.1617391	-1.5595904	-0.7422859
O	1.3765929	2.1066688	1.7537097
C	0.9160079	1.5516985	2.7630595
C	-0.3776075	2.1116428	3.4072467
C	1.4919462	0.3738449	3.4002559
C	-0.6116013	1.7834178	4.8332080
C	1.4777346	0.3221445	4.8758733
H	2.4258422	0.0196022	2.9489609
C	-1.7182353	2.3531643	5.5007211
C	0.3096942	0.9775623	5.5529119
C	-1.9078749	2.1241408	6.8640020
H	-2.4083279	2.9735257	4.9203261
C	0.1133155	0.7666064	6.9302294
C	-0.9859938	1.3336209	7.5808889
H	-2.7687792	2.5631364	7.3794361
H	0.8442890	0.1456636	7.4575985
H	-1.1338990	1.1636598	8.6530071
O	-1.0911792	2.8962826	2.7771165
O	2.3288585	-0.2924697	5.5262133
H	0.8027222	3.3177759	0.5881047
H	1.7901518	-4.1657499	-0.4326467
N	-2.3551848	-3.1238895	-2.2981798
C	-2.3068644	-3.7621789	-3.6100202
H	-2.7433903	-3.0781387	-4.3576908
H	-2.8645806	-4.7264093	-3.6683807
H	-1.2607206	-3.9465053	-3.9068134
C	-3.7076549	-2.7565173	-1.8995495
H	-4.1670870	-2.1389794	-2.6918146
H	-3.6713852	-2.1534871	-0.9752046
H	-4.3891849	-3.6251023	-1.7254422
C	0.3581473	-2.1355373	3.8383238
C	1.5270993	-2.9282367	3.7829522
C	-0.5922775	-2.4147430	4.8458016
C	1.7317418	-3.9734870	4.6891537
H	2.2803376	-2.7165082	3.0166239
C	-0.3857162	-3.4596108	5.7552226
H	-1.5080687	-1.8207540	4.9140011
C	0.7750427	-4.2433031	5.6802060
H	2.6421451	-4.5784458	4.6252798
H	-1.1382620	-3.6648564	6.5240106
H	0.9340143	-5.0612436	6.3908925
H	0.7150343	-1.2058565	1.8990973

SSO1O2

Energy = -4023.989169910 v_i -261.390015

C	2.7480315	0.0150889	-0.3073977
C	3.5554185	1.1126067	0.0606776
C	2.8062896	-1.1657261	0.4615376
C	4.3785827	1.0309664	1.1915992
H	3.5549164	2.0124702	-0.5606468
C	3.6500096	-1.2418699	1.5761341
H	2.1824350	-2.0180213	0.1864664
C	4.4342459	-0.1426703	1.9574349
H	4.9993306	1.8925414	1.4621733
H	3.6815512	-2.1701691	2.1566047
H	5.0869963	-0.2041046	2.8342397
C	0.1211143	0.2802117	-4.6724882
C	-0.9552068	-0.5834302	-4.9589519
C	0.5262323	1.2423787	-5.6206448
C	-1.6160879	-0.4737745	-6.1900871
H	-1.2608614	-1.3309630	-4.2150158
C	-0.1619262	1.3538060	-6.8363448
H	1.3789679	1.8897426	-5.3962679
C	-1.2326960	0.4963467	-7.1288917
H	-2.4519953	-1.1462913	-6.4119325
H	0.1559333	2.1060124	-7.5667574
H	-1.7622475	0.5798922	-8.0839433
N	0.7862827	0.1834255	-3.4211129
N	1.8847971	0.0764006	-1.4277670
S	2.5260677	2.8335143	-2.9730334
S	2.8234202	-2.2653720	-3.4215701
C	-0.5138728	3.4178830	-1.7628079
C	-1.0726122	4.0284879	-0.4586058
C	-1.6126588	3.4440067	-2.8494541
H	0.3369957	4.0275124	-2.1220775
C	-1.5651005	5.4659346	-0.6859171
H	-1.9110095	3.3904575	-0.1229980
C	-2.1429073	4.8656489	-3.0882537
H	-2.4341741	2.7706940	-2.5411606
H	-1.1838467	3.0282943	-3.7774196
C	-2.6485339	5.4937923	-1.7814603
H	-1.9762620	5.8889929	0.2470378
H	-0.7174342	6.1080943	-0.9941201
H	-2.9515337	4.8464398	-3.8403531
H	-1.3340045	5.4943329	-3.5090068
H	-2.9719831	6.5369211	-1.9455107
H	-3.5357691	4.9374463	-1.4228696
C	0.0733909	-3.5579919	-1.9190188
C	-1.4314431	-3.9329727	-2.0079252
C	0.7358858	-4.1857995	-0.6699249
H	0.6037603	-3.9263791	-2.8161668
C	-1.6331865	-5.4620533	-1.8559739
H	-1.9200656	-3.4429108	-1.1458973
C	0.5073142	-5.7003909	-0.5567342
H	0.3305954	-3.6906604	0.2333421
C	-0.9931728	-6.0214532	-0.5770402
H	-2.7163287	-5.6832029	-1.8789426

H	-1.1846068	-5.9779766	-2.7261139
H	0.9779166	-6.0799485	0.3683583
H	1.0042921	-6.2168704	-1.4014638
H	-1.1620219	-7.1125375	-0.5146103
H	-1.4771831	-5.5719753	0.3120428
C	1.3028721	4.4626259	0.4077242
H	1.7123641	3.9854122	-0.4956910
H	1.9353099	4.2225405	1.2747448
H	1.2418007	5.5530604	0.2746663
C	-0.5957532	4.4423841	1.9892068
H	-1.6374058	4.1074572	2.0999280
H	-0.5490496	5.5408238	1.9936795
H	0.0189218	4.0069785	2.7924731
N	-0.0638617	3.9149092	0.6883585
N	0.0122353	2.0745171	-1.5051409
N	0.2122738	-2.0883313	-1.9049877
P	1.4417134	-1.2037938	-2.5745814
P	1.3194079	1.4178319	-2.3468907
O	-1.6686662	-1.1525225	0.0584676
O	-2.4122164	0.8606645	-0.4939962
N	-2.1980518	-0.0379440	0.3794374
H	-2.9202082	1.1799446	1.8963120
C	-2.4903642	0.1993038	1.6976194
C	-2.0344360	-0.7125357	2.7049921
H	-0.7293958	1.4040703	-1.2145304
H	-0.5020179	-1.5807645	-1.3489799
O	-0.0030082	1.4187386	1.6896820
C	0.2723244	0.6696849	2.6555140
C	0.8949739	1.2550430	3.9150015
C	-0.1277111	-0.7207210	2.6230198
C	1.2898085	0.3039710	4.9904228
C	0.3638538	-1.6624409	3.6562003
H	-0.1383287	-1.1463225	1.6074923
C	1.9310427	0.8002097	6.1419734
C	1.0342511	-1.0869109	4.8714316
C	2.3169316	-0.0720313	7.1648359
H	2.1150378	1.8771705	6.2062355
C	1.4264084	-1.9534389	5.9051759
C	2.0652634	-1.4508791	7.0451202
H	2.8167496	0.3178186	8.0579982
H	1.2187267	-3.0210640	5.7822729
H	2.3713358	-2.1333696	7.8452486
O	1.0783501	2.4722429	4.0130926
O	0.2028008	-2.8811978	3.5396727
H	0.0591518	2.8785242	0.8828146
H	1.8167025	-3.9643037	-0.7139509
N	-2.0722461	-3.3573645	-3.2083271
C	-1.8358873	-4.0775459	-4.4548330
H	-2.1587365	-3.4510474	-5.3045880
H	-2.3807740	-5.0470718	-4.5298547
H	-0.7583022	-4.2755695	-4.5817560
C	-3.4773713	-3.0193744	-3.0119635
H	-3.8473632	-2.4670899	-3.8945346
H	-3.5757476	-2.3621615	-2.1318047
H	-4.1439487	-3.9045202	-2.8702156
H	-2.0824892	-1.7678395	2.4059603

C	-2.4817666	-0.4744802	4.1130765
C	-2.8163480	-1.5753973	4.9290220
C	-2.5671967	0.8212742	4.6663925
C	-3.2407804	-1.3864296	6.2510639
H	-2.7401944	-2.5872617	4.5173202
C	-2.9891484	1.0102163	5.9882497
H	-2.2922313	1.6912712	4.0596833
C	-3.3308963	-0.0933322	6.7848975
H	-3.5019711	-2.2541336	6.8661764
H	-3.0486765	2.0234323	6.4002389
H	-3.6615249	0.0550305	7.8184899

Crystal Data

Catalyst 6

Identification code	hk586
Empirical formula	C85 H96 N10 O4 P2
Moiety formula	2(C32 H36 N5 O2 P), 3(C7 H8)
Formula weight	1383.66
Temperature	100(2) K
Wavelength	0.71073 A
Crystal system, space group	Monoclinic, P21
Unit cell dimensions	a = 13.2005(7) A alpha = 90 deg.<br>b = 14.9724(6) A beta = 103.532(2) deg.<br>c = 19.2057(10) A gamma = 90 deg.
Volume	3690.5(3) A^3
Z, Calculated density	2, 1.245 Mg/m^3
Absorption coefficient	0.118 mm^-1
F(000)	1476
Crystal size	.5 x .5 x .2 mm
Theta range for data collection	1.09 to 27.00 deg.
Limiting indices	-16<=h<=12, -17<=k<=19, -16<=l<=24
Reflections collected / unique	19516 / 15009 [R(int) = 0.0659]
Reflection observed [I>2sigma(I)]	7457
Completeness to theta = 27.00	99.9 %
Absorption correction	None
Refinement method	Full-matrix least-squares on F^2
Data / restraints / parameters	15009 / 1 / 943
Goodness-of-fit on F^2	0.879
Final R indices [I>2sigma(I)]	R1 = 0.0600, wR2 = 0.0917
R indices (all data)	R1 = 0.1607, wR2 = 0.1138
Absolute structure parameter	-0.06(8)
Largest diff. peak and hole	0.314 and -0.354 e.A^-3

Table 2. Atomic coordinates (x 10^4) and equivalent isotropic displacement parameters (A^2 x 10^3) for z4_b.
U(eq) is defined as one third of the trace of the orthogonalized Uij tensor.

	x	y	z	U(eq)
P(1)	6791(1)	3444(1)	6387(1)	21(1)
O(1)	7902(2)	3474(2)	6794(1)	24(1)
O(2)	10052(2)	1308(2)	7703(2)	33(1)
N(1)	6040(2)	3603(2)	6948(2)	21(1)
N(2)	6496(3)	4179(2)	5736(2)	23(1)
N(3)	6380(3)	2505(2)	5970(2)	19(1)
N(4)	6390(2)	3098(2)	4476(2)	21(1)
N(5)	6985(3)	-815(2)	6069(2)	27(1)
C(1)	4973(3)	3860(2)	6769(2)	20(1)
C(2)	4621(3)	4406(3)	7251(2)	26(1)
C(3)	3582(3)	4659(3)	7102(2)	31(1)
C(4)	2886(3)	4370(3)	6490(3)	30(1)
C(5)	3232(3)	3827(3)	6003(2)	29(1)
C(6)	4270(3)	3573(2)	6144(2)	24(1)
C(7)	6737(3)	5090(3)	5790(2)	23(1)
C(8)	6745(3)	5579(3)	5175(2)	31(1)
C(9)	6941(3)	6492(3)	5208(3)	36(1)
C(10)	7136(3)	6929(3)	5854(3)	35(1)
C(11)	7130(3)	6455(3)	6473(3)	34(1)
C(12)	6929(3)	5543(3)	6440(2)	29(1)
C(13)	6903(3)	2002(2)	5486(2)	17(1)
C(14)	6351(3)	2142(2)	4694(2)	23(1)
C(15)	5655(3)	3199(3)	3761(2)	28(1)
C(16)	5974(3)	2616(3)	3179(2)	28(1)
C(17)	6973(3)	2105(3)	3546(2)	26(1)
C(18)	6803(3)	1543(3)	4180(2)	27(1)
C(19)	7821(3)	2792(3)	3848(2)	31(1)
C(20)	7445(3)	3362(3)	4408(2)	25(1)
C(21)	5107(4)	2027(3)	2810(3)	42(1)
C(22)	4597(4)	2099(4)	2146(3)	68(2)
C(23)	6943(3)	1027(2)	5699(2)	17(1)
C(24)	7757(3)	688(2)	6272(2)	18(1)
C(25)	7755(3)	-238(2)	6414(2)	25(1)
C(26)	6230(3)	-465(3)	5575(2)	31(1)
C(27)	6178(3)	436(2)	5372(2)	21(1)
C(28)	8565(3)	-604(3)	6950(2)	32(1)
C(29)	9310(3)	-78(3)	7346(2)	31(1)
C(30)	9298(3)	861(3)	7234(2)	27(1)
C(31)	10047(3)	2263(3)	7646(2)	37(1)
C(32)	8535(3)	1231(3)	6697(2)	24(1)
P(2)	1933(1)	8853(1)	1243(1)	21(1)
O(3)	3063(2)	8837(2)	1590(1)	23(1)
O(4)	4770(2)	6545(2)	2793(2)	31(1)
N(6)	1255(2)	9136(2)	1825(2)	18(1)
N(7)	1584(3)	9541(2)	558(2)	20(1)
N(8)	1506(3)	7873(2)	910(2)	21(1)
N(9)	1750(2)	8312(2)	-548(2)	21(1)
N(10)	1596(3)	4514(2)	1155(2)	26(1)
C(33)	164(3)	9294(2)	1710(2)	18(1)
C(34)	-190(3)	9648(3)	2279(2)	27(1)
C(35)	-1252(3)	9787(3)	2205(2)	32(1)
C(36)	-1957(3)	9591(3)	1571(2)	29(1)

C(37)	-1599(3)	9263(2)	1004(2)	27(1)
C(38)	-538(3)	9109(2)	1073(2)	24(1)
C(39)	1681(3)	10479(3)	526(2)	21(1)
C(40)	1732(3)	11026(3)	1117(2)	30(1)
C(41)	1766(3)	11948(3)	1050(3)	31(1)
C(42)	1752(3)	12328(3)	398(3)	35(1)
C(43)	1715(3)	11792(3)	-199(3)	33(1)
C(44)	1682(3)	10869(3)	-127(2)	27(1)
C(45)	1995(3)	7267(2)	495(2)	22(1)
C(46)	1592(3)	7377(2)	-315(2)	23(1)
C(47)	1106(3)	8425(3)	-1279(2)	27(1)
C(48)	1358(3)	7721(3)	-1815(2)	23(1)
C(49)	2310(3)	7183(3)	-1403(2)	29(1)
C(50)	2056(3)	6702(3)	-763(2)	28(1)
C(51)	3191(3)	7848(3)	-1110(2)	31(1)
C(52)	2852(3)	8458(3)	-562(2)	28(1)
C(53)	439(3)	7154(3)	-2140(3)	29(1)
C(54)	-24(3)	7131(3)	-2828(3)	39(1)
C(55)	1856(3)	6309(2)	738(2)	19(1)
C(56)	2568(3)	5937(2)	1345(2)	20(1)
C(57)	2428(3)	5030(3)	1511(2)	22(1)
C(58)	931(3)	4919(3)	649(2)	27(1)
C(59)	1025(3)	5792(3)	409(2)	24(1)
C(60)	3149(3)	4612(3)	2074(2)	27(1)
C(61)	3941(3)	5089(3)	2495(2)	31(1)
C(62)	4039(3)	6013(3)	2363(2)	24(1)
C(63)	5499(3)	6132(3)	3373(2)	32(1)
C(64)	3377(3)	6419(3)	1796(2)	23(1)
C(65)	491(3)	4403(3)	4865(3)	33(1)
C(66)	16(3)	4492(3)	5439(3)	32(1)
C(67)	-190(3)	3726(3)	5787(2)	32(1)
C(68)	69(3)	2891(3)	5564(2)	32(1)
C(69)	550(3)	2820(3)	5007(2)	32(1)
C(70)	748(3)	3571(3)	4654(2)	33(1)
C(71)	-236(4)	5402(3)	5687(3)	54(2)
C(72)	7146(4)	5738(3)	2130(3)	41(1)
C(73)	7261(3)	6604(3)	2411(3)	33(1)
C(74)	7868(3)	6726(3)	3091(3)	39(1)
C(75)	8357(3)	6016(3)	3491(3)	44(1)
C(76)	8260(3)	5168(3)	3219(3)	40(1)
C(77)	7648(3)	5029(3)	2541(3)	38(1)
C(78)	6714(4)	7368(3)	1968(3)	58(2)
C(79)	5812(4)	8965(4)	-438(4)	66(2)
C(80)	5727(4)	8554(4)	160(3)	66(2)
C(81)	5257(4)	9041(5)	640(3)	81(2)
C(82)	4892(5)	9868(7)	522(5)	99(3)
C(83)	5012(5)	10314(5)	-121(5)	105(3)
C(84)	5465(3)	9857(3)	-557(3)	81(2)
C(85)	4528(3)	10542(3)	898(3)	60(3)
C(86)	4650(3)	11167(3)	-39(3)	76(4)

Table 3. Anisotropic displacement parameters (A^2 x 10^3) for z4_b.
The anisotropic displacement factor exponent takes the form:
-2 pi^2 [h^2 a*^2 U11 + ... + 2 h k a* b* U12]

	U11	U22	U33	U23	U13	U12
P(1)	25(1)	20(1)	17(1)	-2(1)	4(1)	2(1)

O(1)	22(2)	26(2)	19(2)	-5(1)	-4(1)	0(1)
O(2)	33(2)	35(2)	26(2)	1(2)	-1(2)	10(2)
N(1)	21(2)	30(2)	12(2)	-2(2)	2(2)	7(2)
N(2)	34(2)	23(2)	11(3)	-1(2)	5(2)	-3(2)
N(3)	27(2)	20(2)	14(2)	-2(2)	12(2)	-2(2)
N(4)	27(2)	21(2)	14(2)	8(2)	1(2)	2(2)
N(5)	42(2)	22(2)	21(2)	0(2)	14(2)	-1(2)
C(1)	21(2)	19(2)	19(3)	7(2)	5(2)	-0(2)
C(2)	24(3)	34(3)	21(3)	0(2)	5(2)	0(2)
C(3)	36(3)	34(3)	27(3)	0(2)	14(3)	10(2)
C(4)	21(3)	35(3)	35(3)	13(2)	8(2)	5(2)
C(5)	22(3)	31(3)	29(3)	1(2)	-5(2)	-5(2)
C(6)	25(2)	27(3)	19(3)	-4(2)	3(2)	-3(2)
C(7)	16(2)	21(3)	31(3)	0(2)	2(2)	1(2)
C(8)	38(3)	24(3)	28(3)	-2(2)	0(2)	-3(2)
C(9)	37(3)	29(3)	36(4)	6(3)	-6(3)	-1(2)
C(10)	34(3)	23(3)	43(4)	-4(3)	4(3)	-5(2)
C(11)	38(3)	26(3)	38(4)	-15(3)	7(3)	-10(2)
C(12)	33(3)	31(3)	27(3)	-5(2)	15(2)	-7(2)
C(13)	25(2)	15(2)	12(3)	-2(2)	6(2)	2(2)
C(14)	31(3)	16(2)	19(3)	-1(2)	4(2)	1(2)
C(15)	33(3)	28(3)	20(3)	6(2)	2(2)	9(2)
C(16)	36(3)	33(3)	14(3)	1(2)	7(2)	1(2)
C(17)	37(3)	23(2)	19(3)	-3(2)	7(2)	1(2)
C(18)	35(3)	26(2)	21(3)	0(2)	7(2)	3(2)
C(19)	28(3)	36(3)	27(3)	0(2)	0(2)	0(2)
C(20)	33(3)	23(2)	20(3)	5(2)	6(2)	-3(2)
C(21)	41(3)	49(3)	37(4)	-5(3)	10(3)	0(3)
C(22)	55(4)	112(5)	31(4)	-2(4)	-4(3)	-10(4)
C(23)	23(2)	19(2)	12(3)	1(2)	9(2)	2(2)
C(24)	27(3)	17(2)	12(3)	3(2)	9(2)	5(2)
C(25)	36(3)	20(3)	22(3)	1(2)	12(2)	4(2)
C(26)	39(3)	30(3)	26(3)	-6(2)	14(3)	-8(2)
C(27)	28(3)	19(2)	16(3)	-2(2)	6(2)	-1(2)
C(28)	48(3)	21(3)	27(3)	4(2)	12(3)	9(2)
C(29)	37(3)	37(3)	19(3)	4(2)	8(2)	15(2)
C(30)	32(3)	32(3)	21(3)	-1(2)	12(2)	4(2)
C(31)	39(3)	28(3)	40(3)	-6(2)	2(3)	11(2)
C(32)	30(3)	23(2)	19(3)	6(2)	9(2)	9(2)
P(2)	27(1)	20(1)	16(1)	-0(1)	4(1)	1(1)
O(3)	19(2)	31(2)	19(2)	2(1)	1(1)	-2(1)
O(4)	31(2)	26(2)	27(2)	2(2)	-12(2)	2(1)
N(6)	19(2)	26(2)	9(2)	-0(2)	0(2)	2(2)
N(7)	30(2)	19(2)	12(2)	0(2)	4(2)	4(2)
N(8)	25(2)	18(2)	22(2)	-4(2)	13(2)	-5(2)
N(9)	23(2)	22(2)	15(2)	4(2)	-1(2)	-2(2)
N(10)	34(2)	22(2)	23(2)	-2(2)	7(2)	-6(2)
C(33)	22(2)	17(2)	15(3)	6(2)	3(2)	3(2)
C(34)	30(3)	30(3)	20(3)	0(2)	5(2)	1(2)
C(35)	33(3)	39(3)	29(3)	-4(2)	17(2)	4(2)
C(36)	22(3)	35(3)	29(3)	0(2)	3(2)	-2(2)
C(37)	28(3)	22(2)	27(3)	-1(2)	0(2)	-2(2)
C(38)	27(3)	22(2)	23(3)	-1(2)	8(2)	2(2)
C(39)	24(2)	17(2)	20(3)	2(2)	4(2)	-0(2)
C(40)	31(3)	35(3)	24(3)	3(2)	3(2)	0(2)
C(41)	39(3)	18(3)	38(4)	-6(2)	10(3)	-2(2)
C(42)	38(3)	18(3)	43(4)	5(3)	1(3)	-4(2)
C(43)	38(3)	29(3)	30(3)	13(2)	5(3)	1(2)
C(44)	39(3)	21(2)	22(3)	-1(2)	8(2)	0(2)
C(45)	22(2)	21(2)	22(3)	0(2)	7(2)	3(2)
C(46)	30(3)	24(2)	14(3)	-1(2)	1(2)	0(2)
C(47)	34(3)	29(2)	16(3)	5(2)	1(2)	3(2)

C(48)	37(3)	28(2)	3(2)	2(2)	2(2)	-3(2)
C(49)	43(3)	29(3)	18(3)	-8(2)	15(2)	2(2)
C(50)	37(3)	30(3)	18(3)	3(2)	9(2)	7(2)
C(51)	23(3)	45(3)	25(3)	0(2)	6(2)	4(2)
C(52)	35(3)	28(2)	22(3)	8(2)	5(2)	-3(2)
C(53)	35(3)	26(3)	27(3)	-4(2)	11(2)	-3(2)
C(54)	40(3)	42(3)	34(4)	-6(3)	6(3)	0(2)
C(55)	26(3)	20(2)	12(3)	0(2)	8(2)	2(2)
C(56)	27(3)	19(2)	15(3)	-1(2)	9(2)	3(2)
C(57)	24(3)	24(2)	20(3)	4(2)	7(2)	6(2)
C(58)	30(3)	31(3)	21(3)	-3(2)	5(2)	-13(2)
C(59)	30(3)	26(3)	14(3)	3(2)	2(2)	1(2)
C(60)	33(3)	23(2)	26(3)	0(2)	4(2)	4(2)
C(61)	36(3)	26(3)	28(3)	6(2)	0(2)	6(2)
C(62)	26(3)	27(3)	18(3)	1(2)	2(2)	-2(2)
C(63)	32(3)	43(3)	21(3)	3(2)	6(2)	-1(2)
C(64)	32(3)	22(2)	17(3)	4(2)	7(2)	2(2)
C(65)	35(3)	26(3)	36(3)	9(2)	5(2)	-2(2)
C(66)	29(3)	34(3)	27(3)	-4(2)	-7(2)	4(2)
C(67)	31(3)	42(3)	22(3)	1(2)	6(2)	2(2)
C(68)	33(3)	39(3)	20(3)	5(2)	-1(2)	-6(2)
C(69)	31(3)	27(3)	33(3)	-6(2)	-3(2)	-2(2)
C(70)	32(3)	49(3)	19(3)	1(2)	7(2)	-5(2)
C(71)	66(4)	37(3)	58(4)	4(3)	10(3)	20(3)
C(72)	52(3)	42(3)	31(3)	-4(3)	16(3)	-2(3)
C(73)	36(3)	32(3)	35(4)	8(3)	18(3)	3(2)
C(74)	39(3)	31(3)	54(4)	-4(3)	21(3)	-7(2)
C(75)	37(3)	47(3)	50(4)	5(3)	13(3)	-15(3)
C(76)	33(3)	37(3)	51(4)	1(3)	14(3)	-4(2)
C(77)	48(3)	28(3)	44(4)	-14(3)	22(3)	-5(2)
C(78)	62(4)	49(3)	69(5)	13(3)	23(3)	9(3)
C(79)	46(4)	82(5)	65(5)	-4(4)	1(3)	-17(3)
C(80)	40(3)	92(5)	57(5)	-17(4)	-6(3)	-10(3)
C(81)	46(4)	137(7)	51(5)	-37(5)	-6(3)	11(4)
C(82)	43(4)	143(8)	88(7)	-73(6)	-28(5)	15(5)
C(83)	36(4)	92(6)	164(10)	-65(6)	-22(6)	1(4)
C(84)	38(4)	65(5)	134(7)	23(5)	11(4)	-6(3)
C(85)	39(7)	119(10)	25(7)	21(7)	13(6)	11(7)
C(86)	35(7)	37(7)	125(12)	-25(7)	-46(7)	-9(5)

Table 4. Hydrogen coordinates (x 10^4) and isotropic displacement parameters (A^2 x 10^3) for z4_b.

	x	y	z	U(eq)
H(2)	5089	4604	7677	31
H(3)	3344	5038	7427	37
H(4)	2174	4540	6401	36
H(5)	2760	3634	5577	35
H(6)	4507	3200	5815	29
H(8)	6615	5284	4725	38
H(9)	6940	6814	4782	43
H(10)	7274	7553	5877	41
H(11)	7264	6755	6922	41
H(12)	6923	5225	6867	35
H(13)	7634	2226	5560	20
H(14)	5601	1977	4637	27
H(15A)	5634	3834	3614	33

H(15B)	4945	3028	3799	33
H(16)	6151	3025	2814	33
H(17)	7202	1712	3190	31
H(18A)	7473	1282	4441	33
H(18B)	6314	1048	4001	33
H(19A)	7949	3177	3459	38
H(19B)	8480	2484	4075	38
H(20A)	7942	3297	4879	30
H(20B)	7435	3999	4267	30
H(21)	4908	1558	3083	50
H(22A)	4771	2559	1853	82
H(22B)	4052	1692	1953	82
H(26)	5683	-848	5340	37
H(27)	5611	641	5005	25
H(28)	8587	-1230	7033	38
H(29)	9849	-338	7704	37
H(30A)	10128	2435	7170	55
H(30B)	10624	2510	8012	55
H(30C)	9385	2496	7718	55
H(32)	8533	1856	6612	28
H(34)	290	9795	2715	32
H(35)	-1495	10019	2596	39
H(36)	-2682	9681	1527	35
H(37)	-2078	9141	562	32
H(38)	-299	8876	680	28
H(40)	1745	10767	1570	36
H(41)	1799	12317	1457	38
H(42)	1768	12960	354	42
H(43)	1712	12053	-650	39
H(44)	1660	10500	-533	33
H(45)	2759	7400	614	26
H(46)	823	7269	-425	28
H(47A)	1218	9031	-1451	32
H(47B)	362	8373	-1268	32
H(48)	1571	8052	-2211	28
H(49)	2534	6743	-1730	34
H(50A)	2698	6435	-463	34
H(50B)	1550	6216	-932	34
H(51A)	3337	8211	-1506	37
H(51B)	3834	7522	-878	37
H(52A)	3299	8340	-80	34
H(52B)	2951	9089	-684	34
H(53)	166	6780	-1828	34
H(54A)	226	7495	-3157	47
H(54B)	-607	6751	-2994	47
H(58)	327	4594	420	33
H(59)	513	6025	19	29
H(60)	3083	3993	2161	33
H(61)	4424	4804	2874	37
H(63A)	5120	5832	3690	48
H(63B)	5961	6588	3645	48
H(63C)	5914	5692	3184	48
H(64)	3467	7034	1705	28
H(65)	638	4920	4618	40
H(67)	-511	3772	6179	38
H(68)	-88	2368	5799	38
H(69)	746	2249	4865	38
H(70)	1065	3518	4260	40
H(71A)	-946	5400	5761	82
H(71B)	-185	5848	5323	82
H(71C)	258	5550	6138	82
H(72)	6729	5634	1662	49

H(74)	7952	7310	3290	47
H(75)	8765	6118	3962	53
H(76)	8610	4683	3492	48
H(77)	7566	4440	2352	46
H(78A)	6359	7736	2260	88
H(78B)	6201	7132	1555	88
H(78C)	7225	7733	1800	88
H(79)	6102	8663	-780	80
H(80)	5968	7960	262	79
H(81)	5202	8756	1072	97
H(82)	4561	10173	873	118
H(83)	4759	10907	-185	126
H(84)	5561	10149	-976	97
H(85A)	4019	10294	1143	90
H(85B)	4197	11009	563	90
H(85C)	5113	10799	1251	90
H(86A)	4874	11574	-373	114
H(86B)	4932	11371	454	114
H(86C)	3887	11159	-138	114
H(1X)	6070(30)	2140(20)	6215(19)	11(10)
H(2X)	1570(30)	8980(20)	2320(20)	24(12)
H(3X)	1510(30)	9250(20)	152(19)	12(11)
H(4X)	6340(30)	3980(20)	5383(19)	3(12)
H(5X)	6360(30)	3630(20)	7470(20)	43(14)
H(6X)	990(30)	7750(20)	960(20)	14(13)

Catalyst 7a

Identification code	hk488-vor
Empirical formula	C83 H92 N10 O2 P2
Moiety formula	2(C31 H34 N5 O P), 3(C7 H8)
Formula weight	1323.61
Temperature	100(2) K
Wavelength	0.71073 A
Crystal system, space group	Monoclinic, P21
Unit cell dimensions	a = 13.1487(14) A alpha = 90 deg.<br>b = 14.9968(18) A beta = 103.672(5) deg.<br>c = 18.6279(12) A gamma = 90 deg.
Volume	3569.1(6) A^3
Z, Calculated density	2, 1.232 Mg/m^3
Absorption coefficient	0.117 mm^-1
F(000)	1412
Crystal size	.15 x .1 x .05 mm
Theta range for data collection	1.12 to 25.00 deg.

Limiting indices	-13<=h<=15, -17<=k<=16, -22<=l<=20
Reflections collected / unique	14543 / 10932 [R(int) = 0.0580]
Reflection observed [I>2sigma(I)]	5220
Completeness to theta = 25.00	99.8 %
Absorption correction	None
Refinement method	Full-matrix least-squares on F^2
Data / restraints / parameters	10932 / 7 / 895
Goodness-of-fit on F^2	0.920
Final R indices [I>2sigma(I)]	R1 = 0.0698, wR2 = 0.1167
R indices (all data)	R1 = 0.1781, wR2 = 0.1438
Absolute structure parameter	0.05(13)
Largest diff. peak and hole	0.926 and -0.248 e.A^-3

Table 2. Atomic coordinates (x 10^4) and equivalent isotropic displacement parameters (A^2 x 10^3) for hk488.
U(eq) is defined as one third of the trace of the orthogonalized Uij tensor.

	x	y	z	U(eq)
P(1)	8137(1)	2428(1)	3777(1)	31(1)
O(1)	7014(3)	2525(3)	3439(2)	33(1)
N(1)	8482(4)	1401(4)	4061(3)	32(2)
C(2)	8430(5)	806(4)	5307(3)	31(2)
N(3)	7628(5)	-1871(4)	3527(3)	40(2)
N(4)	8831(4)	2686(4)	3186(2)	31(2)
N(5)	8556(4)	3033(4)	4526(2)	31(1)
C(1)	7928(5)	829(4)	4475(3)	28(2)
N(2)	8503(4)	1704(4)	5637(2)	31(2)
C(3)	9224(5)	1659(5)	6369(3)	34(2)
C(4)	8852(5)	930(5)	6880(3)	45(2)
C(5)	7780(5)	615(5)	6466(3)	36(2)
C(6)	7838(5)	159(4)	5736(3)	37(2)
C(7)	7054(5)	1426(5)	6275(3)	44(2)
C(8)	7485(5)	2024(5)	5751(3)	38(2)
C(9)	9639(6)	194(6)	7096(4)	65(3)
C(10)	10122(7)	-69(7)	7709(5)	100(4)
C(11)	7833(5)	-115(5)	4133(3)	32(2)
C(12)	6995(5)	-311(5)	3500(3)	32(2)
C(13)	6941(6)	-1204(6)	3253(3)	42(2)
C(14)	8429(6)	-1621(5)	4096(3)	41(2)
C(15)	8529(6)	-770(5)	4400(3)	36(2)
C(16)	6095(6)	-1449(5)	2647(3)	46(2)
C(17)	5402(6)	-836(6)	2295(4)	43(2)
C(18)	5484(5)	61(6)	2534(3)	42(2)
C(19)	6255(5)	310(5)	3127(3)	36(2)
C(20)	9918(5)	2585(4)	3282(3)	33(2)

C(21)	10302(5)	2199(5)	2724(3)	47(2)
C(22)	11373(5)	2102(6)	2799(4)	60(2)
C(23)	12069(5)	2330(5)	3439(4)	48(2)
C(24)	11703(5)	2688(4)	3998(3)	38(2)
C(25)	10631(5)	2830(5)	3931(3)	38(2)
C(26)	8501(5)	3958(5)	4608(4)	32(2)
C(27)	8278(5)	4544(5)	4004(3)	38(2)
C(28)	8262(5)	5473(5)	4114(4)	44(2)
C(29)	8472(5)	5814(5)	4822(4)	47(2)
C(30)	8676(5)	5253(6)	5409(4)	46(2)
C(31)	8695(5)	4323(5)	5316(3)	36(2)
P(2)	6704(1)	2109(1)	1323(1)	31(1)
O(2)	7817(3)	2193(3)	1704(2)	32(1)
N(6)	6318(4)	1123(4)	939(2)	28(1)
N(7)	6199(4)	1608(4)	-626(2)	27(1)
N(8)	7291(5)	-2113(4)	1292(3)	39(2)
N(9)	5979(4)	2289(4)	1906(2)	30(1)
N(10)	6354(4)	2790(4)	616(2)	29(1)
C(32)	6891(5)	633(4)	469(3)	27(2)
C(33)	6274(5)	681(4)	-367(3)	26(2)
C(34)	5439(5)	1619(4)	-1350(3)	32(2)
C(35)	5760(5)	985(5)	-1937(3)	35(2)
C(36)	6855(5)	617(5)	-1568(3)	32(2)
C(37)	6809(5)	85(4)	-861(3)	30(2)
C(38)	7604(5)	1383(4)	-1325(3)	36(2)
C(39)	7202(5)	1937(5)	-754(3)	35(2)
C(40)	4961(5)	298(5)	-2226(4)	45(2)
C(41)	4445(5)	209(6)	-2920(4)	65(3)
C(42)	7036(5)	-336(5)	760(3)	27(2)
C(43)	7896(5)	-560(5)	1356(3)	32(2)
C(44)	7988(6)	-1467(5)	1594(3)	39(2)
C(45)	6486(5)	-1870(5)	747(3)	40(2)
C(46)	6342(5)	-985(5)	477(3)	32(2)
C(47)	8843(6)	-1710(5)	2170(3)	47(2)
C(48)	9559(6)	-1094(6)	2506(4)	47(2)
C(49)	9439(5)	-188(6)	2295(3)	46(2)
C(50)	8635(5)	68(5)	1731(3)	38(2)
C(51)	4898(5)	2486(4)	1766(3)	30(2)
C(52)	4503(5)	2937(5)	2300(3)	37(2)
C(53)	3444(5)	3122(5)	2185(3)	41(2)
C(54)	2764(5)	2908(5)	1535(3)	40(2)
C(55)	3137(5)	2459(4)	1003(3)	37(2)
C(56)	4183(5)	2244(4)	1099(3)	31(2)
C(57)	6563(5)	3715(5)	600(4)	32(2)
C(58)	6923(5)	4203(5)	1246(3)	36(2)
C(59)	7085(5)	5111(6)	1205(4)	48(2)
C(60)	6915(5)	5543(5)	536(4)	46(2)
C(61)	6576(5)	5060(5)	-98(4)	45(2)
C(62)	6397(5)	4140(5)	-72(3)	36(2)
C(63)	4841(5)	2964(5)	4713(3)	34(2)
C(64)	5043(5)	2205(5)	4356(3)	36(2)
C(65)	4736(5)	1379(6)	4558(3)	46(2)
C(66)	4193(5)	1306(5)	5112(3)	39(2)
C(67)	3998(5)	2083(6)	5481(3)	45(2)
C(68)	4319(5)	2885(5)	5270(3)	39(2)
C(69)	5181(6)	3865(5)	4470(4)	65(3)
C(70)	-49(6)	2767(5)	298(4)	43(2)
C(71)	-270(5)	1959(5)	591(3)	38(2)
C(72)	0(6)	1166(6)	311(4)	50(2)
C(73)	507(5)	1161(6)	-255(4)	46(2)
C(74)	725(5)	1958(6)	-563(3)	46(2)
C(75)	448(5)	2750(5)	-280(4)	44(2)

C(76)	-351(7)	3607(6)	617(4)	82(3)
C(77)	2868(5)	9345(6)	2820(4)	51(2)
C(78)	2798(6)	10159(6)	2416(4)	62(2)
C(79)	2231(7)	10234(6)	1700(5)	68(3)
C(80)	1703(7)	9460(7)	1361(4)	68(3)
C(81)	1749(6)	8688(6)	1736(4)	60(2)
C(82)	2330(5)	8614(6)	2440(4)	52(2)
C(83)	3535(5)	9273(5)	3564(3)	66(3)

Table 3. Anisotropic displacement parameters (A^2 x 10^3) for hk488.
The anisotropic displacement factor exponent takes the form:
-2 pi^2 [h^2 a*^2 U11 + ... + 2 h k a* b* U12]

	U11	U22	U33	U23	U13	U12
P(1)	31(1)	39(2)	23(1)	-2(1)	5(1)	-2(1)
O(1)	28(3)	41(3)	30(2)	-7(2)	8(2)	-3(2)
N(1)	35(4)	33(4)	31(3)	1(3)	17(3)	4(3)
C(2)	32(4)	35(5)	26(4)	-2(3)	6(3)	3(4)
N(3)	53(4)	37(4)	29(3)	-3(3)	8(3)	-1(4)
N(4)	20(3)	38(4)	30(3)	-4(3)	-1(2)	-3(3)
N(5)	40(4)	30(4)	19(3)	8(3)	1(3)	0(3)
C(1)	24(4)	31(5)	28(4)	-7(3)	5(3)	-3(4)
N(2)	33(4)	40(4)	17(3)	1(3)	1(3)	2(3)
C(3)	35(4)	37(5)	27(4)	2(3)	3(3)	-2(4)
C(4)	53(5)	43(6)	34(4)	-3(4)	-1(4)	7(4)
C(5)	44(5)	36(5)	29(4)	-4(4)	12(3)	-4(4)
C(6)	52(5)	31(5)	31(4)	0(4)	15(3)	1(4)
C(7)	36(5)	51(6)	43(4)	-10(4)	8(3)	-11(4)
C(8)	37(4)	53(5)	21(3)	-5(4)	-1(3)	-6(4)
C(9)	78(6)	63(7)	51(5)	9(5)	8(5)	18(6)
C(10)	102(8)	105(10)	89(7)	38(7)	13(6)	25(7)
C(11)	42(5)	40(6)	18(4)	-8(4)	15(3)	-7(4)
C(12)	37(4)	35(5)	29(4)	-12(4)	17(3)	-10(4)
C(13)	44(5)	57(7)	28(4)	-6(4)	15(4)	-7(5)
C(14)	50(5)	42(6)	39(4)	1(4)	24(4)	-3(4)
C(15)	44(5)	36(6)	31(4)	-12(4)	13(4)	-2(4)
C(16)	58(6)	47(6)	36(4)	-15(4)	18(4)	-24(5)
C(17)	37(5)	62(7)	28(4)	-10(5)	2(4)	-17(5)
C(18)	37(5)	63(7)	25(4)	7(4)	4(3)	-4(5)
C(19)	42(5)	39(5)	27(4)	-7(4)	7(3)	-5(4)
C(20)	35(4)	31(5)	31(4)	7(3)	7(3)	7(4)
C(21)	34(5)	65(6)	43(4)	-8(4)	12(3)	3(5)
C(22)	40(5)	86(7)	57(5)	-1(5)	16(4)	4(5)
C(23)	31(4)	62(7)	52(5)	-1(5)	12(4)	1(5)
C(24)	29(5)	33(5)	44(4)	6(4)	-7(3)	-7(4)
C(25)	36(5)	49(6)	28(4)	-6(4)	4(3)	-8(4)
C(26)	24(4)	32(5)	39(4)	-11(4)	6(3)	-5(4)
C(27)	39(5)	37(6)	34(4)	6(4)	4(3)	0(4)
C(28)	40(5)	44(6)	45(5)	1(4)	3(4)	-8(4)
C(29)	42(5)	35(6)	61(5)	-13(5)	7(4)	6(4)
C(30)	44(5)	46(6)	43(5)	-13(5)	4(4)	-9(5)
C(31)	35(5)	39(6)	32(4)	6(4)	4(3)	0(4)
P(2)	34(1)	32(1)	25(1)	-1(1)	6(1)	1(1)
O(2)	30(3)	35(3)	30(2)	-1(2)	5(2)	2(3)
N(6)	43(4)	21(4)	24(3)	4(3)	15(3)	-1(3)
N(7)	33(3)	26(4)	19(3)	5(3)	0(2)	1(3)
N(8)	56(4)	28(4)	37(3)	5(3)	20(3)	6(4)

N(9)	26(3)	39(4)	24(3)	0(3)	4(2)	1(3)
N(10)	34(3)	34(4)	17(3)	-10(3)	3(2)	2(3)
C(32)	28(4)	30(5)	20(3)	1(3)	-1(3)	2(4)
C(33)	27(4)	29(5)	20(3)	-2(3)	3(3)	1(3)
C(34)	48(5)	22(4)	22(4)	3(3)	0(3)	8(4)
C(35)	45(5)	39(5)	18(3)	1(4)	5(3)	-2(4)
C(36)	36(4)	32(5)	30(4)	5(3)	9(3)	1(4)
C(37)	37(4)	33(5)	20(3)	-6(3)	4(3)	2(4)
C(38)	42(4)	32(5)	36(4)	5(4)	13(3)	6(4)
C(39)	43(4)	37(5)	23(3)	10(3)	6(3)	-4(4)
C(40)	42(5)	47(6)	50(5)	-11(4)	21(4)	-7(4)
C(41)	68(6)	69(7)	44(5)	-20(5)	-14(4)	7(5)
C(42)	32(4)	25(5)	29(4)	2(4)	15(3)	7(4)
C(43)	34(4)	37(6)	27(4)	6(4)	7(3)	14(4)
C(44)	54(5)	40(6)	23(4)	9(4)	8(4)	14(5)
C(45)	52(5)	37(6)	31(4)	5(4)	11(4)	9(4)
C(46)	41(5)	31(5)	27(4)	11(4)	12(3)	21(4)
C(47)	71(6)	45(6)	29(4)	18(4)	18(4)	29(5)
C(48)	45(5)	62(7)	31(4)	14(5)	3(4)	21(5)
C(49)	54(5)	48(6)	33(4)	-3(4)	2(4)	3(5)
C(50)	40(5)	49(6)	23(4)	9(4)	3(3)	17(4)
C(51)	35(4)	24(5)	28(4)	7(3)	4(3)	2(4)
C(52)	40(5)	44(5)	26(4)	0(4)	7(3)	0(4)
C(53)	39(5)	52(6)	36(4)	2(4)	16(4)	-4(4)
C(54)	33(4)	39(5)	49(4)	9(4)	13(4)	13(4)
C(55)	32(4)	29(5)	43(4)	3(4)	-1(3)	-4(4)
C(56)	32(4)	25(5)	33(4)	-2(3)	-1(3)	1(4)
C(57)	27(4)	21(5)	48(5)	0(4)	8(3)	3(4)
C(58)	35(4)	32(6)	36(4)	-6(4)	2(3)	0(4)
C(59)	62(6)	34(6)	48(5)	-17(4)	9(4)	-6(5)
C(60)	47(5)	23(5)	75(6)	-9(5)	26(4)	-2(4)
C(61)	50(5)	37(6)	52(5)	11(4)	18(4)	3(4)
C(62)	46(5)	24(5)	37(4)	-4(4)	12(4)	2(4)
C(63)	31(4)	34(5)	34(4)	1(4)	-1(3)	3(4)
C(64)	34(4)	39(6)	33(4)	4(4)	8(3)	-1(4)
C(65)	33(5)	62(7)	40(4)	-5(4)	3(4)	11(5)
C(66)	27(4)	40(6)	49(4)	7(4)	6(4)	5(4)
C(67)	43(5)	51(6)	43(4)	1(5)	17(3)	-9(5)
C(68)	43(5)	35(6)	36(4)	0(4)	3(3)	9(4)
C(69)	78(6)	64(7)	58(5)	3(5)	24(4)	-18(6)
C(70)	43(5)	37(6)	48(5)	5(4)	9(4)	9(4)
C(71)	37(4)	43(6)	32(4)	2(4)	3(3)	4(4)
C(72)	49(5)	44(6)	55(5)	8(5)	6(4)	4(5)
C(73)	40(5)	53(6)	48(5)	-6(5)	16(4)	3(5)
C(74)	42(5)	56(6)	45(4)	11(5)	17(4)	11(5)
C(75)	42(5)	32(6)	57(5)	16(4)	8(4)	-3(4)
C(76)	111(8)	60(7)	76(6)	-3(5)	25(5)	20(6)
C(77)	38(5)	63(7)	53(5)	0(5)	15(4)	-5(5)
C(78)	54(6)	55(7)	78(6)	-7(6)	16(5)	-4(5)
C(79)	74(7)	55(7)	85(7)	28(6)	42(5)	33(6)
C(80)	82(7)	62(8)	58(6)	3(6)	15(5)	25(6)
C(81)	89(7)	39(6)	52(5)	-3(5)	17(5)	13(5)
C(82)	63(6)	41(6)	47(5)	6(5)	5(4)	0(5)
C(83)	82(6)	75(7)	42(4)	-1(5)	16(4)	-25(5)

Table 4. Hydrogen coordinates (x 10^4) and isotropic displacement parameters (A^2 x 10^3) for hk488.

	x	y	z	U(eq)

H(1X)	9060(30)	1200(40)	4030(30)	38
H(2)	9160	574	5372	37
H(4X)	8430(30)	2560(40)	2752(16)	37
H(5X)	8840(40)	2810(40)	4928(18)	37
H(1)	7204	1071	4409	33
H(3A)	9261	2251	6610	41
H(3B)	9934	1506	6313	41
H(4)	8774	1233	7342	54
H(5)	7489	190	6779	43
H(6A)	7124	36	5435	45
H(6B)	8219	-414	5838	45
H(7A)	6335	1229	6035	52
H(7B)	7030	1757	6730	52
H(8A)	6971	2049	5268	46
H(8B)	7573	2636	5955	46
H(9)	9790	-123	6691	78
H(10A)	10017	211	8143	120
H(10B)	10600	-552	7747	120
H(14)	8949	-2051	4296	50
H(15)	9100	-645	4806	44
H(16)	6021	-2053	2490	55
H(17)	4858	-1008	1885	52
H(18)	5001	492	2281	51
H(19)	6294	912	3290	43
H(21)	9829	1999	2286	56
H(22)	11624	1873	2398	72
H(23)	12799	2239	3492	57
H(24)	12185	2847	4445	46
H(25)	10393	3092	4326	46
H(27)	8137	4313	3516	45
H(28)	8107	5864	3702	53
H(29)	8473	6441	4898	56
H(30)	8809	5493	5895	55
H(31)	8839	3943	5736	43
H(6X)	5960(40)	890(40)	1210(30)	34
H(9X)	6410(30)	2370(40)	2314(17)	36
H(10X)	6210(40)	2540(30)	201(17)	34
H(32)	7595	912	521	33
H(33)	5549	451	-405	31
H(34A)	5373	2237	-1543	39
H(34B)	4745	1434	-1281	39
H(35)	5834	1363	-2364	42
H(36)	7115	226	-1921	39
H(37A)	7525	-78	-587	36
H(37B)	6403	-470	-998	36
H(38A)	7641	1759	-1755	43
H(38B)	8314	1153	-1102	43
H(39A)	7736	1930	-280	41
H(39B)	7111	2563	-926	41
H(40)	4806	-116	-1881	54
H(41A)	4575	608	-3285	78
H(41B)	3944	-254	-3057	78
H(45)	5986	-2309	531	48
H(46)	5750	-845	91	38
H(47)	8923	-2313	2328	57
H(48)	10142	-1275	2885	56
H(49)	9921	243	2549	55
H(50)	8569	676	1586	45
H(52)	4968	3119	2748	44
H(53)	3189	3402	2565	49

H(54)	2046	3065	1449	48
H(55)	2660	2293	556	44
H(56)	4420	1937	723	38
H(58)	7058	3914	1713	43
H(59)	7319	5443	1648	58
H(60)	7031	6167	515	55
H(61)	6461	5352	-563	55
H(62)	6161	3812	-516	43
H(64)	5396	2249	3968	43
H(65)	4897	858	4317	55
H(66)	3958	742	5239	47
H(67)	3648	2050	5871	53
H(68)	4177	3409	5516	47
H(69A)	5894	3818	4398	98
H(69B)	5166	4312	4852	98
H(69C)	4703	4045	4006	98
H(71)	-612	1953	987	46
H(72)	-165	618	512	61
H(73)	707	612	-435	55
H(74)	1060	1960	-963	56
H(75)	603	3298	-487	53
H(76A)	-1097	3724	412	123
H(76B)	64	4103	494	123
H(76C)	-220	3549	1155	123
H(78)	3157	10668	2652	75
H(79)	2192	10783	1440	81
H(80)	1312	9488	863	81
H(81)	1368	8187	1503	72
H(82)	2373	8052	2681	62
H(83A)	4251	9448	3558	99
H(83B)	3534	8656	3736	99
H(83C)	3269	9667	3897	99

Catalyst 14a

Identification code	hk561
Empirical formula	C28 H43 N6 P2 S2
Moiety formula	C28 H43 N6 P2 S2
Formula weight	589.74
Temperature	100(2) K
Wavelength	0.71073 A
Crystal system, space group	Orthorhombic, P212121
Unit cell dimensions	a = 9.517(4) A alpha = 90 deg.<br>b = 17.928(10) A beta = 90 deg.<br>c = 18.494(7) A gamma = 90 deg.
Volume	3155(3) A^3
Z, Calculated density	4, 1.241 Mg/m^3
Absorption coefficient	0.298 mm^-1

F(000)	1260
Crystal size	.15 x .07 x .03 mm
Theta range for data collection	1.58 to 24.83 deg.
Limiting indices	-10<=h<=7, -10<=k<=16, -14<=l<=21
Reflections collected / unique	4903 / 3207 [R(int) = 0.1096]
Reflection observed [I>2sigma(I)]	1436
Completeness to theta = 24.83	64.9 %
Absorption correction	None
Refinement method	Full-matrix least-squares on F^2
Data / restraints / parameters	3207 / 60 / 348
Goodness-of-fit on F^2	0.855
Final R indices [I>2sigma(I)]	R1 = 0.0694, wR2 = 0.1432
R indices (all data)	R1 = 0.1779, wR2 = 0.2066
Absolute structure parameter	-0.5(3)
Largest diff. peak and hole	0.287 and -0.278 e.A^-3

Table 2. Atomic coordinates (x 10^4) and equivalent isotropic displacement parameters (A^2 x 10^3) for z3_b.
U(eq) is defined as one third of the trace of the orthogonalized Uij tensor.

	x	y	z	U(eq)
S(1)	7102(4)	2367(3)	1662(2)	39(1)
S(2)	8459(4)	4377(3)	3466(2)	42(1)
P(1)	5974(4)	3074(3)	2190(2)	31(1)
P(2)	6694(4)	4138(3)	3034(2)	32(1)
N(1)	4295(10)	3013(7)	2070(6)	34(3)
N(2)	1935(11)	2482(10)	2692(5)	53(5)
N(3)	5490(10)	4721(8)	3293(6)	39(4)
N(4)	3678(12)	5114(10)	4378(6)	53(5)
C(1)	3548(12)	2428(11)	1657(6)	38(5)
C(2)	1991(13)	2486(10)	1902(6)	34(4)
C(3)	562(13)	2811(11)	2952(7)	56(6)
C(4)	2211(15)	1819(11)	3048(7)	55(6)
C(5)	1114(13)	1900(11)	1516(7)	55(6)
C(6)	1166(13)	2026(11)	695(7)	53(6)
C(7)	2709(16)	1950(13)	429(7)	67(7)
C(8)	3630(14)	2595(11)	823(6)	50(5)
N(9)	6530(10)	3959(7)	2144(5)	30(3)
C(10)	3962(14)	4705(10)	3130(6)	35(5)
C(11)	3306(14)	5277(11)	3616(7)	46(5)
C(12)	2896(16)	4527(11)	4721(8)	58(6)
C(13)	3628(16)	5808(11)	4810(7)	59(6)

C(14)	1698(13)	5347(9)	3483(6)	33(4)
C(15)	1416(12)	5553(10)	2704(6)	41(5)
C(16)	2082(13)	4959(9)	2189(7)	39(4)
C(17)	3681(11)	4924(9)	2339(7)	38(5)
N(18)	6136(10)	3238(7)	3089(5)	32(3)
C(19)	5824(13)	2774(10)	3690(7)	32(4)
C(20)	5367(14)	3094(11)	4337(7)	48(5)
C(21)	4939(18)	2609(15)	4899(8)	66(7)
C(22)	4977(15)	1865(13)	4816(8)	54(6)
C(23)	5398(14)	1563(11)	4185(8)	47(4)
C(24)	5851(13)	2006(10)	3614(7)	37(4)
C(25)	6966(14)	4396(13)	1532(7)	62(7)
C(26)	8135(14)	4803(9)	1541(7)	32(4)
C(27)	8531(16)	5243(11)	943(8)	55(6)
C(28)	7741(16)	5244(11)	312(8)	47(6)
C(29)	6544(16)	4828(11)	295(8)	50(5)
C(30)	6133(14)	4399(12)	895(7)	65(7)

Table 3. Anisotropic displacement parameters (A^2 x 10^3) for z3_b.
The anisotropic displacement factor exponent takes the form:
-2 pi^2 [h^2 a*^2 U11 + ... + 2 h k a* b* U12]

	U11	U22	U33	U23	U13	U12
S(1)	35(2)	36(4)	46(2)	-4(2)	1(2)	4(2)
S(2)	29(2)	52(4)	43(2)	-7(2)	0(2)	-8(2)
P(1)	27(2)	23(4)	45(3)	1(2)	0(2)	1(2)
P(2)	23(2)	34(4)	39(2)	-3(2)	-1(2)	-3(2)
N(1)	31(7)	28(12)	43(8)	-5(6)	2(6)	-7(7)
N(2)	30(7)	104(17)	26(7)	-13(8)	-4(6)	-15(9)
N(3)	19(7)	59(13)	39(8)	1(7)	4(6)	-12(7)
N(4)	47(9)	79(17)	33(8)	-9(8)	2(7)	21(9)
C(1)	25(8)	63(16)	26(8)	-6(8)	-8(7)	7(9)
C(2)	29(5)	32(6)	42(5)	-4(4)	0(4)	0(4)
C(3)	33(10)	49(18)	84(11)	-12(10)	10(8)	23(9)
C(4)	81(11)	53(14)	32(8)	29(8)	-7(8)	32(9)
C(5)	24(8)	100(20)	41(9)	17(10)	12(8)	30(10)
C(6)	21(9)	62(19)	76(12)	-3(10)	-7(8)	30(10)
C(7)	80(13)	70(20)	46(10)	-25(10)	-22(9)	-42(12)
C(8)	37(9)	62(18)	50(9)	8(9)	6(7)	5(10)
N(9)	23(6)	29(12)	39(7)	5(6)	0(6)	-4(6)
C(10)	40(10)	34(15)	31(9)	-2(8)	-12(7)	16(8)
C(11)	31(9)	72(18)	36(9)	-9(9)	-5(8)	-15(10)
C(12)	52(9)	55(15)	68(10)	33(9)	10(8)	-34(9)
C(13)	61(12)	70(20)	48(10)	7(10)	5(8)	12(11)
C(14)	31(5)	30(6)	39(5)	-5(4)	2(4)	0(4)
C(15)	26(9)	30(15)	65(10)	-28(9)	-5(7)	3(8)
C(16)	41(6)	36(6)	39(5)	1(4)	-3(4)	-2(5)
C(17)	9(8)	33(15)	72(11)	-17(9)	-15(7)	10(7)
N(18)	31(4)	33(6)	33(4)	-11(4)	-4(4)	-6(4)
C(19)	31(5)	32(6)	32(5)	3(4)	5(4)	-8(4)
C(20)	55(10)	45(17)	43(10)	-18(9)	7(8)	-23(10)
C(21)	100(16)	50(20)	45(11)	19(11)	27(10)	37(14)
C(22)	51(11)	60(20)	47(12)	11(10)	9(8)	10(12)
C(23)	48(6)	43(7)	51(6)	1(5)	0(4)	-2(5)
C(24)	39(5)	36(6)	36(5)	-8(4)	0(4)	11(5)
C(25)	28(9)	130(20)	32(9)	13(11)	10(8)	13(12)
C(26)	29(5)	31(6)	36(5)	3(4)	1(4)	-10(5)

C(27)	41(10)	70(20)	56(11)	-33(10)	10(9)	-13(10)
C(28)	49(11)	44(18)	48(12)	-11(9)	-3(9)	-4(10)
C(29)	35(10)	76(18)	38(10)	-7(9)	-3(8)	-9(11)
C(30)	32(9)	130(20)	33(10)	0(11)	-8(8)	-26(12)

Table 4. Hydrogen coordinates (x 10^4) and isotropic displacement parameters (A^2 x 10^3) for z3_b.

	x	y	z	U(eq)
H(1A)	3905	3008	2526	140(90)
H(3X)	5549	4733	3789	300(180)
H(1)	3940	1923	1768	45
H(2)	1638	2984	1740	41
H(3A)	-218	2490	2798	83
H(3B)	441	3310	2744	83
H(3C)	572	2845	3480	83
H(4A)	2430	1383	2779	66
H(4B)	2182	1800	3561	66
H(5A)	128	1928	1686	67
H(5B)	1479	1397	1632	67
H(6A)	804	2529	578	64
H(6B)	567	1653	448	64
H(7A)	2753	2012	-103	80
H(7B)	3084	1451	553	80
H(8A)	4617	2579	655	60
H(8B)	3238	3094	716	60
H(10)	3566	4199	3233	42
H(11)	3736	5770	3492	55
H(12A)	2029	4732	4926	87
H(12B)	3464	4305	5107	87
H(12C)	2663	4144	4363	87
H(13A)	2647	5954	4888	88
H(13B)	4120	6207	4550	88
H(13C)	4085	5725	5278	88
H(14A)	1234	4867	3597	40
H(14B)	1302	5734	3806	40
H(15A)	1821	6050	2599	49
H(15B)	389	5579	2620	49
H(16A)	1652	4465	2277	47
H(16B)	1912	5099	1679	47
H(17A)	4122	4554	2012	46
H(17B)	4108	5417	2239	46
H(20)	5343	3620	4397	57
H(21)	4620	2813	5344	79
H(22)	4705	1550	5204	64
H(23)	5384	1036	4129	57
H(24)	6175	1785	3178	44
H(26)	8715	4797	1959	38
H(27)	9351	5543	973	66
H(28)	8027	5527	-97	57
H(29)	5979	4827	-129	60
H(30)	5296	4112	875	78

Catalyst 15

Identification code	hk573
Empirical formula	C24 H52 N6 P2 S2
Moiety formula	C24 H52 N6 P2 S2
Formula weight	550.78
Temperature	100(2) K
Wavelength	0.71073 A
Crystal system, space group	Monoclinic, P21
Unit cell dimensions	a = 9.7823(2) A alpha = 90 deg.<br>b = 12.6943(3) A beta = 90.0540(10) deg.<br>c = 12.4351(3) A gamma = 90 deg.
Volume	1544.18(6) A^3
Z, Calculated density	2, 1.185 Mg/m^3
Absorption coefficient	0.299 mm^-1
F(000)	600
Crystal size	.25 x .15 x .1 mm
Theta range for data collection	1.64 to 26.99 deg.
Limiting indices	-12<=h<=12, -16<=k<=16, -11<=l<=15
Reflections collected / unique	9167 / 6637 [R(int) = 0.0225]
Reflection observed [I>2sigma(I)]	5735
Completeness to theta = 26.99	100.0 %
Absorption correction	None
Refinement method	Full-matrix least-squares on F^2
Data / restraints / parameters	6637 / 1 / 325
Goodness-of-fit on F^2	1.022
Final R indices [I>2sigma(I)]	R1 = 0.0341, wR2 = 0.0718
R indices (all data)	R1 = 0.0460, wR2 = 0.0852
Absolute structure parameter	-0.07(6)
Largest diff. peak and hole	0.220 and -0.250 e.A^-3

Table 2. Atomic coordinates (x 10^4) and equivalent isotropic

displacement parameters (A^2 x 10^3) for zz_b.
U(eq) is defined as one third of the trace of the orthogonalized Uij tensor.

	x	y	z	U(eq)
S(1)	-1145(1)	-2023(1)	8759(1)	24(1)
S(2)	-930(1)	-839(1)	5018(1)	23(1)
P(1)	37(1)	-956(1)	8151(1)	16(1)
P(2)	146(1)	-342(1)	6226(1)	16(1)
N(1)	1045(2)	-434(2)	9056(2)	19(1)
N(2)	3654(2)	-345(2)	9947(2)	29(1)
N(3)	1172(2)	583(2)	5808(2)	18(1)
N(4)	3346(2)	1219(2)	4531(2)	20(1)
C(1)	1167(2)	-730(2)	10200(2)	19(1)
C(2)	2459(2)	-211(2)	10662(2)	21(1)
C(3)	4269(3)	-1393(3)	9988(2)	42(1)
C(4)	4680(3)	476(3)	10153(3)	48(1)
C(5)	2664(2)	-589(2)	11819(2)	24(1)
C(6)	1442(2)	-284(2)	12521(2)	28(1)
C(7)	118(2)	-700(2)	12035(2)	26(1)
C(8)	-48(2)	-359(2)	10869(2)	22(1)
N(5)	966(2)	-1206(2)	7039(2)	17(1)
C(10)	2330(2)	1095(2)	6362(2)	18(1)
C(11)	3051(2)	1791(2)	5529(2)	18(1)
C(12)	4519(2)	506(2)	4596(2)	28(1)
C(13)	3465(3)	1919(2)	3603(2)	29(1)
C(14)	4262(2)	2367(2)	6044(2)	24(1)
C(15)	3822(2)	3022(2)	7020(2)	29(1)
C(16)	3082(2)	2337(2)	7833(2)	28(1)
C(17)	1872(2)	1770(2)	7306(2)	22(1)
N(6)	-650(2)	6(2)	7372(1)	16(1)
C(19)	-1907(2)	655(2)	7527(2)	20(1)
C(20)	-1895(2)	1553(2)	6709(2)	23(1)
C(21)	-3185(2)	-22(2)	7363(2)	26(1)
C(22)	-1881(2)	1100(2)	8669(2)	25(1)
C(23)	1749(2)	-2175(2)	6731(2)	21(1)
C(24)	2668(2)	-1892(2)	5789(2)	28(1)
C(25)	2621(3)	-2499(2)	7699(2)	33(1)
C(26)	785(3)	-3064(2)	6426(2)	33(1)

Table 3. Anisotropic displacement parameters (A^2 x 10^3) for zz_b.
The anisotropic displacement factor exponent takes the form:
-2 pi^2 [h^2 a*^2 U11 + ... + 2 h k a* b* U12]

	U11	U22	U33	U23	U13	U12
S(1)	26(1)	23(1)	22(1)	2(1)	3(1)	-8(1)
S(2)	26(1)	27(1)	17(1)	-1(1)	-4(1)	-9(1)
P(1)	17(1)	18(1)	15(1)	0(1)	1(1)	-2(1)
P(2)	17(1)	18(1)	15(1)	0(1)	-1(1)	-2(1)
N(1)	18(1)	25(1)	16(1)	4(1)	0(1)	-6(1)
N(2)	19(1)	47(2)	22(1)	2(1)	1(1)	-4(1)
N(3)	18(1)	21(1)	14(1)	0(1)	-1(1)	-4(1)
N(4)	18(1)	22(1)	20(1)	2(1)	2(1)	-1(1)

C(1)	23(1)	19(1)	14(1)	3(1)	0(1)	-0(1)
C(2)	21(1)	24(1)	19(1)	-1(1)	-1(1)	-0(1)
C(3)	30(1)	72(2)	22(2)	-5(2)	-1(1)	18(2)
C(4)	30(2)	81(3)	34(2)	6(2)	-2(1)	-23(2)
C(5)	24(1)	30(2)	18(1)	-0(1)	-3(1)	2(1)
C(6)	31(1)	37(2)	16(1)	-3(1)	1(1)	2(1)
C(7)	28(1)	36(2)	16(1)	-1(1)	4(1)	-1(1)
C(8)	21(1)	27(1)	17(1)	1(1)	1(1)	-1(1)
N(5)	21(1)	17(1)	13(1)	0(1)	1(1)	2(1)
C(10)	16(1)	17(1)	21(1)	1(1)	-5(1)	-2(1)
C(11)	15(1)	18(1)	20(1)	0(1)	-1(1)	-1(1)
C(12)	28(1)	24(2)	32(2)	-1(1)	5(1)	1(1)
C(13)	29(1)	36(2)	22(1)	5(1)	1(1)	-4(1)
C(14)	19(1)	26(2)	27(1)	1(1)	-0(1)	-6(1)
C(15)	26(1)	30(2)	30(2)	-6(1)	-2(1)	-11(1)
C(16)	29(1)	34(2)	23(1)	-5(1)	-4(1)	-6(1)
C(17)	23(1)	22(1)	22(1)	-2(1)	1(1)	-3(1)
N(6)	16(1)	19(1)	14(1)	1(1)	0(1)	1(1)
C(19)	14(1)	23(1)	22(1)	2(1)	1(1)	1(1)
C(20)	19(1)	23(1)	27(1)	3(1)	-0(1)	4(1)
C(21)	17(1)	32(2)	29(1)	2(1)	2(1)	-1(1)
C(22)	22(1)	31(2)	23(1)	-2(1)	5(1)	5(1)
C(23)	25(1)	16(1)	24(1)	-2(1)	4(1)	4(1)
C(24)	29(1)	24(2)	29(2)	-4(1)	7(1)	4(1)
C(25)	39(1)	32(2)	29(2)	-1(1)	-1(1)	15(1)
C(26)	36(1)	21(2)	41(2)	-7(1)	7(1)	-3(1)

Table 4. Hydrogen coordinates (x 10^4) and isotropic displacement parameters (A^2 x 10^3) for zz_b.

	x	y	z	U(eq)
H(1)	1251	-1513	10259	22
H(2)	2271	562	10701	25
H(3A)	4939	-1465	9407	62
H(3B)	3554	-1928	9902	62
H(3C)	4726	-1491	10683	62
H(4A)	5059	384	10877	72
H(4B)	4250	1171	10098	72
H(4C)	5416	420	9622	72
H(5A)	3507	-272	12118	29
H(5B)	2775	-1365	11825	29
H(6A)	1563	-579	13252	34
H(6B)	1393	493	12583	34
H(7A)	114	-1478	12074	32
H(7B)	-666	-436	12459	32
H(8A)	-117	418	10832	26
H(8B)	-902	-660	10572	26
H(10)	2979	543	6624	22
H(11)	2378	2351	5331	21
H(12A)	5366	918	4610	42
H(12B)	4523	40	3967	42
H(12C)	4454	82	5252	42
H(13A)	4258	2382	3698	43
H(13B)	2635	2346	3539	43
H(13C)	3584	1498	2949	43
H(14A)	4689	2836	5504	29
H(14B)	4956	1845	6272	29

H(15A)	4637	3342	7361	34
H(15B)	3210	3599	6782	34
H(16A)	2749	2779	8433	34
H(16B)	3725	1810	8131	34
H(17A)	1415	1319	7847	27
H(17B)	1200	2299	7052	27
H(20A)	-1051	1961	6789	34
H(20B)	-2685	2013	6832	34
H(20C)	-1942	1261	5980	34
H(21A)	-3197	-297	6627	39
H(21B)	-4002	409	7484	39
H(21C)	-3174	-610	7874	39
H(22A)	-1871	518	9187	38
H(22B)	-2695	1534	8786	38
H(22C)	-1059	1531	8765	38
H(24A)	2106	-1655	5183	41
H(24B)	3198	-2512	5575	41
H(24C)	3292	-1325	6004	41
H(25A)	3250	-1926	7883	50
H(25B)	3146	-3133	7519	50
H(25C)	2025	-2647	8314	50
H(26A)	172	-3214	7030	49
H(26B)	1316	-3696	6255	49
H(26C)	243	-2856	5798	49
H(2X)	1750(20)	-210(20)	8860(20)	23(7)
H(1X)	1270(20)	590(20)	5160(20)	23(7)

Catalyst 16

Identification code	hk568
Empirical formula	C22 H41 N5 P2 S2
Moiety formula	C22 H41 N5 P2 S2
Formula weight	501.66
Temperature	100(2) K
Wavelength	0.71073 A
Crystal system, space group	Orthorhombic, P212121
Unit cell dimensions	a = 10.4209(3) A alpha = 90 deg.<br>b = 22.6478(7) A beta = 90 deg.<br>c = 23.1679(4) A gamma = 90 deg.
Volume	5467.9(2) A^3
Z, Calculated density	8, 1.219 Mg/m^3
Absorption coefficient	0.331 mm^-1
F(000)	2160
Crystal size	.3 x .2 x .1 mm
Theta range for data collection	1.26 to 27.00 deg.

Limiting indices	-13<=h<=13, -28<=k<=28, -29<=l<=29
Reflections collected / unique	11888 / 11888 [R(int) = 0.0000]
Reflection observed [I>2sigma(I)]	8809
Completeness to theta = 27.00	100.0 %
Absorption correction	None
Refinement method	Full-matrix least-squares on F^2
Data / restraints / parameters	11888 / 0 / 575
Goodness-of-fit on F^2	1.035
Final R indices [I>2sigma(I)]	R1 = 0.0422, wR2 = 0.0754
R indices (all data)	R1 = 0.0711, wR2 = 0.1009
Absolute structure parameter	-0.01(6)
Largest diff. peak and hole	0.303 and -0.283 e.A^-3

Table 2. Atomic coordinates (x 10^4) and equivalent isotropic displacement parameters (A^2 x 10^3) for z3_b.
U(eq) is defined as one third of the trace of the orthogonalized Uij tensor.

	x	y	z	U(eq)
S(1)	3460(1)	301(1)	4144(1)	20(1)
S(2)	2963(1)	1221(1)	5953(1)	22(1)
P(1)	1843(1)	577(1)	4450(1)	15(1)
P(2)	1576(1)	1072(1)	5411(1)	16(1)
N(1)	1759(3)	1274(1)	4720(1)	15(1)
N(2)	1386(3)	395(1)	5133(1)	14(1)
N(3)	229(3)	1307(1)	5699(1)	17(1)
N(4)	639(3)	440(1)	4011(1)	16(1)
N(5)	-1273(3)	-355(1)	3686(1)	18(1)
C(1)	1392(3)	-190(1)	5439(1)	18(1)
C(2)	2757(3)	-369(2)	5590(1)	26(1)
C(3)	786(3)	-644(2)	5035(1)	22(1)
C(4)	604(3)	-120(2)	5994(1)	23(1)
C(5)	-1060(3)	1189(2)	5539(1)	17(1)
C(6)	-1442(3)	1162(1)	4959(1)	19(1)
C(7)	-2708(3)	1039(2)	4827(1)	25(1)
C(8)	-3611(4)	957(2)	5255(1)	24(1)
C(9)	-3244(3)	994(2)	5828(1)	25(1)
C(10)	-1971(3)	1105(2)	5971(1)	22(1)
C(11)	2194(3)	1859(2)	4485(1)	20(1)
C(12)	3657(4)	1907(2)	4504(1)	30(1)
C(13)	1578(4)	2342(2)	4848(1)	31(1)
C(14)	1723(4)	1911(2)	3861(1)	29(1)
C(15)	724(3)	171(2)	3427(1)	16(1)
C(16)	-633(3)	1(2)	3239(1)	20(1)
C(17)	-823(4)	-966(2)	3708(1)	32(1)
C(18)	-2657(4)	-342(2)	3627(2)	34(1)

C(19)	-604(3)	-267(2)	2631(1)	23(1)
C(20)	7(4)	146(2)	2194(1)	26(1)
C(21)	1339(3)	331(2)	2385(1)	24(1)
C(22)	1290(3)	603(2)	2989(1)	21(1)
S(3)	4840(1)	1928(1)	1219(1)	20(1)
S(4)	4875(1)	2921(1)	3067(1)	22(1)
P(3)	6331(1)	2109(1)	1682(1)	15(1)
P(4)	6340(1)	2616(1)	2647(1)	16(1)
N(6)	6552(2)	2798(1)	1951(1)	14(1)
N(7)	6300(3)	1925(1)	2394(1)	15(1)
N(8)	7658(2)	2737(1)	3016(1)	17(1)
N(9)	7676(3)	1900(1)	1387(1)	16(1)
N(10)	9889(3)	2198(1)	777(1)	21(1)
C(23)	5842(3)	1370(2)	2686(1)	19(1)
C(24)	6333(3)	1384(2)	3307(1)	24(1)
C(25)	6418(4)	842(2)	2368(1)	24(1)
C(26)	4382(3)	1337(2)	2681(1)	28(1)
C(27)	8954(3)	2672(2)	2823(1)	15(1)
C(28)	9820(3)	3133(2)	2891(1)	19(1)
C(29)	11077(3)	3078(2)	2710(1)	21(1)
C(30)	11490(3)	2566(2)	2448(1)	22(1)
C(31)	10633(3)	2100(2)	2379(1)	21(1)
C(32)	9374(3)	2151(2)	2570(1)	19(1)
C(33)	6441(3)	3384(1)	1662(1)	18(1)
C(34)	7170(4)	3354(2)	1095(1)	26(1)
C(35)	5039(4)	3539(2)	1556(1)	27(1)
C(36)	7057(4)	3845(2)	2057(1)	27(1)
C(37)	7890(3)	1599(2)	831(1)	17(1)
C(38)	9335(3)	1605(2)	711(1)	19(1)
C(39)	11283(3)	2183(2)	864(1)	30(1)
C(40)	9580(4)	2596(2)	300(1)	31(1)
C(41)	9611(3)	1293(2)	130(1)	23(1)
C(42)	9116(3)	657(2)	147(1)	27(1)
C(43)	7689(3)	648(2)	274(1)	28(1)
C(44)	7404(3)	965(2)	842(1)	21(1)

Table 3. Anisotropic displacement parameters (A^2 x 10^3) for z3_b.
The anisotropic displacement factor exponent takes the form:
-2 pi^2 [h^2 a*^2 U11 + ... + 2 h k a* b* U12]

	U11	U22	U33	U23	U13	U12
S(1)	16(1)	23(1)	19(1)	-3(1)	3(1)	2(1)
S(2)	17(1)	28(1)	21(1)	-7(1)	-3(1)	-0(1)
P(1)	14(1)	14(1)	15(1)	-1(1)	1(1)	-1(1)
P(2)	14(1)	17(1)	15(1)	-2(1)	0(1)	-0(1)
N(1)	19(2)	14(2)	13(1)	-1(1)	2(1)	-3(1)
N(2)	16(2)	13(2)	12(1)	-1(1)	1(1)	-3(1)
N(3)	13(2)	25(2)	14(1)	-7(1)	0(1)	3(1)
N(4)	13(2)	19(2)	15(1)	-3(1)	3(1)	-4(1)
N(5)	20(2)	20(2)	16(1)	1(1)	-1(1)	-6(1)
C(1)	20(2)	16(2)	17(2)	3(1)	2(2)	1(2)
C(2)	24(2)	25(2)	29(2)	7(2)	2(2)	6(2)
C(3)	26(2)	16(2)	25(2)	1(2)	4(2)	-1(2)
C(4)	27(2)	23(2)	19(2)	3(2)	4(2)	0(2)
C(5)	14(2)	16(2)	21(2)	-3(2)	0(1)	1(2)
C(6)	21(2)	19(2)	18(2)	-1(1)	1(2)	4(2)
C(7)	24(2)	26(2)	25(2)	-5(2)	-8(2)	5(2)

C(8)	13(2)	22(2)	38(2)	-2(2)	-5(2)	3(2)
C(9)	21(2)	28(2)	26(2)	-3(2)	3(2)	-1(2)
C(10)	20(2)	26(2)	19(2)	-3(2)	-1(2)	-0(2)
C(11)	25(2)	12(2)	23(2)	2(2)	1(2)	-3(2)
C(12)	30(2)	28(2)	31(2)	2(2)	3(2)	-12(2)
C(13)	43(3)	16(2)	33(2)	-3(2)	5(2)	-1(2)
C(14)	40(3)	22(2)	24(2)	6(2)	-2(2)	-5(2)
C(15)	17(2)	18(2)	14(2)	-3(1)	-0(1)	-0(2)
C(16)	19(2)	20(2)	21(2)	0(2)	-1(2)	0(2)
C(17)	46(3)	23(2)	27(2)	1(2)	2(2)	-6(2)
C(18)	29(2)	42(3)	32(2)	9(2)	-3(2)	-10(2)
C(19)	26(2)	23(2)	20(2)	-5(2)	-3(2)	-3(2)
C(20)	30(2)	33(2)	15(2)	-5(2)	-4(2)	-1(2)
C(21)	25(2)	30(2)	18(2)	-1(2)	3(2)	1(2)
C(22)	20(2)	26(2)	18(2)	-1(2)	1(2)	1(2)
S(3)	17(1)	25(1)	18(1)	-4(1)	-3(1)	-0(1)
S(4)	17(1)	30(1)	21(1)	-9(1)	2(1)	1(1)
P(3)	14(1)	17(1)	13(1)	-1(1)	1(1)	0(1)
P(4)	14(1)	19(1)	14(1)	-3(1)	1(1)	-0(1)
N(6)	16(2)	14(2)	13(1)	1(1)	-0(1)	0(1)
N(7)	19(2)	15(2)	11(1)	0(1)	1(1)	-2(1)
N(8)	16(2)	19(2)	16(1)	-5(1)	2(1)	-1(1)
N(9)	16(2)	18(2)	13(1)	-5(1)	-3(1)	-2(1)
N(10)	20(2)	20(2)	21(1)	5(1)	6(1)	-2(1)
C(23)	20(2)	19(2)	17(2)	3(1)	1(2)	-2(2)
C(24)	30(2)	25(2)	17(2)	2(1)	4(2)	-4(2)
C(25)	27(2)	20(2)	24(2)	1(2)	2(2)	1(2)
C(26)	21(2)	30(2)	32(2)	3(2)	5(2)	-8(2)
C(27)	13(2)	21(2)	10(2)	-0(1)	-2(1)	3(2)
C(28)	21(2)	21(2)	15(2)	-2(1)	-3(1)	-2(2)
C(29)	18(2)	25(2)	20(2)	4(2)	-2(2)	-3(2)
C(30)	12(2)	33(2)	22(2)	7(2)	-0(2)	2(2)
C(31)	23(2)	24(2)	16(2)	0(2)	3(2)	9(2)
C(32)	17(2)	21(2)	18(2)	2(2)	-4(1)	-1(2)
C(33)	19(2)	14(2)	22(2)	5(1)	1(2)	3(2)
C(34)	31(2)	24(2)	23(2)	6(2)	1(2)	2(2)
C(35)	28(2)	20(2)	34(2)	6(2)	-2(2)	6(2)
C(36)	35(2)	16(2)	29(2)	1(2)	-1(2)	-1(2)
C(37)	18(2)	21(2)	12(2)	-1(1)	-2(1)	3(2)
C(38)	21(2)	21(2)	15(2)	-0(1)	1(1)	6(2)
C(39)	22(2)	32(2)	35(2)	-1(2)	4(2)	-7(2)
C(40)	34(3)	30(2)	28(2)	8(2)	8(2)	3(2)
C(41)	19(2)	29(2)	20(2)	-2(2)	2(2)	3(2)
C(42)	23(2)	37(3)	22(2)	-13(2)	4(2)	4(2)
C(43)	25(2)	28(2)	32(2)	-10(2)	0(2)	3(2)
C(44)	19(2)	25(2)	19(2)	-6(2)	0(2)	3(2)

Table 4. Hydrogen coordinates (x 10^4) and isotropic displacement parameters (A^2 x 10^3) for z3_b.

	x	y	z	U(eq)
H(3X)	332	1432	6079	21
H(4X)	-16	314	4196	19
H(2A)	3259	-414	5235	39
H(2B)	2744	-744	5800	39
H(2C)	3148	-64	5833	39
H(3A)	-67	-507	4917	33

H(3B)	708	-1023	5235	33
H(3C)	1328	-692	4692	33
H(4A)	1017	171	6246	34
H(4B)	554	-501	6193	34
H(4C)	-264	14	5896	34
H(6)	-836	1228	4660	23
H(7)	-2961	1011	4434	30
H(8)	-4478	875	5157	29
H(9)	-3864	944	6125	30
H(10)	-1722	1124	6365	26
H(12A)	3951	1870	4904	45
H(12B)	3922	2290	4349	45
H(12C)	4035	1590	4271	45
H(13A)	642	2314	4818	46
H(13B)	1859	2729	4707	46
H(13C)	1836	2295	5252	46
H(14A)	2077	1586	3631	43
H(14B)	2007	2289	3699	43
H(14C)	784	1892	3853	43
H(15)	1272	-191	3445	20
H(16)	-1128	378	3208	24
H(17A)	-1188	-1163	4047	48
H(17B)	116	-971	3734	48
H(17C)	-1094	-1174	3357	48
H(18A)	-2957	68	3635	52
H(18B)	-3049	-562	3946	52
H(18C)	-2902	-524	3259	52
H(19A)	-117	-642	2641	27
H(19B)	-1492	-357	2508	27
H(20A)	62	-56	1816	31
H(20B)	-538	500	2147	31
H(21A)	1690	623	2108	29
H(21B)	1914	-17	2387	29
H(22A)	2167	715	3110	26
H(22B)	760	966	2979	26
H(8X)	7550	3041	3258	21
H(9X)	8311	2146	1493	19
H(24A)	7273	1404	3306	36
H(24B)	6057	1025	3508	36
H(24C)	5986	1731	3505	36
H(25A)	6103	836	1969	35
H(25B)	6164	476	2563	35
H(25C)	7356	874	2367	35
H(26A)	4027	1685	2875	41
H(26B)	4103	979	2884	41
H(26C)	4075	1325	2281	41
H(28)	9544	3491	3066	23
H(29)	11661	3395	2767	25
H(30)	12350	2531	2315	27
H(31)	10910	1745	2200	25
H(32)	8799	1829	2528	23
H(34A)	8064	3244	1170	38
H(34B)	7143	3740	905	38
H(34C)	6773	3057	843	38
H(35A)	4643	3233	1315	41
H(35B)	4985	3921	1359	41
H(35C)	4587	3562	1926	41
H(36A)	6618	3845	2431	40
H(36B)	6983	4236	1879	40
H(36C)	7965	3749	2113	40
H(37)	7437	1820	518	20
H(38)	9739	1354	1017	23

H(39A)	11487	1913	1182	44
H(39B)	11590	2580	960	44
H(39C)	11703	2045	510	44
H(40A)	10067	2479	-44	46
H(40B)	9806	3001	407	46
H(40C)	8659	2574	216	46
H(41A)	9185	1510	-187	27
H(41B)	10546	1292	55	27
H(42A)	9281	464	-229	33
H(42B)	9580	434	449	33
H(43A)	7388	234	299	34
H(43B)	7219	844	-44	34
H(44A)	6467	965	912	25
H(44B)	7820	750	1164	25

References

[1] Yang, Y.; Chen, G.; Zheng, L. *Lett. Org. Chem.* **2010**, 7, 2, 163

[2] Oliva, C. G.; Silva, A. M-S.; Resende, D.; Paz, F. A. A.; Cavaleiro, J. A. S. *Eur. J. Org. Chem.* **2010**, 3449-3458

[3] Taniguchi, T.; Fukuba, T.; Nakatsuka, S.; Hayase, S.; Kawatsura, M.; Uno, H.; Itoh, T. *J. Org. Chem.* **2008**, 73 (10), 3875

[4] Zhmurova, I. N.; Kirsanov, A. V. *J. Gen. Chem. USSR (Engl. Transl.)* **1960**, 30,3044

[5] Bashall, A.; Doyle, E. L.; Wright, D. S. *Chem. Comm.* **2001**, 2542-2543
